# Prosthesis usability experience is associated with extent of upper limb prosthesis adoption: A Structural Equation Modeling (SEM) analysis

**DOI:** 10.1371/journal.pone.0299155

**Published:** 2024-06-25

**Authors:** Linda J. Resnik, Matthew Borgia, Emily L. Graczyk, Jessica Barth, Pengsheng Ni

**Affiliations:** 1 Providence VA Medical Center, Providence, Rhode Island, United States of America; 2 Department of Health Services, Policy and Practice, Brown University School of Public Health, Providence, Rhode Island, United States of America; 3 Department of Biomedical Engineering, Case Western Reserve University, Cleveland, Ohio, United States of America; 4 Louis Stokes Cleveland Department of Veterans Affairs Medical Center, Cleveland, Ohio, United States of America; 5 Center for Innovation in Long-Term Services & Supports, Providence VA Medical Center, Providence, Rhode Island, United States of America; 6 Biostatistics & Epidemiology Data Analytic Center, Department of Health Law, Policy and Management, Boston University School of Public Health, Boston, Massachusetts, United States of America; UCSI University Kuala Lumpur Campus: UCSI University, ISLAMIC REPUBLIC OF IRAN

## Abstract

Factors associated with upper limb prosthesis adoption are not well understood. In this study, we explored how prosthesis usability experience relates to the extent of prosthesis adoption through the development of a structural equation model (SEM). First, items related to prosthesis usability were developed and refined using cognitive testing and pilot testing and employed in a survey of 402 prosthesis users (mean age 61.7 (sd 14.4), 77.1% Veterans). The SEM examined two unidimensional latent constructs: Prosthesis Usability Experience and Prosthesis Adoption–and each had multiple measured indicators. SEMs tested direct as well as moderating and mediating effects between the latent constructs and covariates related to demographics and prosthesis type. SEM found a significant positive association between Prosthesis Usability Experience and Extent of Prosthesis Adoption. Several covariates had direct effects on prosthesis adoption: 1) Extent of Prosthesis Adoption was lower for those with transhumeral and shoulder amputation, and higher for those with bilateral amputation, compared to the reference group with unilateral transradial amputation and 2) Myoelectric multiple degree of freedom (multi-DOF) prosthesis use was associated with lower Extent of Prosthesis Adoption, compared to body-powered prosthesis use. Myoelectric multi-DOF use also modified the effect of Prosthesis Usability Experience on Extent of Prosthesis Adoption. For those with bilateral ULA, the strength of the relationship between Prosthesis Usability Experience and Extent of Prosthesis Adoption was reduced. Findings suggest that in order to increase prosthesis adoption, prosthetics developers and rehabilitation providers should focus on implementing strategies to improve prosthesis usability experience. New Prosthesis Usability Experience measures could be used to identify persons at greater risk for poor prosthesis adoption and target interventions to increase prosthesis use.

## Introduction

While considerable research has been conducted to understand factors associated with upper limb prosthesis rejection and abandonment [1–7], far less research has been conducted to understand factors associated with the extent of prosthesis adoption. The extent of prosthesis adoption is sometimes considered a marker of successful prosthetic rehabilitation and is an outcome of interest to both rehabilitation care providers and prosthesis technology developers. For example, some clinicians believe that the goal is for the prosthesis “to become an integrated part of the client’s life.” [8].

Previous studies have used a variety of approaches to estimate the extent of prosthesis adoption, including hours of prosthesis use, frequency of prosthesis use, self-reported activity performance, and a variety of data and video logs [9–21]. Each approach provides a lens through which the extent of prosthesis adoption can be quantified, but no single measure provides a complete picture of this outcome. In addition, because different studies define and quantify extent of prosthesis adoption in different ways, it can be difficult to synthesize findings across studies. Furthermore, several studies examine factors associated with prosthesis satisfaction [13, 22–25] which, while important, is a different construct than extent of adoption. Therefore, to improve interpretability of findings, a composite measure that combines several indicator variables is needed to provide a more robust estimate of the extent of prosthesis adoption. In this paper, we used a novel method of estimating the latent construct of Extent of Prosthesis Adoption using multiple indicators of prosthesis use including frequency, duration, and engagement in everyday tasks. In addition, we employed a structural equation model (SEM) to estimate relationships of multiple variables simultaneously within a single statistical structure.

Prior studies have demonstrated the impact of level of congenital limb difference or acquired limb loss (referred to as amputation in this paper) and laterality (unilateral or bilateral) on prosthesis adoption. In general, people with bilateral upper limb amputation (ULA) use prostheses more than people with unilateral ULA. While the trends are largely consistent across metrics, the magnitude of impact differs based on which metric of prosthesis adoption is selected. Using hours of use per day as a metric, prior studies have demonstrated 52% of persons with unilateral ULA and 76% of those with bilateral ULA use their devices at least 8 hours a day [11, 12, 15, 26]. However, with frequency of use as a metric, prior research reported that 70% of those with unilateral ULA and more than 95% of those with bilateral ULA use their prostheses daily [15]. Amputation level also contributes to prosthesis use–people with transradial (TR) amputation tend to use their prostheses more than people with more proximal levels of amputation (transhumeral (TH) or shoulder (SH)) [10, 11]. Using the number of tasks performed with the prosthesis as a metric, prior studies demonstrated that persons with TR unilateral ULA engaged their prosthesis in an average of 28% of unilateral tasks and 43% of bilateral tasks as compared to those with TH (14% and 26%) and SH level amputation (10% and 22%), respectively [2]. Variability in magnitude of effect by amputation level also exists across metrics.

While it is possible that the same variables that lead to prosthesis rejection might promote adoption when the direction of the factor is reversed, it is also possible that an entirely different set of factors contributes to prosthesis adoption. Factors beyond bodily impairment have been associated with prosthesis abandonment and may also contribute to the extent of prosthesis adoption. Prior studies have shown that prosthesis abandonment rates differ by device type [5], and factors related to device usability experience have been identified as reasons persons with ULA abandon or avoid using a prosthesis. These usability factors include lack of prosthesis functionality and reliability [3, 5], dissatisfaction with prosthesis cosmesis, dissatisfaction with technology, discomfort associated with wearing a prosthesis, complexity of control, difficulty in prosthesis use, lack of sensory feedback, and the mental workload required for prosthesis use [3, 15, 25, 27]. These device usability factors may be modulated by participant demographics or characteristics. For example, women with ULA are less likely to be prosthesis users than men overall [2], and women may prefer anthropomorphic prosthetic devices [3]. With so many factors influencing prosthesis use, it is difficult to prioritize technologies and therapies to meet the needs of persons with limb loss. A better understanding of the prosthesis usability experience and its influence on the extent of prosthesis adoption may provide opportunities to tailor amputation rehabilitation interventions to a specific patient to improve outcomes.

Therefore, the purposes of this study were two-fold: 1) to develop new patient reported measures of the prosthesis usability experience and 2) to develop a structural equation model (SEM) explaining the relationship between prosthesis usability and a composite measure of the latent construct of upper limb prosthesis adoption. These novel measures of prosthesis usability and the new understanding of the relationship between prosthesis usability and extent of prosthesis adoption can provide guidance to clinicians, researchers, payers, and device developers.

## Materials and methods

### Overview of study design

**Fig 1:**
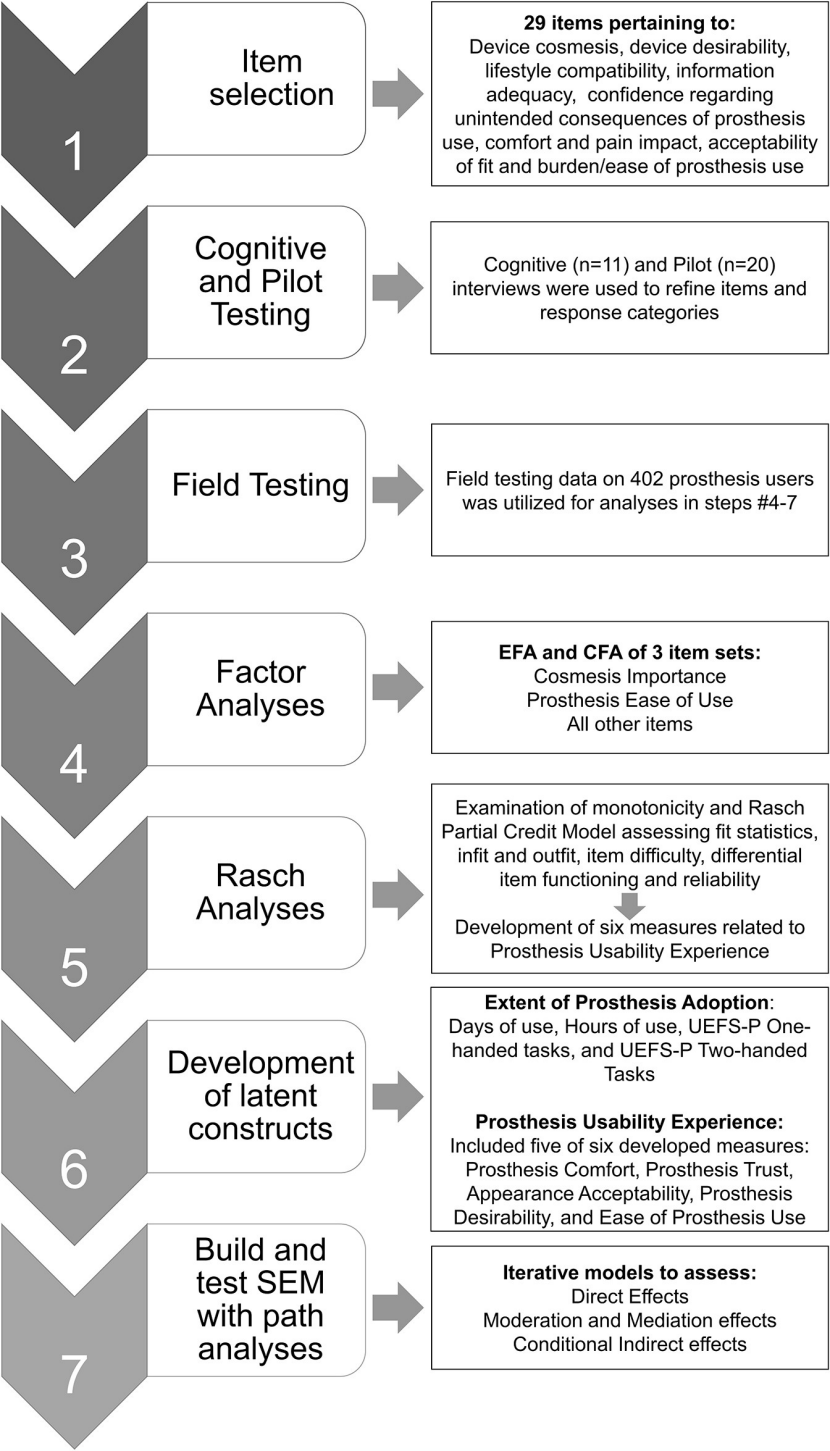
Study design flow chart.

The study design is shown in Fig 1. First, we developed and refined new measures related to prosthesis usability using cognitive testing, pilot testing, factor and Rasch analyses. Then we used these measures to estimate the latent construct of Prosthesis Usability and developed a latent construct for Extent of Prosthesis Adoption. Finally, we employed these two latent constructs in an SEM that examined the relationships between Prosthesis Usability Experience, Extent of Prosthesis Adoption, amputation-related variables, prosthesis type, and participant demographics (race, gender, and age). SEM is composed of one or more measurement models, which measure the latent or composite variables, and a structural model which tests hypothetical relationships or associations (e.g., direct and indirect relationships) between latent constructs and covariates using path analysis [28, 29]. SEM development and analysis occurred in several stages: analysis of direct effects, analysis of moderation and mediation effects, and exploration of conditional indirect effects.

### Recruitment, sampling, and data collection

The data collection and analyses reported in this manuscript were conducted as part of a larger, parent study that aimed to develop and refine numerous measures for upper limb amputation rehabilitation. The full survey included questions about amputation history, level, etiology, prosthesis use, type and number of prostheses used, and payment source for the primary and secondary prosthesis. The survey included demographic questions related to sex, Veteran status, race, and ethnicity. It also included the Trinity Amputee Prosthetic Evaluation Scale (TAPES)-Upper prosthesis satisfaction measure [30], the Orthotics and Prosthetics User Survey (OPUS), Client Satisfaction with Device (CSD) scale [31], three new subscales from a revised OPUS CSD (Comfort, Appearance, Utility) [32] and additional items and measures related to psychosocial adjustment [33], activity performance [34, 35], patient experience [36], and residual limb health [37], and prosthesis affordability [38], all reported elsewhere.

The sample for this analysis was recruited from multiple sources: 1) participants from an earlier study,^4^ 2) Veterans who had received VA care between 1/1/16 and 6/1/19, 3) emails sent from the Amputee Coalition of America, and 4) recruitment letters sent from a private prosthetics service company. Recruitment took place between 10/29/2018 and 8/18/2020. Participants were included if they had ULA at the level of the wrist or above and were able to understand study requirements and hear well enough to comprehend questions administered over the telephone. All participants provided oral informed consent as approved by the Central VA Institutional Review Board. Data collection was conducted via telephone survey administered by trained survey center staff. Four hundred and two participants in the analytic sample were surveyed at baseline, and a smaller convenience sample of 50 persons were resurveyed within 2 weeks to obtain test-retest reliability data. While we did not conduct an a priori power analysis to determine the survey sample size, we believe that our sample size was sufficient, given that the number of participants was greater than the 30 participants recommended by others for IRT analysis.” [39].

### Measure development

The goal of this effort was to develop and refine a novel measure of prosthesis usability experience. Measure development occurred in several steps.

#### Item set development, cognitive and pilot testing

We developed an item set that addressed a variety of dimensions thought to be associated with experiences of prosthesis usability and extent of adopting (or using) a prosthesis. The item content was identified through informal discussions with several key informants (including a physical medicine and rehabilitation physician, an occupational therapist and a research assistant experienced in upper limb amputation), prior analyses of qualitative responses to open-ended questions in a national survey [15] and reviews of the literature. The major areas that were identified in this preliminary work were: preferences related to device cosmesis, overall desirability of available devices, lifestyle compatibility, adequate information about prosthetic choices, confidence regarding unintended consequences of prosthesis use, comfort of wearing the prosthesis, impact on the body and the acceptability of prosthesis fit with clothing, and burden/ease of prosthesis use. Items addressing these factors were generated by the investigative team or in some cases adopted from existing measures (S1 File).

Cognitive testing was then conducted to obtain feedback about the initial item set, identify questions that were unclear or misinterpreted, and to identify potential new items related to prosthesis acceptance. Eleven cognitive interviews were conducted by the study lead. Participants included 9 prosthesis users and 2 persons with ULA who were non-users including1 with bilateral amputation (63.6% male, mean age 54.4) (S1 Table). Cognitive-based interviewing involved both focused probing as well as asking respondents to “think out loud” as they answered questions, giving observers insights into respondent understanding and processing of each question. Respondents were asked about their comprehension, recall ability and strategy of information related to the question, decision processes, and response processes [40]. At the end of the interview participants were also asked to identify other factors that influenced their prosthesis use. Participants who did not use a prosthesis were asked to explain why they did not use a device, and those who used a device were asked to talk about prosthesis characteristics that they were most and/or least satisfied with. Feedback from the cognitive interviews was used to iteratively refine and add items (described in S2 File).

Prior to pilot testing, the item set was discussed with the survey team and minor changes to response categories language, and question wording were made. The item set was then pilot tested with a convenience sample of 20 participants (16 with unilateral and 4 with bilateral amputation, 15 prosthesis users, 5 non-users, 55% male and mean age 61.9) (S1 Table). Although the number of participants within key subgroups (laterality and prosthesis use) were not balanced, there were sufficient numbers in each subgroup to obtain feedback on the measure. Very minor changes were made based on interviewer and participant feedback. The refined item set was then field tested as part of the parent study of amputation-related measures. No pilot testing data was included in the analyses of field testing data.

#### Factor and Rasch analyses

Once the survey data was collected, we evaluated the structural validity of the new measures using factor and Rasch analyses. We followed the evaluation framework for key psychometric properties of patient reported outcome measures as defined by the National Institute of Health Patient-Reported Outcomes Measurement Information System (PROMIS) initiative [41]. Evaluation of structural validity utilized factor analyses and Rasch analyses [42, 43] and data from 402 prosthesis users. MPlus software [44] was used to conduct analyses.

Factor analyses were conducted to examine the underlying dimensions explaining the relationships between items. We conducted three separate item set evaluations for: 1) items related to cosmesis importance, 2) items related to prosthesis ease of use, 3) all other items. A separate exploratory factor analysis (EFA) was conducted for cosmesis importance items because of their unique response structure (only 3 response categories where neither higher nor lower are necessarily better). The six items related to prosthesis ease of use were considered a modification of the Utility Scale of the Prosthetic Evaluation Questionnaire [45], and thus were only evaluated in a separate confirmatory factor analysis (CFA). The remaining items were evaluated in a single EFA. This analysis resulted in six novel measures: Cosmesis Importance, Prosthesis Comfort, Prosthesis Trust, Appearance Acceptability, Prosthesis Desirability, and Prosthesis Ease of Use. We evaluated whether the ratio of 1st and 2nd eigenvalues was greater than 4 to assess the unidimensionality in EFA results, and used the comparative fit index (CFI), Tucker–Lewis Index (TLI), and root mean square error approximation (RMSEA) to examine CFA model fit.

Following these factor analyses we used Rasch partial credit modeling (PCM). Rasch analyses involves probabilistic modeling of latent traits where there is an intention to combine information across items to obtain a measure of some underlying variable (e.g. Extent of Prosthesis Adoption). Rasch analysis provides estimates of item difficulty (the manner in which each item behaves along the ability scale of the latent construct), as well as estimates of each person’s score on the measure. PCM is a type of Rasch model in which each item has a unique rating scale structure. We used PCM to evaluate monotonicity and mean square (MSNQ) fit statistics. We used two approaches to identify whether items had significantly different meanings for different groups (i.e. differential item functioning [DIF]) by age and gender within each factor (See S3 File) [46, 47]. We also examined reliability of scores using Cronbach alpha, and person and item reliability statistics to determine consistency of scores. We calculated intraclass correlation coefficients (ICC) (type 3,1) [48] and minimal detectable change (MDC) based on the 50-person sample that had test-retest data collected within a two-week period. We examined the distribution (floor and ceiling) of scores to determine whether the scales would have adequate ability to distinguish between persons at each end of the scale.

#### Validation of new measures

To examine concurrent and discriminant validity of the new measures resulting from factor and Rasch analyses, we estimated Pearson correlations among 1) these new measures, 2) two measures of prosthetic satisfaction–the 8-item OPUS CSD scale (using an 8-item CSD calibrated and employed in our prior study of persons with ULA) [24] and the satisfaction scale of the TAPES [30]–and 3) the 13-item PROMIS Upper Extremity amputation-specific measure (PROMIS UE-13 AMP), a measure of upper limb function with amputation sample-specific calibration [49]. Correlations above 0.30 were considered moderate, and those above 0.50 were considered strong [50, 51].

#### Latent constructs

A latent construct is one that cannot be directly measured or observed. We had two latent constructs for this analysis: Extent of Prosthesis Adoption and Prosthesis Usability Experience that were estimated using the indicator variables defined in Table 1. Indicators for the Prosthesis Usability Experience included the six novel measures developed by our team: Indicators for the Extent of Prosthesis Adoption latent construct were hours of prosthesis use, frequency of prosthesis use, and one- and two-handed task performance as measured by the Upper Extremity Functional Scale for Prosthesis Users (UEFS-P) One-handed Task scale and Two-handed Task scale, respectively [34]. Each latent construct was initially examined separately using EFA and CFA. For Prosthesis Usability Experience, we used maximum likelihood estimation (ML) with robust SE (standard error) method for both EFA and CFA since all indicators were continuous variables. Because two indicators of Extent of Prosthesis Adoption were categorical variables, we used weighted least squares mean and variance adjusted estimation for both EFA and CFA of this latent construct.

**Table 1:** Latent constructs and indicator variables explored in the SEM analysis.

Latent Construct^	Variable Name	Definition	Measure Details#	Interpretation
Extent of prosthesis adoption	Hours of use	The number of hours the respondent wears the prosthesis in a typical day.	Response categories: < 2 hours, 2 to < 4 hours, 4 to < 8 hours, 8 to <12 hours, and ≥12 hours	More hours indicate greater adoption
	Days of use	The average frequency that the respondent wears the prosthesis in a typical month.	Response categories: daily, 2 to 3 times per week, once a week, a few times a month, or once a month	Greater frequency indicates greater adoption
	UEFS-P One handed Task scale	The performance of one-handed activities of daily living, such as drinking from a cup, with the prosthesis.	9-items; Response categories: “Do not do activity” and “Do activity”	Higher scores indicate greater adoption
	UEFS-P Two-handed Task scale	The perceived ease of performing two-handed activities of daily living, such as carrying a laundry basket, with the assistance of the prosthesis.	20-items; Response categories: “Do not do activity,” “Attempted with some difficulty,” and “Very easy”	Higher scores indicate greater adoption
Prosthesis usability experience**	Cosmesis Importance*	The importance the respondent places on liking their appearance when wearing the prosthesis, having a prosthesis that allows wearing jewelry or a watch, looks good with clothing, and has a natural-looking hand with fingernails.	items total 1-item; Response scale: Strongly disagree to Strongly agree 3 items; Response scale Not at all important to Very important	Higher scores indicate that the appearance of the prosthesis has high significance to the participant
	Prosthesis Comfort	The extent of pain or discomfort caused by wearing the prosthesis.	4-items; Response scale: Strongly disagree to Strongly agree	Higher scores indicate that wearing the prosthesis does not cause bodily pain
	Prosthesis Trust	The likelihood that the respondent would not avoid wearing or using the prosthesis when caring for a baby and extent to which they are unafraid of hurting or scaring someone with the prosthesis.	3-items; Response scale: Strongly disagree to Strongly agree	Higher scores indicate that respondents are less likely to avoid engaging their prosthesis during specific tasks because of concerns about inadvertent consequences.
	Appearance Acceptability	The frequency that the respondent did not avoid wearing a prosthesis because of the way that it fits with or under clothes.	3-items; Response scale: Always/Regularly to Never	Higher scores indicate respondents find the fit of the prosthesis under clothes more acceptable
	Prosthesis Desirability	The respondent’s opinions on the suitability and desirability of prostheses, adequacy of information about current prostheses, ability to get desired prostheses, consistent functionality of their prosthesis, and satisfaction with wrist function.	6-items; Response scale: Strongly disagree to Agree	Higher scores indicate that respondents were more satisfied with currently available prostheses.
	Prosthesis Ease of Use	The frequency that the respondent felt off-balance when wearing the prosthesis, the frequency that the prosthesis got in the way of activities, and the amount of physical and mental energy it took to use the prosthesis.	4-items total. 2 items with response scale of Extreme amount to None, 2 items with response scale of All the time to Not at all.	Higher scores indicate greater ease of prosthesis use

^ Latent constructs are unobserved constructs that can be inferred from measured indicator variables.

^#^ Final response categories after correcting for monotonicity by collapsing categories are detailed in S1 File.

* The Cosmesis importance scale was dropped from the SEM after model 1.

** Usability Experience measures are worded such that they measure the inverse of each construct. These scales are reverse coded so that higher scores indicate better outcomes.

#### Additional covariates

Five additional covariates related to participant demographics, amputation characteristics, and prosthesis characteristics were included in our analyses. Age was considered as a continuous variable. Gender was categorized as male or female. Race was categorized as white, black, other (including multiple races), and unknown. Amputation level/laterality was considered as a single variable in analyses, such that participants were categorized as having bilateral amputation, regardless of the level of amputation of either limb, following the precedent of earlier studies [37]. For participants with unilateral limb loss, amputation level was categorized as TR—which includes amputation at the below elbow or wrist disarticulation levels, TH—which includes amputation at the above elbow and elbow disarticulation levels, and SH—which includes shoulder disarticulation and forequarter amputation. The prosthesis used most often (primary prosthesis) type was classified into four categories based on the type of control scheme and type of terminal device: body-powered (BP), myoelectric single degree-of-freedom terminal device (myo single-DOF), myoelectric multiple degree-of-freedom terminal device (myo multi-DOF), and cosmetic. Hybrid devices were grouped with the appropriate myoelectric category based on the type of terminal device. For persons with bilateral ULA the prosthesis used on the dominant side was considered the primary prosthesis.

#### Structural equation modeling

The purpose of SEM is to examine the structural relationship between measured variables and latent constructs. Our SEM contained two measurement models which were used to estimate the latent constructs of 1) Extent of Prosthesis Adoption and 2) Prosthesis Usability Experience. We calculated the descriptive statistics for variables used in the SEM model (such as mean, standard deviation, range of scores, and a frequency table for categorical variables). We examined the normality of each continuous variable by generating histogram plots and calculating skewness and kurtosis, with values outside the range of -2 and 2 considered to be in violation of normality assumptions. To better understand the content of each latent construct and its variability we also examined the distribution of latent constructs of Extent of Prosthesis Adoption and Prosthesis Usability Experience as well as each individual indicator variable by amputation level/laterality and prosthesis type.

**Fig 2:**
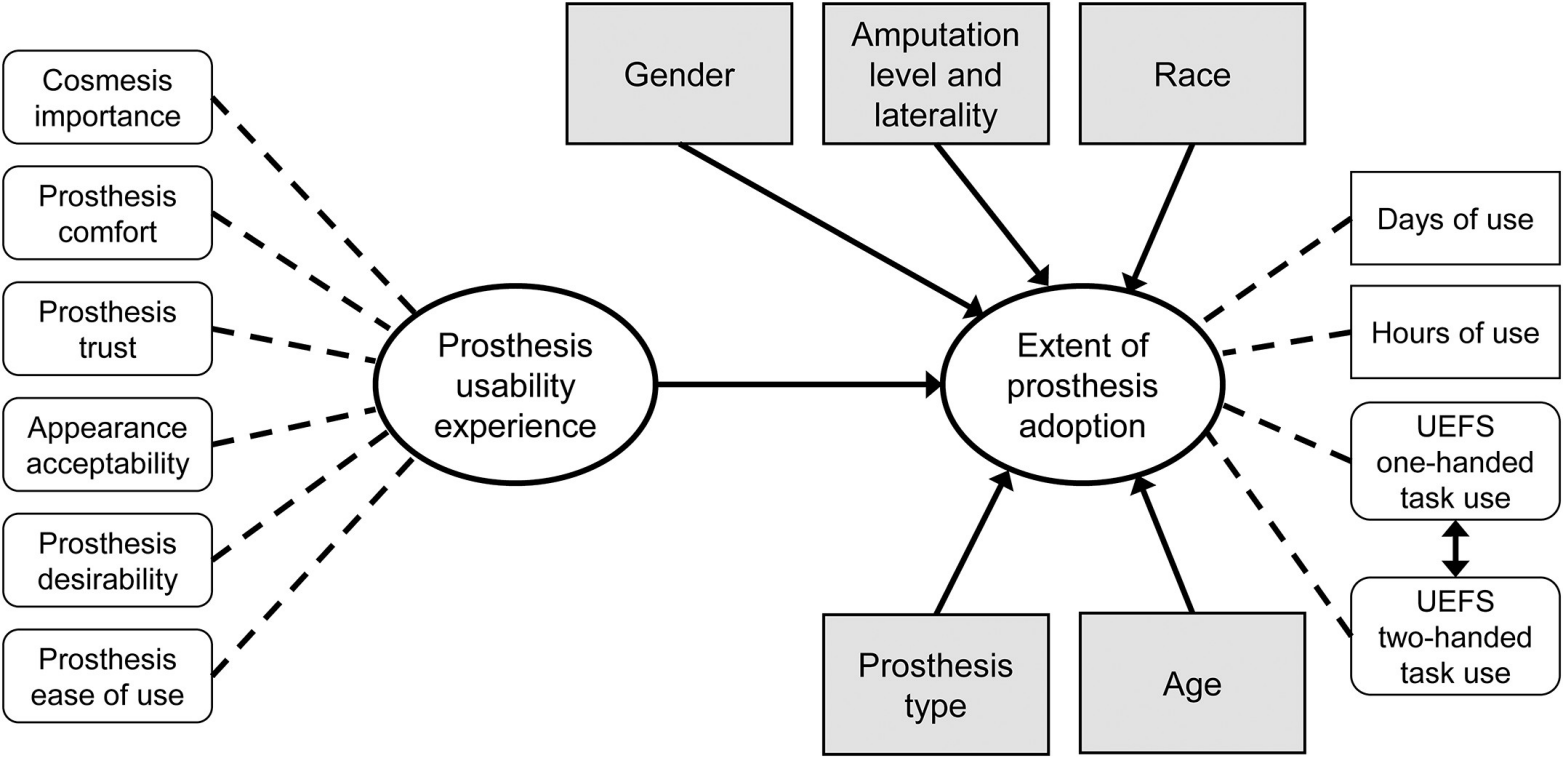
Path diagram for the basic conceptual model of Extent of Prosthesis Adoption. Ovals represent latent constructs modeled through CFA. Rounded white rectangles are indicator measures comprised of >1 self-report items while white rectangles with corners are single item indicator variables. Grey rectangles are covariates. Solid lines represent regression coefficients for direct effects, dotted lines indicate factor loadings of indicator variables onto latent constructs. The double-headed arrow represents a correlation effect.

The basic conceptual model guiding our analysis is shown in the path diagram in Fig 2. The theoretical model contained the two latent constructs (shown in ovals), as well as the covariates related to amputation, demographic factors (gender, age, race), and prosthesis type. The indicator variables in the measurement models for each latent construct are shown as dashed lines. In the theoretical model, there are a series of regression pathways, each with its own regression coefficient, called a path coefficient (shown with a solid, single-headed arrow), which describes the strength of the relationship between the variables (or constructs) in that pathway.

We developed the SEM model to fit the data and tested it in several stages, exploring multiple models to build the path analysis. Our first SEM tested the direct effects of the latent construct of Prosthesis Usability Experience and all covariates on the latent construct of Extent of Prosthesis Adoption.

Our subsequent model tested moderating and mediating effects between the two latent constructs and the subset of covariates that had statistically significant direct effects in the initial model. Moderating variables, sometimes called interaction effects, are those that influence the strength and direction of a relationship between two variables. In contrast, mediating variables, sometimes called indirect, endogenous, or distal causal influences, explain the process through which two variables are related, by intervening between the two variables in a causal chain. Finally, we tested moderated mediation effects, also known as conditional indirect effects, which occur when the effect of one variable on another (outcome) variable via a mediating variable differs depending on the level of a moderator variable. SEM analyses were generated from Mplus version 7.4 [52]. All other analyses were generated using SAS software version 9.4.

## Results

### Sample

The analytic sample included 402 prosthesis users who had complete data (Table 2). On average, participants were 61.7 +/- 14.4 years of age and had received their amputation 30.2 +/- 20.2 years prior, with the majority reporting amputation at the transradial level (66.2%). The sample predominantly consisted of white (84.3%), Veteran (77.1%) men (80.4%) who had acquired amputation (90.8%). Most participants used body-powered (68.2%) prostheses and had received some degree of prosthesis training in the past (64.2%).

**Table 2:** Demographic and prosthetic characteristics of the analytic sample for structural equation modeling.

Variable	Analytic Sample (N = 402)
	**Mn (sd)**
**Age (N = 402)**	61.7 (14.4)
Years since amputation* (N = 353)	30.2 (20.2)
**Status** *	**N (%)**
Veteran	309 (77.1)
Military	0 (0.0)
Civilian	92 (22.9)
**Gender**	
Female	79 (19.7)
Male	323 (80.4)
**Race**	
White	339 (84.3)
Black	32 (8.0)
Other	14 (3.5)
Unknown	17 (4.2)
**Amputation level**	
Shoulder	24 (6.0)
Transhumeral	80 (19.9)
Transradial	266 (66.2)
Bilateral	32 (8.0)
**Congenital**	
Yes	37 (9.2)
No (acquired)	365 (90.8)
**Prosthesis training**	
Yes	258 (64.2)
No	144 (35.8)
**Primary prosthesis type**	
Body-powered	274 (68.2)
Myoelectric Single-DOF	60 (14.9)
Myoelectric Multi-DOF	45 (11.2)
Cosmetic	23 (5.7)

* 50 participants missing data for time since amputation; 1 missing data for military status

### Measurement development

At the completion of EFA and CFA (with removal of items that did not load well or had poor residual correlations), there were 5 scales with good evidence of unidimensionality. Detailed results of factor analyses can be found in S3 File.

These scales were labeled: Prosthesis Comfort (4 items), Prosthesis Trust (3 items), Appearance Acceptability (3 items), Prosthesis Desirability (6 items) and Cosmesis importance (5 items). All scales were designed so that higher scores indicated more of the construct measured by the scale. Thus, higher scores of Cosmesis Importance (vs. lower scores) indicate that appearance of the prosthesis is more important. Higher scores of the Prosthesis Desirability scale indicate that respondents were more satisfied with currently available prostheses. Higher scores of the Prosthesis Trust scale indicates that respondents are less likely to avoid engaging their prosthesis during specific tasks because of concerns about inadvertent consequences. Higher scores of Prosthesis Comfort indicate that wearing the prosthesis does not cause bodily pain. Finally, higher scores of Appearance Acceptability scale indicate that respondents find the fit of the prosthesis under clothes more acceptable (Table 1).

Rasch analyses led to refinement of the response categories and measure content and after refinements and adjustment for severe DIF (S2 and S3 Tables). We standardized the Rasch summary score for each measure (as calculated on a logit scale) into a T-score matrix for the sample.

Rasch person reliability was 0.61 for Cosmesis Importance (0.78 among prosthesis users only), 0.80 for Prosthesis Comfort, 0.72 for Prosthesis Trust, 0.61 for Appearance Acceptability, 0.78 for Prosthesis Desirability and 0.61 for the Ease of Use scale (S4 Table). Cronbach’s alpha was above 0.80 for the Prosthesis Comfort and Prosthesis Desirability scales, between 0.70 and 0.80 for the Cosmesis Importance scale (prosthesis users only), 0.68 for Ease of Use, but below 0.60 for the Appearance Acceptability and Prosthesis Trust scales. Test-retest reliability ICCs(3,1) were 0.63, 0.61, 0.65, 0.48, 0.52, 0.56 for the Cosmesis Importance, Prosthesis Desirability, Prosthesis Trust, Prosthesis Comfort and Acceptability of Fit, and Prosthesis Ease of Use scales, respectively. Confidence intervals and MDC values are also shown in S4 Table. Less than 15% of the sample were at the floor (0.4%-6.9%) of possible scores. However, a large ceiling effect was observed in the appearance scale with 41.3% of participants selecting only the best response category (‘never’). Final copies of usability measures used in our analyses are provided in S4 File, and crosswalks to convert summed scores to Rasch-based scoring are provided in S5 File.

#### Validation of new measures

Pearson correlations, examining concurrent validity between our new measures and other measures expected to be related are shown in S5 Table. Weak correlations were observed between Cosmesis Importance, Prosthesis Trust and the two prosthesis satisfaction measures (TAPES and OPUS CSD-8); and between all measures except Ease of Use and upper limb function (PROMIS UE-13 AMP). Moderate correlations were observed between Prosthesis Comfort, Appearance Acceptability and Prosthesis Ease of Use and at least one of the prosthesis satisfaction measures. Ease of Use was also moderately associated with upper limb function.

### Development of latent constructs

Initial EFA of Extent of Prosthesis Adoption indicators showed that 66% of the variance was explained by the first factor, suggesting that the items in the factor were unidimensional. Subsequent CFA had poor fit indices (CFI = 0.944, TLI = 0.832, RMSEA = 0.260). There was also high residual correlation between the UEFS-P One-handed and Two-handed Task scales. To address this, we added a correlation effect between the One-handed and Two-handed UEFS-P measures to the subsequent CFA, resulting in a better fit (CFI = 0.999, TLI = 0.993, RMSEA = 0.052).

The EFA conducted on the six Prosthesis Usability Experience indicator measures showed that five measures (all but Cosmesis Importance) loaded adequately (>0.3) on the latent construct of Prosthesis Usability Experience. After removing the Cosmesis Importance scale, EFA showed that 46% of the variance was explained by the first factor. In the CFA, CFI was 0.982, TLI was 0.965, and RMSEA was 0.052. Factor loadings of indicator variable scores are presented as standardized scores (i.e., z-scores) for the measures on the latent constructs that were used in subsequent structural equation models.

### SEM direct effects

**Fig 3:**
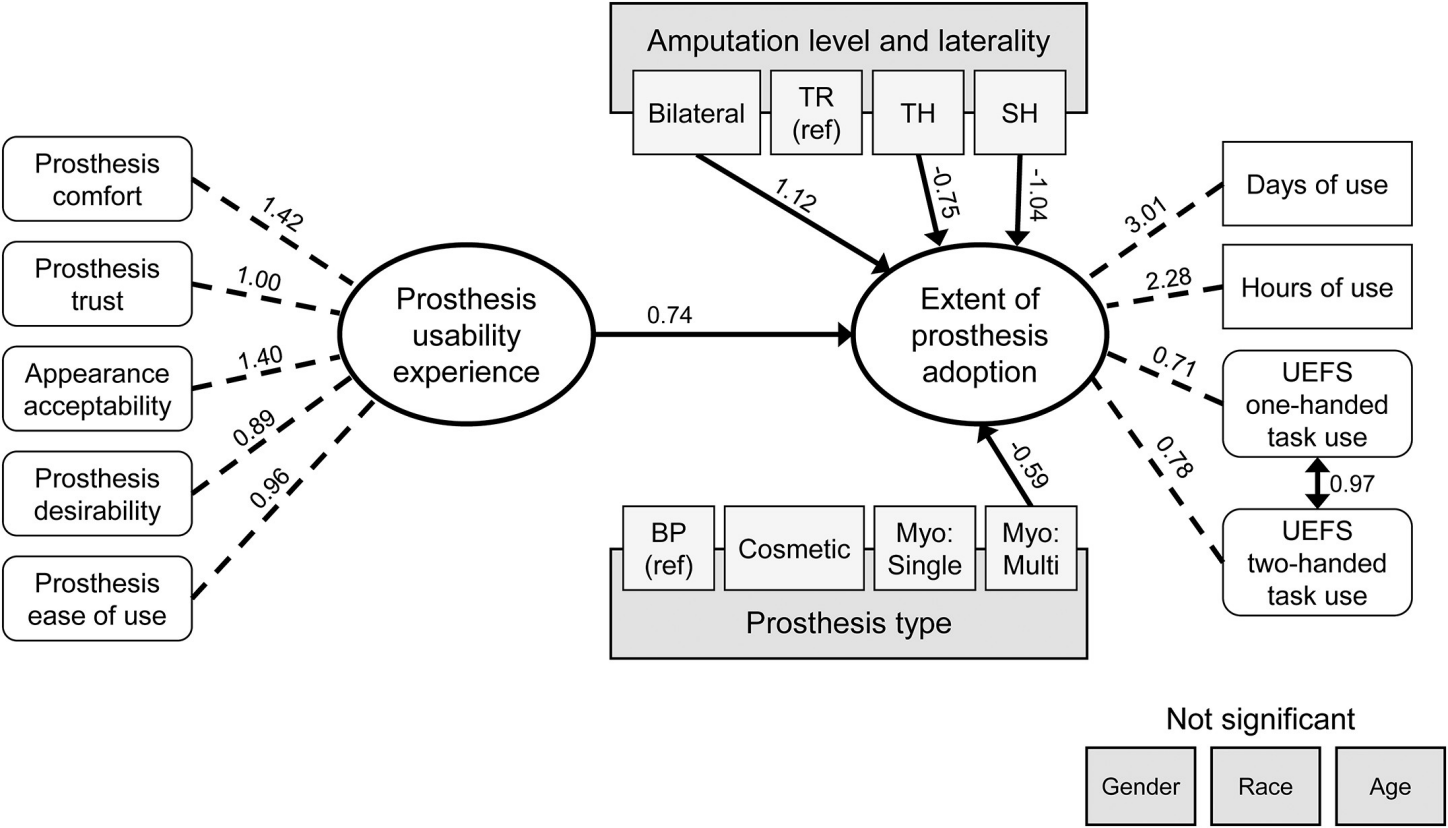
SEM initial model of Extent of Prosthesis Adoption showing significant direct effects only. Ovals represent latent constructs modeled through CFA. Rounded white rectangles are indicator measures comprised of >1 self-report items while white rectangles with corners are single item indicator variables. Darker grey rectangles are covariates. Note that the sub-categories of amputation level, laterality, and prosthesis type have been included as lighter grey rectangles within their respective covariate. Solid lines represent regression coefficients for direct effects, dotted lines indicate factor loadings of indicator variables onto latent constructs. The double-headed arrow represents a correlation effect. Note that gender, race, and age did not have significant direct effects on Extent of Prosthesis Adoption.

All continuous variables included in the SEM met the criteria of normality (skewness and kurtosis values within -2 and 2 range). The initial SEM (Fig 3) examined only the effect of Prosthesis Usability Experience on Extent of Prosthesis Adoption and the effects of covariates (i.e. amputation level/laterality, prosthesis type, age, race, gender) on Extent of Prosthesis Adoption. In this model, 46% of the variance in the data was explained. We found a direct effect of Prosthesis Usability Experience on the Extent of Prosthesis Adoption (β = 0.74, p<0.001), where higher Prosthesis Usability Experience scores were associated with higher Extent of Prosthesis Adoption scores. There were also direct effects of amputation level and laterality on Extent of Prosthesis Adoption. Specifically, those with TH and SH level amputation (β = -0.75, p<0.001; β = -1.04, p <0.001, respectively) had lower Extent of Prosthesis Adoption scores and those with bilateral amputations (β = 1.12, p<0.001) had higher Extent of Prosthesis Adoptions scores compared to the reference group of TR ULA. Finally, there was a direct effect of prosthesis type, wherein users of myoelectric multi-DOF devices (β = -0.59, p = 0.007) have lower Extent of Prosthesis Adoption scores compared to the reference group of BP device users. No statistically significant effects were observed for race, age, or gender.

### SEM moderation and mediation effects

**Fig 4:**
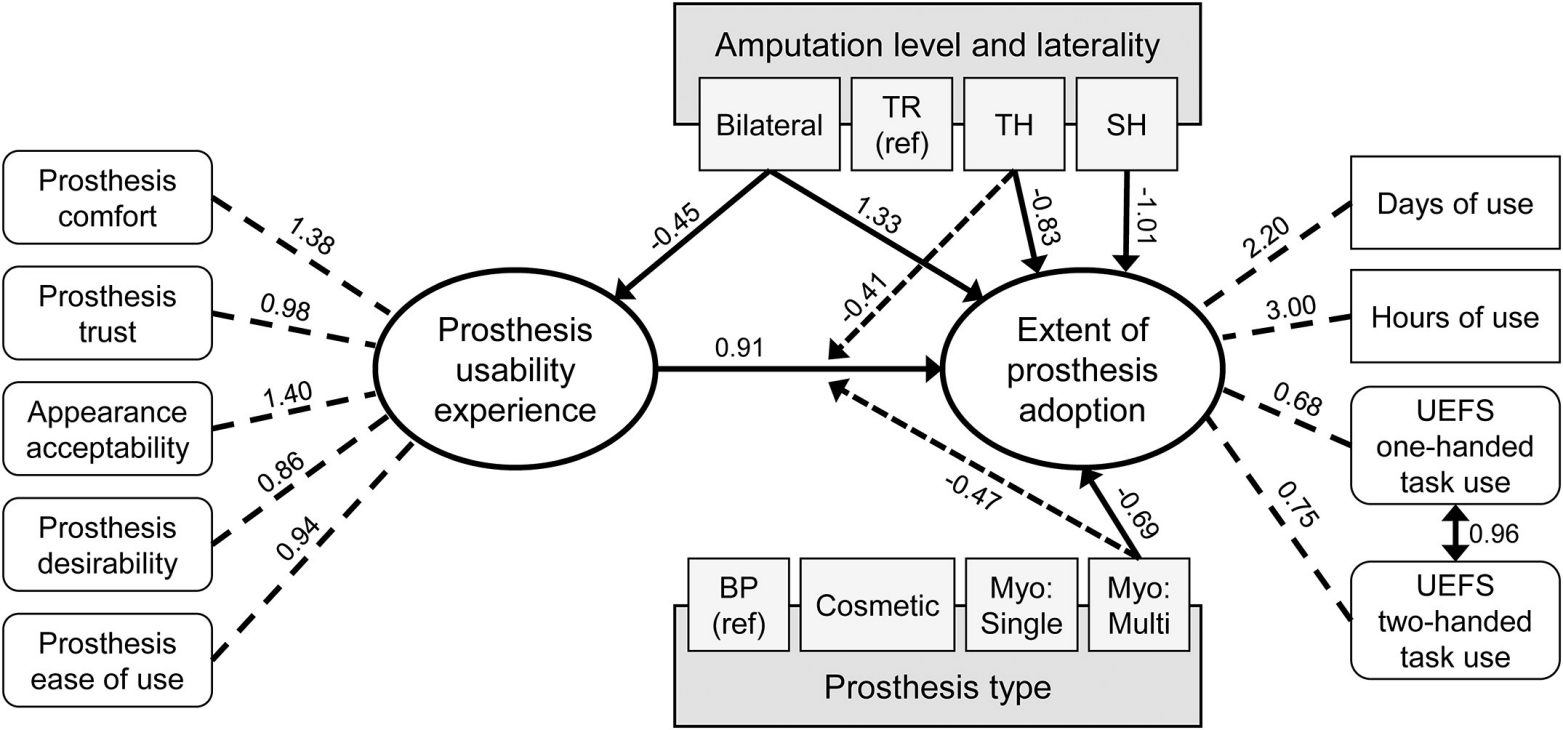
Final SEM model of Extent of Prosthesis Adoption showing all significant direct, moderating, and mediating effects. Ovals represent latent constructs modeled through CFA. Rounded white rectangles are indicator measures comprised of >1 self-report items while white rectangles with corners are single item indicator variables. Darker grey rectangles are covariates. Note that sub-categories of amputation level, laterality, and prosthesis type have been included as lighter grey rectangles within their respective covariate. Solid arrows represent regression coefficients for direct effects, and dashed arrows represent regression coefficients for moderating effects. Dotted lines indicate factor loadings of indicator variables onto latent constructs. The double-headed arrow represents a correlation effect.

The final SEM included moderating and mediating relationships among variables (Fig 4), in addition to the direct effects uncovered in the initial model. The final model explained 51.5% of variance in Extent of Prosthesis Adoption scores. All direct effects found in the initial model were also observed in the final SEM, but the values of the regression coefficients often changed. Results of the final SEM demonstrated that overall, increases in Prosthesis Usability Experience scores resulted in higher Extent of Prosthesis Adoption scores (β = 0.91, p<0.001). As before, there was a significant direct effect of amputation level on Extent of Prosthesis Adoption, where those with TH and SH level amputation had lower Extent of Prosthesis Adoption scores compared to TR (TH: β = -0.83, p<0.001; SH: β = -1.01, p = 0.001), while those with bilateral amputation had higher Extent of Prosthesis Adoption scores (β = 1.33, p = 0.007). There was also a significant direct effect by prosthesis type: those using myoelectric multi-DOF prosthesis had lower Extent of Prosthesis Adoption scores than those using body-powered prostheses (β = -0.69, p = 0.003).

Prosthesis type and amputation level both significantly moderated the overall effect of Prosthesis Usability Experience scores on Extent of Prosthesis Adoption scores. There were no statistically significant findings for myoelectric single-DOF devices, nor for cosmetic devices as shown in Figs 3 and 4. Those with myo multi-DOF prostheses (compared to BP) had a reduction (β = -0.47, p = 0.050) of the direct effect of Prosthesis Usability Experience scores on Extent of Prosthesis Adoption scores. Thus, while every 1-point increase in Prosthesis Usability Experience corresponded to a 0.91-point increase in Extent of Prosthesis Adoption for BP users, users of multi-DOF myoelectric prostheses only had a 0.44-point increase in Extent of Prosthesis Adoption with every 1-point increase in Prosthesis Usability Experience. Those with TH amputation (compared to TR) also had a reduction (β = -0.41, p = 0.048) in the direct effect of Prosthesis Usability Experience scores on Extent of Prosthesis Adoption, compared to those with TR amputation. In other words, those with TH amputation had a 0.60-point increase in Extent of Prosthesis Adoption for every 1-point increase in Prosthesis Usability Experience.

Only one significant mediation effect was observed. Those with bilateral ULA (compared to those with unilateral TR ULA) had lower Prosthesis Usability Experience scores (β = -0.45, p = 0.046). Thus, the relationship between amputation laterality and the Extent of Prosthesis Adoption was mediated by the Prosthesis Usability Experience scores. For those with bilateral ULA, the strength of the relationship between Prosthesis Usability Experience and Extent of Prosthesis Adoption was therefore reduced to -0.41 (0.91 multiplied by -0.45) (p = 0.054).

### SEM exploration of conditional indirect effects

There were no statistically significant conditional indirect effects (i.e., moderated mediation effects). We examined the standard error of each coefficient that comprised the conditional indirect effect from bilateral amputation (vs TR) mediated through Prosthesis Usability Experience and found there was large variation in the moderated effect from amputation level (Bilateral vs TR) on the association between Prosthesis Usability Experience scores and Extent of Prosthesis Adoption scores, which likely resulted in the insignificant conditional indirect effect.

The distributions of Extent of Prosthesis Adoption and Prosthesis Usability Experience latent construct scores as well as the distributions of the indicator variables by amputation level/laterality and prosthesis type are shown in Tables 3 and 4. Briefly, those with more proximal level amputation (TH and SH) had lower Prosthesis Usability Experience and Extent of Prosthesis Adoption scores as compared to those with TR amputation. Those with bilateral amputation had the lowest Prosthesis Usability Experience but the highest Extent of Adoption scores. Prosthesis Usability Experience scores were highest for myo single DOF users followed by cosmetic and body-powered users and lowest for myo multi DOF users. Extent of Adoption scores were also highest for myo single DOF users, followed by body-powered and cosmetic users, and lowest for myo multi-DOF users.

**Table 3:** Distribution of Extent of Prosthesis Adoption and prosthesis usability experience latent construct scores and indicator variable scores by amputation level/laterality.

	Amputation Level				
	SH (N = 24)	TH (N = 80)	TR (N = 266)	Bilateral* (n = 32)	All (N = 402)
	Mn (Sd)	Mn (Sd)	Mn (Sd)	Mn (Sd)	Mn (Sd)
**Latent Constructs** ^†^					
Prosthesis Usability Experience	−0.2 (0.8)	−0.1 (0.8)	0.1 (0.9)	−0.3 (0.7)	−0.2 (0.8)
Extent of Prosthesis Adoption	−0.6 (0.7)	−0.3 (0.8)	0.1 (0.9)	0.6 (0.8)	−0.6 (0.7)
**Prosthesis Usability Indicators** ^‡^					
Prosthesis Comfort	50.2 (12.1)	50.8 (10.4)	52.3 (9.8)	47.7 (9.2)	50.2 (12.1)
Prosthesis Trust	48.2 (10.2)	48 (9.2)	47.5 (9.6)	46.5 (6.6)	48.2 (10.2)
Appearance Acceptability	49.2 (9.6)	50.9 (9.7)	51.2 (9.4)	49.7 (9.9)	49.2 (9.6)
Prosthesis Desirability	52.3 (7.5)	53.2 (8.6)	54.1 (9)	52.4 (12)	52.3 (7.5)
Ease of Use	46.4 (8.7)	48.1 (9.5)	51.2 (10.3)	47 (9.6)	46.4 (8.7)
**Extent of Prosthesis Adoption Indicators** ^‡^					
UEFS-P One-handed Tasks	42.6 (7)	44.9 (7)	50.5 (8.7)	66.9 (10.6)	42.6 (7)
UEFS-P Two-handed Tasks	44.1 (9.4)	46.1 (10.2)	52.2 (9)	51.1 (10.9)	44.1 (9.4)
	**N (%)**	**N (%)**	**N (%)**	**N (%)**	**N (%)**
**Days of prosthesis use**					
Daily	12 (50.0)	47 (58.8)	210 (79.0)	30 (93.8)	299 (74.4)
2 to 3 times per week	10 (31.7)	24 (30.0)	39 (14.7)	2 (6.3)	75 (18.7)
Once a week	1 (4.2)	5 (6.3)	9 (3.4)	0 (0.0)	15 (3.7)
A few times a month	0 (0.0)	3 (3.8)	7 (2.6)	0 (0.0)	10 (2.5)
Once a month	1 (4.2)	1 (1.3)	1 (0.4)	0 (0.0)	3 (0.8)
Once every few months	0 (0.0)	0 (0.0)	0 (0.0)	0 (0.0)	0 (0.0)
1 to 3 times per year	0 (0.0)	0 (0.0)	0 (0.0)	0 (0.0)	0 (0.0)
**Hours/day prosthesis use**					
<2 hours	8 (33.3)	14 (17.5)	30 (11.3)	1 (3.1)	53 (13.2)
2 to 4 hours	5 (20.8)	12 (15.0)	24 (9.0)	3 (9.4)	44 (11.0)
4 to 8 hours	5 (20.8)	16 (20.0)	41 (15.4)	4 (12.5)	66 (16.4)
8 to 12 hours	3 (12.5)	23 (28.8)	69 (25.9)	5 (15.6)	100 (24.9)
12 hours or more	3 (12.5)	15 (18.8)	102 (38.4)	19 (59.4)	19 (59.4)

* Bilateral amputation includes persons with any level of upper limb amputation on both sides

^†^ Prosthesis usability experience scores are presented as non-standardized scores that were used in the models

^‡^ The indicators of the two latent constructs are presented at converted t-scores, centered at 50 with a SD of 10. Possible scores range from 0–100, higher scores indicated more of that construct

**Table 4:** Prosthesis usability experience and Extent of Prosthesis Adoption: Latent construct and indicator variable scores by prosthesis type (N = 402).

	Primary Prosthesis Type				
	Body-powered (N = 274)	Myo: Single DOF (N = 60)	Myo: Multi DOF (N = 45)	Cosmetic (N = 23)	All (N = 402)
	Mn (Sd)	Mn (Sd)	Mn (Sd)	Mn (Sd)	Mn (Sd)
**Latent Constructs** *					
Prosthesis Usability Experience	0.0 (0.8)	0.2 (1.0)	−0.1 (0.9)	0.1 (0.7)	0.0 (0.8)
Extent of Prosthesis Adoption	0.0 (0.9)	0.1 (0.8)	−0.2 (0.7)	−0.1 (0.6)	0.0 (0.9)
**Prosthesis Usability Experience** ^†^					
Prosthesis Comfort	50.6 (9.8)	54.1 (10)	53.7 (11.7)	50.7 (9)	50.6 (9.8)
Prosthesis Trust	46.7 (9.2)	49.1 (10.5)	48.4 (8.8)	53.0 (6.2)	46.7 (9.2)
Appearance Acceptability	51.5 (9.5)	51.1 (8.5)	48.3 (9.8)	49.6 (10)	51.5 (9.5)
Prosthesis Desirability	53.9 (8.6)	54.4 (10.8)	51.8 (9.8)	51.8 (7.9)	53.9 (8.6)
Ease of Use	49.9 (10.3)	51.1 (10.9)	47.9 (8.5)	52.3 (8.7)	49.9 (10.3)
**Extent of Prosthesis Adoption Indicators** ^†^					
UEFS-P One-handed Tasks	50.3 (10.3)	53.2 (9.8)	49.1 (9.3)	44.5 (7.6)	50.3 (10.3)
UEFS-P Two-handed Tasks	50.8 (9.9)	52.8 (9.7)	48.8 (7.8)	43.3 (10.1)	50.8 (9.9)
	**N (%)**	**N (%)**	**N (%)**	**N (%)**	**N (%)**
**Days of Prosthesis Use**					
Daily	203 (74.1)	46 (76.7)	32 (71.1)	18 (78.3)	299 (74.4)
2 to 3 times per week	48 (17.5)	12 (20.0)	10 (22.2)	5 (21.7)	75 (18.7)
Once a week	12 (4.4)	1 (1.7)	2 (4.4)	0 (0.0)	15 (3.7)
A few times a month	8 (2.9)	1 (1.7)	1 (2.2)	0 (0.0)	10 (2.5)
Once a month	3 (1.1)	0 (0.0)	0 (0.0)	0 (0.0)	3 (0.8)
Once every few months	0 (0.0)	0 (0.0)	0 (0.0)	0 (0.0)	0 (0.0)
1 to 3 times per year	0 (0.0)	0 (0.0)	0 (0.0)	0 (0.0)	0 (0.0)
**Hours/day prosthesis use**					
<2 hours	39 (14.2)	7 (11.7)	5 (11.1)	2 (8.7)	53 (13.2)
2 to 4 hours	32 (11.7)	5 (8.3)	7 (15.6)	0 (0.0)	44 (11.0)
4 to 8 hours	35 (12.8)	11 (18.3)	15 (33.3)	5 (21.7)	66 (16.4)
8 to 12 hours	63 (23.0)	16 (26.7)	11 (24.4)	10 (43.5)	100 (24.9)
12 hours or more	105 (38.3)	21 (35.0)	7 (15.6)	6 (26.1)	139 (34.6)

* Prosthesis usability experience scores are presented as non-standardized scores that were used in the models.

^†^ Prosthesis usability experience scores are presented as non-standardized scores that were used in the models

^‡^ The indicators of the two latent constructs are presented at converted t-scores, centered at 50 with a SD of 10. Possible scores range from 0–100, higher scores indicated more of that construct.

## Discussion

The study introduces new, psychometrically valid measures of Prosthesis Usability Experience and used SEMs to examine the relationships between the latent constructs of Prosthesis Usability Experience and Extent of Prosthesis Adoption. The new measures of Prosthesis Usability Experience (Cosmesis Importance, Prosthesis Comfort, Prosthesis Trust, Appearance Acceptability, Prosthesis Desirability and Ease of Use) are a unique contribution to the field. Analyses of Pearson correlations supported the concurrent and discriminant validity of these new measures, demonstrating they have unique content not captured in existing measures of prosthesis satisfaction or upper limb function. Furthermore, they have acceptable reliability as determined in our final SEM model (Coefficient Omega reliability = 0.71). Most commonly used existing validated measures for persons with ULA pertain primarily to function, prosthesis satisfaction, or psychosocial adjustment and were not specifically developed to assess usability factors associated with prosthesis adoption [31, 53–56]. Thus the novel measures of Prosthesis Usability Experience introduced in this manuscript fill a gap in the field and could help both clinicians and researchers working in the field of ULA rehabilitation better understand patient experiences.

Findings supported our hypothesis that Prosthesis Usability Experience is positively associated with Extent of Prosthesis Adoption and identified other key relationships. The direct effect of amputation level on Extent of Prosthesis Adoption that we observed aligns with prior research showing that those with more proximal limb loss use their prostheses for fewer hours per day, and are more likely to reject their prosthesis [2, 7, 57]. An examination of the indicator variable scores show that individuals with TH and SH amputation had slightly higher prosthesis trust scores but lower prosthesis comfort, appearance acceptability, prosthesis desirability, and ease of use scores as compared to TR. Although our study was not designed to explain why TH amputation level, but not SH level, moderates the effect of Prosthesis Usability Experience on Extent of Prosthesis Adoption, we believe that this effect might be attributable to unmeasured variables that impact persons with TH amputation level, but were not well captured in our set of indicator variables. For example, we did not have an indicator measure assessing fatigue associated with prosthesis use, a common problem amongst those with shorter residual limbs due to impaired muscle strength [58].

Interestingly, our findings demonstrate that while those with bilateral ULA have lower Prosthesis Usability Experience scores than those with unilateral TR ULA, they still have higher Extent of Prosthesis Adoption scores. This could be interpreted to mean that while those with bilateral ULA find that their devices are less usable, desirable, and/or comfortable, their greater Extent of Prosthesis Adoption is driven by necessity of the device. The finding of greater adoption amongst those with bilateral ULA is consistent with prior literature demonstrating that people with bilateral ULA use their prosthesis more than those with unilateral ULA [15, 57, 59]. Persons with unilateral ULA can use their intact contralateral hand/arm to complete tasks and forgo wearing and using a prosthesis in certain situations [25]. However those with bilateral ULA must wear a prosthesis to perform many tasks, given that they do not have an intact hand/arm for grasping.

The finding that myo multi-DOF devices had a negative direct effect on Extent of Prosthesis Adoption and also lessened the effect of Prosthesis Usability Experience on Extent of Adoption was somewhat surprising. Myo multi-DOF devices are perhaps the most anthropomorphic of prosthetic terminal device types, given their appearance and movement patterns that are more naturalistic than single DOF hands or hooks. Interestingly, we found that myo multi-DOF users rated Appearance Acceptability lower than did BP users, but they rated Prosthesis Trust higher. This suggests that myo multi-DOF users may have high standards of device appearance and are sensitive to social perceptions. Thus, other factors beyond prosthesis usability might motivate them to have greater prosthesis adoption, such as greater social acceptability when wearing a device that looks high tech and anthropomorphic. The higher scores of the Prosthesis Trust scale seen in this group suggests that the softer feel, non-metallic materials and anthropomorphic shape of the myoelectric hand may help reduce concerns about handling a baby or surprising/scaring others because of the appearance of a hook on BP terminal devices.

A strength of our study was the novel and innovative analytic approach. To date, there has been limited use of SEMs in studies of rehabilitation interventions and outcomes [60–65], and no prior studies using SEM in the field of upper limb prosthetics. A benefit of SEM is that it enables estimation of the relationships of multiple variables of interest within a single statistical structure, allowing an examination of the paths or mechanisms through which effects occur.

Whereas previous studies have utilized single measures of prosthesis rejection (typically a binary outcome) [1, 3, 5, 7, 59, 66], or simpler composite measures of prosthesis acceptance/rejection [66], we employed a novel, latent construct approach to measuring the Extent of Prosthesis Adoption–estimating it using a variety of indicator variables (e.g., hours of prosthesis use per day, frequency of prosthesis use, and engagement of the prosthesis in one- and two-handed task performance). Reliability of this measurement from the final SEM model was good (0.73).

We used a similar latent construct approach to estimate Prosthesis Usability Experience by including indicators of prosthesis comfort, trust, appearance acceptability, prosthesis desirability, and ease of use. Using a latent construct approach allowed us to overcome some of the challenges of using composite measures that are not unidimensional [67], and the difficulties of using single indicators of prosthesis use which contain limited information. We believe that our latent construct approach to quantifying prosthesis usability experience is a strength that enhances internal validity of this research and could be replicated in future studies.

### Limitations and future directions

There are several limitations that should be considered when interpretating our work. Our large, convenience sample was comprised largely of white, older male, US Veterans with unilateral TR amputation. Therefore, the generalizability of our findings may be limited to those with demographic characteristics within the U.S. similar to our sample. It is possible that the weighting of indicator variables within each latent construct we developed might differ for those with other demographic characteristics. Future research is needed to replicate our results with larger samples of non-Veterans, non-whites, women, and younger persons. While our sample was large, some of our subgroups of interest (SH level ULA, bilateral ULA) were small, contributing to wide confidence intervals around certain estimates. Future research with larger samples, especially in the SH and bilateral ULA groups, are needed to estimate relationships among modeled variables with more precision.

Another limitation of our study is that all indicator variables used to estimate Extent of Prosthesis Adoption were derived from self-report questionnaires, which may be subject to recall bias. Additionally, the measures of One- and Two- handed task performance we used focused on activities requiring prehensile movements (i.e., grasping), and do not capture activities using the prosthesis in non-prehensile activities for support and bracing roles [21]. Future research might include performance data derived from wearable sensors, or engineering data logs derived from the prosthetic terminal device itself as additional objective indicators of Extent of Prosthesis Adoption construct.

Because our study design was cross-sectional, we advise caution in making causal inferences about the associations that we observed. There is likely to be some selection bias in that persons with ULA were not randomly assigned to prosthesis type but may have had a role in selecting their prosthesis type or have been prescribed a specific type of device because of clinical factors that were not measured or controlled for in our study.

There are likely more factors important to Extent of Prosthesis Adoption that we did not measure with the indicators included here. The content for the prosthesis usability measures were identified through participant input, survey analyses and the literature, and was not grounded in any specific theoretical framework. We did not solicit input on content from clinical providers such as prosthetists, or therapists. It is very possible that there are additional aspects of prosthesis usability not captured in the current measures. Our final model explained 51.5% of the variance in Extent of Prosthesis Adoption. While this is a similar amount of variance explained as in a previous report using multivariate regression to predict a composite measure of prosthesis acceptance/rejection (46%) [66], the inclusion of additional variables could potentially further improve the model fit. For example, we did not examine impact of myoelectric control strategies, such as pattern recognition, or surgical interventions, such as targeted muscle innervation, that may improve myoelectric controls and thus impact prosthesis usability experience or prosthesis adoption. Nor did we include factors such as access to prosthetic care, amount and type of prosthetic training, socio-economic status, or psychosocial factors such as presence of depressive symptomology, cognitive abilities, and aspects of the social environment (e.g., marital and employment status), which may impact upper limb use in daily life in prior studies of upper limb impairment [68–70]. It is possible these factors and other factors such as prosthesis embodiment may influence Prosthesis Usability and Extent of Prosthesis Adoption as well.

### Clinical implications

Our study provides a better understanding of factors influencing the Extent of Prosthesis Adoption. Given that Prosthesis Usability Experience is directly related to Extent of Prosthesis Adoption, we believe that it would be beneficial to collect standardized measures of Prosthesis Usability Experience at routine amputation care visits. This would enable therapists and clinicians to identify persons experiencing issues with prosthesis comfort, lack of prosthesis trust, dissatisfaction with appearance, low prosthesis desirability and poor ease of use in a timely manner, so that interventions can be developed and delivered to improve prosthesis usability experience. The goal would be to mitigate new and emerging issues that would put an individual at greater risk for poor prosthesis adoption before they resort to prosthesis abandonment. Interventions to alleviate prosthesis usability issues could include prosthetic training, socket or harness modifications, and changes to controls that may facilitate adoption.

## Conclusion

This study found that Prosthesis Usability Experience was positively associated with Extent of Prosthesis Adoption. It also identified other key relationships between demographic covariates and Extent of Prosthesis Adoption. Persons with unilateral amputation at the TH or SH level had lower Extent of Prosthesis Adoption as compared to those with TR amputation. While those with bilateral ULA had greater Extent of Prosthesis Adoption, and lower Prosthesis Usability Scores as compared to those with TR unilateral ULA. The impact of Prosthesis Usability Experience on prosthesis adoption was lessened for those with TH amputation, and for persons using myo multi-DOF prostheses. Given these findings, we believe that it would be beneficial to collect measures of the Prosthesis Usability Experience to identify persons at greater risk for poorer prosthesis adoption and better target interventions to improve prosthesis use.

## Supporting information

S1 Table Demographics of cognitive and pilot testing sample. (DOCX)

S2 Table Differential item functioning (DIF) results. (DOCX)

S3 Table Item difficulty and fit statistics from final Rasch partial credit model showing calibrations for items with Differential Item Functioning (DIF). (DOCX)

S4 Table Summary of psychometric properties of prosthesis usability experience indicator measures. (DOCX)

S5 Table Prosthesis usability experience measures correlations with other measures. (DOCX)

S1 File Original item content. (DOCX)

S2 File Cognitive interview item iterations. (DOCX)

S3 File Detailed statistical results. (DOCX)

S4 File Prosthesis usability experience final measures. (DOCX)

S5 File Prosthesis usability experience scoring crosswalks. (DOCX)

S1 Data (ZIP)
