# Supplementary material for: Prosthesis usability experience is associated with extent of upper limb prosthesis adoption: A Structural Equation Modeling (SEM) analysis
Source: PLoS One. 2024 Jun 25;19(6):e0299155. doi: 10.1371/journal.pone.0299155 (PMC11198835; doi:10.1371/journal.pone.0299155)
Supplement: S1 Table — (DOCX) [file pone.0299155.s001.docx]

|  | **Cognitive**  **N=11** | **Pilot**  **N=20** |
| --- | --- | --- |
|  | **Mn (sd)** | **Mn (sd)** |
| Age (mn, sd) | 54.4 (9.8) | 61.9 (13.5) |
|  | **N (%)** | **N (%)** |
| Gender |  |  |
| Male | 7 (63.6) | 11 (55.0) |
| Female | 4 (36.4) | 9 (45.0) |
| Amputation level |  |  |
| Transradial/wrist disarticulation | 5 (45.5) | 11 (55.0) |
| Transhumeral/elbow disarticulation | 6 (54.6) | 5 (25.0) |
| Shoulder | 0 (0.0) | 4 (20.0) |
| Bilateral upper limb loss | 1 (9.1) | 4 (20.0) |
| Prosthesis User | 9 (81.8) | 15 (75.0) |
| Primary prosthesis type |  |  |
| Body powered | 6 (66.7) | 6 (40.0) |
| Myoelectric | 2 (22.2) | 6 (40.0) |
| Hybrid | 0 (0.0) | 1 (6.7) |
| Cosmetic | 1 (11.1) | 1 (6.7) |
| Sports/recreation | 0 (0.0) | 1 (6.7) |
| Etiology |  |  |
| Combat injury | 0 (0.0) | 2 (10.0) |
| Accident | 2 (27.3) | 10 (50.0) |
| Burn | 1 (9.1) | 2 (10.0) |
| Cancer | 1 (9.1) | 1 (5.0) |
| Diabetes | 0 (0.0) | 0 (0.0) |
| Infection | 1 (9.1) | 2 (10.0) |
| Congenital | 3 (27.3) | 5 (25.0) |
| Other | 2 (18.2) | 2 (10.0) |
| Race |  |  |
| White | 10 (90.9) | 14 (70.0) |
| Black | 1 (9.1) | 1 (5.0) |
| Other | 0 (0.0) | 3 (15.0) |
| Unknown | 0 (0.0) | 2 (10.0) |

**Supplemental Table 1. Demographics of cognitive and pilot testing sample**
