# Supplementary material for: Prosthesis usability experience is associated with extent of upper limb prosthesis adoption: A Structural Equation Modeling (SEM) analysis
Source: PLoS One. 2024 Jun 25;19(6):e0299155. doi: 10.1371/journal.pone.0299155 (PMC11198835; doi:10.1371/journal.pone.0299155)
Supplement: S2 Table — (DOCX) [file pone.0299155.s002.docx]

| **Scale/Item** | **DIF contrast** | **Joint SE** | **DIF Severity** | **DIF by** | **DIF directionality: More difficult for…** |
| --- | --- | --- | --- | --- | --- |
| **Cosmesis Importance** |  |  |  |  |  |
| I prefer a prosthesis that has a natural-looking hand with fingernails | 0.93 | 0.34 | ***** | Laterality | Bilateral amputation |
| **Prosthesis Comfort (no DIF items)** |  |  |  |  |  |
| **Prosthesis Trust** |  |  |  |  |  |
| I would avoid wearing a prosthesis when caring for a baby | -0.49 | 0.14 | * | Prosthesis use | Nonusers |
| **Appearance Acceptability** |  |  |  |  |  |
| Avoid wearing a prosthesis because you do not like the fit | 0.48 | 0.20 | * | Age | Those ≤65 |
| **Prosthesis Desirability** |  |  |  |  |  |
| There are prostheses available that I like | -0.76 | 0.13 | ** | Age | Those >65 |
| A prosthesis always works for me | -0.47 | 0.15 | * | Prosthesis use | Nonusers |
| I feel that I have enough information about current prosthetic technologies | 0.69 | 0.13 | * | Prosthesis use | Users |
| **Prosthesis Ease of Use (no DIF items)** |  |  |  |  |  |

**Supplemental Table 2. Differential Item Functioning (DIF) results.**

*Slight to moderate: DIF contrast >0.43 and >2*SE (Standard Error)

**Moderate to severe: DIF contrast>0.64 and >0.43+2*SE
