# Supplementary material for: Prosthesis usability experience is associated with extent of upper limb prosthesis adoption: A Structural Equation Modeling (SEM) analysis
Source: PLoS One. 2024 Jun 25;19(6):e0299155. doi: 10.1371/journal.pone.0299155 (PMC11198835; doi:10.1371/journal.pone.0299155)
Supplement: S3 Table — (DOCX) [file pone.0299155.s003.docx]

|  | **Logit Model** | | **Infit*** | | **Outfit** | |
| --- | --- | --- | --- | --- | --- | --- |
|  | **Item Difficulty (Measure)** | **SE** | **MNSQ** | **ZSTD** | **MNSQ** | **ZSTD** |
| **Cosmesis Importance (N=703)** |  |  |  |  |  |  |
| To have a prosthesis that allows you to wear jewelry on your artificial limb, such as a watch or ring | 2.27 | 0.12 | 1.17 | 1.7 | 1.58 | 1.2 |
| To have a prosthesis that looks good with your clothing | -0.51 | 0.10 | 0.77 | -3.4 | 0.64 | -3.7 |
| I prefer a prosthesis that has a natural-looking hand with fingernails *(Unilateral only)* | -0.52 | 0.09 | 1.13 | 1.8 | 1.10 | 1.2 |
| To like the way you look while wearing your prosthesis | -1.23 | 0.10 | 0.81 | -2.8 | 0.82 | -1.7 |
| **Prosthesis Comfort (N=667)** |  |  |  |  |  |  |
| Wearing a prosthesis makes my stump uncomfortable | 0.80 | 0.08 | 0.95 | -1.0 | 0.95 | -0.9 |
| Wearing a prosthesis makes my stump hurt | 0.38 | 0.08 | 0.98 | -0.3 | 0.96 | -0.7 |
| Wearing a prosthesis makes my neck hurt | -0.50 | 0.08 | 0.99 | -0.1 | 0.99 | -0.2 |
| Wearing a prosthesis makes my back hurt | -0.68 | 0.08 | 1.02 | 0.4 | 1.01 | 0.1 |
| **Prosthesis Trust (N=726)** |  |  |  |  |  |  |
| I would avoid wearing a prosthesis when caring for a baby *(Nonuser)* | 1.31 | 0.13 | 1.02 | 0.20 | 1.07 | 0.7 |
| I would avoid wearing a prosthesis when caring for a baby *(User)* | 0.28 | 0.08 | 1.22 | 3.2 | 1.22 | 3.2 |
| I am afraid that I will scare someone, either a child or an adult, when wearing a prosthesis | -0.77 | 0.07 | 0.90 | -1.8 | 0.90 | -1.8 |
| I am afraid that I will hurt someone while wearing a prosthesis | -0.83 | 0.07 | 0.93 | -1.3 | 0.93 | -1.3 |
| **Appearance Acceptability(N=472)** |  |  |  |  |  |  |
| Avoid wearing a prosthesis because it does not fit under your clothes | -0.38 | 0.10 | 0.81 | -2.4 | 0.80 | -2.5 |
| Avoid wearing a prosthesis because of the way it fits with your clothes | -0.63 | 0.10 | 0.84 | -1.9 | 0.78 | -2.5 |
| Avoid wearing a prosthesis because you do not like the fit *(≤65)* | 0.86 | 0.13 | 1.44 | 3.5 | 1.47 | 3.7 |
| Avoid wearing a prosthesis because you do not like the fit *(>65)* | 0.15 | 0.16 | 1.20 | 1.5 | 1.20 | 1.5 |
| **Prosthesis Desirability (N=726)** |  |  |  |  |  |  |
| A prosthesis always works for me *(Nonuser)* | 0.62 | 0.14 | 1.04 | 0.4 | 1.05 | 0.4 |
| I feel that I have enough information about current prosthetic technologies *(User)* | 0.48 | 0.08 | 1.03 | 0.4 | 1.02 | 0.4 |
| A prosthesis always works for me *(User)* | 0.45 | 0.08 | 1.03 | 0.6 | 1.05 | 0.9 |
| I am satisfied with the function of the wrist of my prosthesis | 0.37 | 0.08 | 0.93 | -1.0 | 0.94 | -0.8 |
| I can get the prosthesis that I really want | 0.34 | 0.07 | 1.05 | 1.0 | 1.06 | 1.1 |
| There are prostheses available that I like *(>65)* | -0.09 | 0.10 | 0.80 | -2.7 | 0.81 | -2.5 |
| I feel that I have enough information about current prosthetic technologies *(Nonuser)* | -0.52 | 0.11 | 1.23 | 2.5 | 1.25 | 2.6 |
| There are prostheses available that suit my needs | -0.55 | 0.06 | 0.95 | -1.1 | 0.97 | -0.5 |
| There are prostheses available that I like *(≤65)* | -1.10 | 0.09 | 0.92 | -1.1 | 1.00 | 0.0 |
| **Prosthesis Ease of Use (N=416)** |  |  |  |  |  |  |
| How much physical energy did it take to use your prosthesis for as long as you needed it | 0.67 | 0.06 | 0.87 | -1.9 | 0.85 | -2.2 |
| How much mental energy did it take to use your prosthesis for as long as you needed it | 0.55 | 0.06 | 0.79 | -3.2 | 0.77 | -3.2 |
| Your prosthesis got in the way of your everyday activities | 0.13 | 0.07 | 1.29 | 3.6 | 1.32 | 3.7 |
| Off balance while wearing your prosthesis | -1.35 | 0.12 | 1.06 | 0.7 | 1.06 | 0.5 |

**Supplemental Table 3. Item difficulty and fit statistics from final Rasch Partial Credit Model showing calibrations for items with Differential Item Functioning (DIF).**

*Infit: inlier-sensitive or information-weighted fit; outfit: outlier-sensitive fit; SE: standard error; MNSQ: mean squared; ZSTD: z-standardized
