# Supplementary material for: Prosthesis usability experience is associated with extent of upper limb prosthesis adoption: A Structural Equation Modeling (SEM) analysis
Source: PLoS One. 2024 Jun 25;19(6):e0299155. doi: 10.1371/journal.pone.0299155 (PMC11198835; doi:10.1371/journal.pone.0299155)
Supplement: S4 Table — (DOCX) [file pone.0299155.s004.docx]

| **Scales** | **Final N items** | **N items (N final response categories)** | **Rasch Person reliability** | **Cronbach Alpha** | **Residual Variance Explained (1^st^ contrast Eigenvalue)** | **N (%) at Floor** | **N (%) at Ceiling** | **DIF:**  **N items (group)** | ***ICC (95% CI)** | ***MDC 90** | ***MDC 95** |
| --- | --- | --- | --- | --- | --- | --- | --- | --- | --- | --- | --- |
| Cosmesis Importance** | 4 | 3(3); 1(2) | 0.61 | 1.00 | 93.3% (1.7) | 49 (6.9) | 34 (4.8) | 1 (laterality) | 0.63 (0.43, 0.77) | 12.0 | 14.3 |
| Prosthesis Comfort | 4 | 4(4) | 0.80 | 0.86 | 55.6% (2.5) | 10 (1.5) | 34 (5.1) | 0 | 0.61 (0.40, 0.76) | 12.1 | 14.4 |
| Prosthesis Trust | 3 | 3(4) | 0.72 | 0.37 | 55.3% (1.7) | 12 (1.7) | 49 (6.8) | 1 prosthesis use) | 0.65 (0.45, 0.79) | 13.9 | 16.6 |
| Appearance Acceptability | 3 | 3(4) | 0.61 | 0.50 | 65.0% (1.9) | 2 (0.4) | 195 (41.3) | 1 (age) | 0.48 (0.24, 0.67) | 17.3 | 20.6 |
| Prosthesis Desirability | 6 | 6(4) | 0.78 | 0.81 | 55.2% (2.0) | 5 (0.7) | 9 (1.2) | 1 (age), 2 (prosthesis use^) | 0.52 (0.28,0. 69) | 15.1 | 18.0 |
| Ease of Use | 4 | 2(5); 1(4); 1(3) | 0.64 | 0.68 | 67.8 (1.7) | 2 (0.48) | 60 (14.4) | 0 | 0.56 (0.34, 0.73) | 17.1 | 20.3 |

**Supplemental Table 4.** **Summary of psychometric properties of Prosthesis Usability Experience indicator measures.**

*ICC and MDC estimates based on 50-person sample with retest data; all other columns are based off full Rasch sample (N=402)

**Cosmesis importance was dropped in SEM after the initial model

^poor fit for bilateral amputation - item only recommended for those with unilateral ULA.
