# Supplementary material for: Prosthesis usability experience is associated with extent of upper limb prosthesis adoption: A Structural Equation Modeling (SEM) analysis
Source: PLoS One. 2024 Jun 25;19(6):e0299155. doi: 10.1371/journal.pone.0299155 (PMC11198835; doi:10.1371/journal.pone.0299155)
Supplement: S5 Table — (DOCX) [file pone.0299155.s005.docx]

|  | **Cosmesis Importance** | **Prosthesis Comfort** | **Prosthesis Trust** | **Appearance Acceptability** | **Prosthesis Desirability** | **Ease of Use** |
| --- | --- | --- | --- | --- | --- | --- |
| **TAPES** | -0.17* | 0.39* | 0.23* | 0.37* | **0.59*** | 0.37* |
| **OPUS CSD 8** | 0.13* | **-0.52*** | -0.26* | -0.46* | -**0.59*** | -0.39* |
| **PROMIS UE-13 AMP** | -0.13* | 0.19* | 0.16* | 0.24* | 0.21* | 0.36* |

**Supplemental Table 5. Prosthesis Usability Experience measures correlations with other measures (N=402).**

*p<0.05, bold font indicates strong correlations (r≥0.50)
