# Supplementary material for: Prosthesis usability experience is associated with extent of upper limb prosthesis adoption: A Structural Equation Modeling (SEM) analysis
Source: PLoS One. 2024 Jun 25;19(6):e0299155. doi: 10.1371/journal.pone.0299155 (PMC11198835; doi:10.1371/journal.pone.0299155)
Supplement: S1 File — (DOCX) [file pone.0299155.s006.docx]

|  | |  |  | **Supplemental File 1.**  **Item content of Prosthesis Usability Experience Scales Prior to Cognitive Testing** | | | | | | | | |  | |  | |  |  |  |  | | **Change Yes/No** | **Change Response Options** | | **Change of Question wording** | **Change of Stem** | **New Questions** | **Questions Taken Out** |
| --- | --- | --- | --- | --- | --- | --- | --- | --- | --- | --- | --- | --- | --- | --- | --- | --- | --- | --- | --- | --- | --- | --- | --- | --- | --- | --- | --- | --- |
|  |  | | | | Cosmetic preferences | | Overall desirability of devices | Lifestyle compatibility | Adequacy of information | Confidence | Comfort of wearing | Impact on body | | Burden/ease of use | |  |  |  |  |  |  |  |  |  |  |  |  |  |
|  | How important is it for you to have a prosthesis that does not restrict the type of clothing you wear? Would you say… | | | | X |  | |  |  |  |  |  | |  | |  | | | | |  | | |  |  |  |  |  |
|  | How important is it for you to have a prosthesis that allows you to wear jewelry on your artificial limb, such as a wrist, bracelet, or ring? Would you say… | | | | X |  | |  |  |  |  |  | |  | |  | | | | |  | | |  |  |  |  |  |
|  | How important is it for you to have a prosthesis that looks good with your clothing? | | | | X |  | |  |  |  |  |  | |  | |  | | | | |  | | |  |  |  |  |  |
|  | How important is it for you to like the way you look while wearing your prosthesis? | | | | X |  | |  |  |  |  |  | |  | |  | | | | |  | | |  |  |  |  |  |
|  | Prostheses are available that suit my needs | | | |  | X | |  |  |  |  |  | |  | |  | | | | |  | | |  |  |  |  |  |
|  | Prostheses are available that I like | | | |  | X | |  |  |  |  |  | |  | |  | | | | |  | | |  |  |  |  |  |
|  | I am afraid that I will hurt someone when wearing a prosthesis | | | |  |  | |  |  | X |  |  | |  | |  | | | | |  | | |  |  |  |  |  |
|  | I am afraid that I will scare someone [either a child or an adult] when wearing a prosthesis | | | |  |  | |  |  | X |  |  | |  | |  | | | | |  | | |  |  |  |  |  |
|  | A prosthesis never works for me | | | |  | X | |  |  | X |  |  | |  | |  | | | | |  | | |  |  |  |  |  |
|  | Wearing a prosthesis makes my back hurt | | | |  |  | |  |  |  | X | X | |  | |  | | | | |  | | |  |  |  |  |  |
|  | Wearing a prosthesis makes my neck hurt | | | |  |  | |  |  |  | X | X | |  | |  | | | | |  | | |  |  |  |  |  |
|  | Wearing a prosthesis makes my stump hurt | | | |  |  | |  |  |  | X | X | |  | |  | | | | |  | | |  |  |  |  |  |
|  | I prefer a prosthesis that has a natural-looking hand with fingernails | | | | X |  | |  |  |  | X | X | |  | |  | | | | |  | | |  |  |  |  |  |
|  | I am more likely to wear a prosthesis now than when I was younger | | | |  |  | | X |  |  |  |  | |  | |  | | | | |  | | |  |  |  |  |  |
|  | I feel that I have enough information about current prosthetic technologies | | | |  |  | |  | X |  |  |  | |  | |  | | | | |  | | |  |  |  |  |  |
|  | I avoid wearing a prosthesis because I do not like the way it looks under my clothes | | | |  |  | | X |  |  |  |  | |  | |  | | | | |  | | |  |  |  |  |  |
|  | I avoid wearing a prosthesis because of the way it fits under my clothes | | | |  |  | | X |  |  |  |  | |  | |  | | | | |  | | |  |  |  |  |  |
|  | I use assistive devices or adaptive equipment to help me do everyday tasks | | | |  |  | |  |  |  |  |  | | X | |  | | | | |  | | |  |  |  |  |  |
|  | I avoid wearing a prosthesis because I do not like the harnessing. | | | |  |  | |  |  |  | X |  | |  | |  | | | | |  | | |  |  |  |  |  |
|  | Rate: how much your prosthesis slowed you down | | | |  |  | |  |  |  |  |  | | X | |  | | | | |  | | |  |  |  |  |  |
|  | Rate: how often you felt off balance while using your prosthesis | | | |  |  | |  |  |  |  |  | | X | |  | | | | |  | | |  |  |  |  |  |
|  | Rate: how much energy it took to use your prosthesis for as long as you needed it | | | |  |  | |  |  |  |  |  | | X | |  | | | | |  | | |  |  |  |  |  |
|  | Rate: How much did your prosthesis get in the way of your everyday activities? | | | |  |  | |  |  |  |  |  | | X | |  | | | | |  | | |  |  |  |  |  |
|  | Rate: how much your prosthesis slowed you down | | | |  |  | |  |  |  |  |  | | X | |  | | | | |  | | |  |  |  |  |  |
