# Supplementary material for: Prosthesis usability experience is associated with extent of upper limb prosthesis adoption: A Structural Equation Modeling (SEM) analysis
Source: PLoS One. 2024 Jun 25;19(6):e0299155. doi: 10.1371/journal.pone.0299155 (PMC11198835; doi:10.1371/journal.pone.0299155)
Supplement: S2 File — (DOCX) [file pone.0299155.s007.docx]

**Supplemental File 2.**

**Revisions Resulting from Cognitive Interviews**

The research team reviewed the item set on 4 different occasions after conducting cognitive interviews with 11 participants (9 prosthesis users and 2 non-users. Feedback from the interviews and from open ended questions was used to iteratively refine the item set as described below.

First revisions

The first changes were made after administration of the item set to 2 participants. We reversed the “importance” scale used in 5 original items (not at all important, somewhat important, very important) to correspond more intuitively with the numbering scale. The 5 items were: “ How important is it for you to have a prosthesis that does not restrict the type of clothing you wear?”, “How important is it for you to have a prosthesis that allows you to wear jewelry on your artificial limb, such as a wrist, bracelet, or ring?”, “ How important is it for you to have a prosthesis that looks good with your clothing?”, “How important is it for you to have other people like you to talk about limb loss?” and “ How important is it for you to like the way you look while wearing your prosthesis?”

We modified an item about jewelry, “How important is it for you to have a prosthesis that allows you to wear jewelry on your artificial limb, such as a wrist, bracelet, or ring?” to be more gender neutral by removing the worst bracelet, which we believed were not commonly worn by men asking instead, “How important is it for you for you to have a prosthesis that allows you to wear jewelry on your artificial limb, such as watch or ring? For clarity, we modified “I avoid wearing a prosthesis because I do not like the way it looks under my clothes” to “I avoid wearing a prosthesis because I do not like the fit.” We made this change because we learned that some participants wore their prosthesis under clothes and some wore their devices over their clothes.

We decided to word items positively because we found several participants had difficulty responding to questions that were negatively worded. Thus, we replaced the item, “A prosthesis never works for me,” with its converse, “A prosthesis always works for me.” Because harnessing is not universal to all prosthetic users, we removed this item, “I avoid wearing a prosthesis because I do not like the harnessing.” Additionally, based on the participant feedback about things that they liked or didn’t like about wearing a prosthesis, we added 3 new items: “I can get the prosthesis I really want,” “Wearing a prosthesis makes my stump uncomfortable,” and “I would avoid wearing a prosthesis when caring for a baby.” We then reordered the items so that items that were specific for non-users were grouped together.

We added one new item replaced to the concept of ease of use, “How often do you feel more balanced when wearing your prosthesis?” This item was the converse of an original item, “How often you felt off balance while using your prosthesis,” with anchors, “Not at all,” and “All the time.” The change was made because of one participant’s positive comment that her prosthesis helped her feel more balanced.

Second revision

We made further changes to the item set after administering it to 3 more participants, (2 prosthesis users and 1 non-user). Because some participants commented about their dissatisfaction with their prosthetic wrists of their prostheses. We generated another item to reflect their concerns: “I am satisfied with the wrist of my prosthesis.”

Third revision

The revised item set was then administered to another 3 participants, (2 prosthesis users and 1 non-user). We modified another item: “I avoid wearing a prosthesis because of the way it fits under my clothes” to “I avoid wearing a prosthesis because of the way it fits with my clothes.” This is because some participants wore their prosthesis over their clothes too.

We modified another item by providing examples of assistive devices to ensure that participants understood the term. The item was revised from “I use assistive devices or adaptive equipment to help me do everyday tasks” to “I use assistive devices or adaptive equipment, like a button hook or special kitchen tool, to help me do everyday tasks.”

Further, we added another item which reads, “Some things are just easier to do without a prosthesis.” This item was added because we learned that some participants preferred performing certain tasks without their prosthesis because they found it more efficient.

For further clarity, we modified the scale with anchor words from “not at all” and “very much,” and rated on a 0-4 scale, to “not at all agree” and “very much agree.”

We decided to remove an item, “How much energy it took to use your prosthesis for as long as you needed it.” We found that several participants were confused about the term, “energy,” which could mean physical or mental energy. To address this ambiguity, we replaced the original item with two new items: “How much physical energy it took to use your prosthesis for as long as you needed it,” and “How much mental energy it took to use your prosthesis for as long as you needed it.” The anchors for both newly added items were, “none” and “extreme amount.” We also clarified the item, “How much your prosthesis slowed you down,” revising it to read, “When I don’t wear my prosthesis I can get things done faster,” with anchors “not at all,” and “extreme amount.” This item, we believed was more positively worded and would be easier to answer.

Fourth administration

The revised item set was then administered to 3 more participants. No additional changes were made.
