# Supplementary material for: Prosthesis usability experience is associated with extent of upper limb prosthesis adoption: A Structural Equation Modeling (SEM) analysis
Source: PLoS One. 2024 Jun 25;19(6):e0299155. doi: 10.1371/journal.pone.0299155 (PMC11198835; doi:10.1371/journal.pone.0299155)
Supplement: S3 File — (DOCX) [file pone.0299155.s008.docx]

**Supplemental File 3**

**Detailed Statistical Results**

**Factor analyses**

Unidimensionality of the Cosmesis Importance item set was supported (CFI=0.997, TLI=0.993, RMSEA=0.069). Ratio of the 1st and second eigenvalues was 4.03 suggesting unidimensionality,^1^Cronbach alpha was 0.76.Two items related to ease of use that did not load well in exploratory factor analysis (EFA) and had poor residual correlations in confirmatory factor analysis (CFA) were dropped. The model with the final 4 items had acceptable CFI (0.974) and TLI (0.923), but poor RMSEA (0.153).

EFA of the remaining 20 items suggested a 3-factor solution. Two items (were dropped due to low loadings across all 3 scales. The first CFA of the three-factor structure identified in EFA suggested that two items should be dropped due to poor fit (loadings <0.3 and RMSEA=0.12) and that 3 items fit better if entered as a new factor. A subsequent CFA including the cosmesis importance items confirmed a 5-factor structure with acceptable fit indices and high factor loadings on each scale CFI=0.916, TLI=0.902, RMSEA=0.083. The 5 scales were labeled: Prosthesis Comfort (4 items), Prosthesis Trust (3 items), Appearance Acceptability (3 items), Prosthesis Desirability (6 items) and Cosmesis importance (5 items). All scales were designed so that higher scores indicated more of the construct measured by the scale. Thus, higher scores of Cosmesis Importance (vs. lower scores) indicate that respondents have a higher regard for the appearance of the prosthesis. Higher scores of the Prosthesis Desirability scale indicate that respondents were more satisfied with currently available prostheses. Higher scores of the Prosthesis Trust scale indicates that respondents are less likely to avoid engaging their prosthesis during specific tasks because of concerns about inadvertent consequences. Higher scores of Prosthesis Comfort indicate that wearing the prosthesis does not cause bodily pain. Finally, higher scores of Appearance Acceptability scale indicate that respondents find the fit of the prosthesis under clothes more acceptable.

**Rasch analyses**

In Rasch PCM models, category characteristic curves revealed disordered threshold parameters (due to low response of neutral categories) for 1 item in the Importance scale (‘I prefer a prosthesis that has a natural-looking hand with fingernails‘), all 7 Prosthesis Desirability items and all 3 Prosthesis Trust items. In order to correct for monotonicity issues caused by categories with low responses, categories were merged – in this case, the ‘neither disagree nor agree’ category was merged with ‘disagree’ for all items. A similar issue was observed in all items pertaining to bodily comfort. Given that these items were worded such that agreement indicated worse comfort, the neutral category was merged with ‘agree’ (the next worse choice). Finally, all 3 items of the Appearance Acceptability scale had monotonicity issues due to the low response rates for the ‘regularly’ and ‘always’ categories, so these were merged as well.

After collapsing disordered response categories, we calibrated the proposed scales using PCM, identified and dropped one misfit item (infit mnsq=1.44) which also had a low item-total correlation (r=0.32) in the Cosmesis importance scale (‘To have a prosthesis that does not restrict the type of clothing you wear. In the Prosthesis Comfort, Prosthesis Trust, Appearance Acceptability, and Prosthesis Desirability scales, all items had infit values <1.4 After dropping items, all measures had acceptable unidimensionality (with between 55.2%-93.3% of variance explained; eigenvalues of first construct were 1.7-2.5). No positive residual correlations of items were >0.4. in any scale.

Category characteristic curves also revealed disordered threshold parameters for two items in the Prosthesis Ease of Use scale. To correct this, the middle three categories for the item ‘off balance while wearing your prostheses’ were merged, and the middle and next highest category for ‘your prosthesis got in the way of your everyday activities‘ were merged. After collapsing disordered response categories, all items had infit<1.4 in the PCM (Table 3), 81.9% of residual variance was explained, and the eigenvalue of the 1^st^ contrast was 1.8, indicating acceptable unidimensionality. No items had DIF by age or gender.

**DIF Analyses**

We used two approaches to identify DIF. The first approach identified DIF based on whether DIF contrasts were greater than 0.64 or greater than $2SE+0.43$, using WINSTEPS. ^2^ The second approach was the generalized PCM Lasso method, from R package GPCMLasso.^3^ Items with moderate to severe DIF as identified by the first method and confirmed by the Lasso method were split into separate items for the relevant groups.

All items with differential item functioning (DIF) (as confirmed by Lasso methods) and the directionality of the DIF are shown in S2 Table 2. For the Importance scale there was DIF by laterality for one item. After splitting this item by laterality, the updated partial credit model indicated poor fit (infit=1.60) for the item among those with bilateral amputation – therefore we determined that the item would be used only for those with unilateral amputation. In the Prosthesis Desirability scale there was DIF by age for one item and by prosthesis use for two items. In the Confidence scale there was DIF by prosthesis use for one item. There were no items with DIF in the Prosthesis Comfort scale.-.

After adjustment for DIF, all infit values were acceptable in the updated partial credit model (Table 3), except for the Appearance Acceptability item with DIF by laterality: the infit value for this item among the younger (<65) age group was 1.44. After consideration of the borderline item fit, low number of items in the scale, Cronbach alphas and item content we decided to retain this item for the younger group. We standardized the Rasch summary score (as calculated on a logit scale) into a T-score matrix for the sample.

1. Reeve BB, Hays RD, Bjorner JB, et al. Psychometric evaluation and calibration of health-related quality of life item banks: plans for the Patient-Reported Outcomes Measurement Information System (PROMIS). *Med Care* 2007; 45: S22-31. 2007/04/20. DOI: 10.1097/01.mlr.0000250483.85507.04

00005650-200705001-00004 [pii].

2. Zwick R, Thayer, D.T., Lewis, C. An Empirical Bayes Approach to Mantel-Haenszel DIF Analysis. *Journal of Educational Measurement* 1999; 36: 1-28. DOI: doi.org/10.1111/j.1745-3984.1999.tb00543.x.

3. Schauberger G and Mair P. A regularization approach for the detection of differential item functioning in generalized partial credit models. *Behav Res Methods* 2020; 52: 279-294. 2019/03/20. DOI: 10.3758/s13428-019-01224-2.
