# Supplementary material for: Prosthesis usability experience is associated with extent of upper limb prosthesis adoption: A Structural Equation Modeling (SEM) analysis
Source: PLoS One. 2024 Jun 25;19(6):e0299155. doi: 10.1371/journal.pone.0299155 (PMC11198835; doi:10.1371/journal.pone.0299155)
Supplement: S4 File — (DOCX) [file pone.0299155.s009.docx]

**Supplemental File 4**

**Prosthesis Usability Experience Scales**

**Cosmesis Importance Scale**

Thinking of prostheses, how important are the following to you?

|  | Not at all important | Somewhat important | Very important |
| --- | --- | --- | --- |
| To have a prosthesis that allows you to wear jewelry on your artificial limb, such as a watch or ring | 1 | 2 | 3 |
| To have a prosthesis that looks good with your clothing | 1 | 2 | 3 |
| To like the way you look while wearing your prosthesis | 1 | 2 | 3 |

To what extent do you disagree or agree with each of the following statements?

|  | Strongly Disagree | Disagree | Neither Disagree nor Agree | Agree | Strongly Agree |
| --- | --- | --- | --- | --- | --- |
| I prefer a prosthesis that has a natural-looking hand with fingernails | 1 | 2 | 3 | 4 | 5 |

*To score, recode 5-category *fingernail* item to collapse neither with disagree (from 12345 to 12234). Then, for those without missing data, sum items and use scoring crosswalk.

**Prosthesis Comfort Scale**

Thinking of the prosthesis you use or have used on either side, to what extent do you disagree or agree with each of the following statements?

|  | Strongly Disagree | Disagree | Neither Disagree nor Agree | Agree | Strongly Agree |
| --- | --- | --- | --- | --- | --- |
| Wearing a prosthesis makes my back hurt | 1 | 2 | 3 | 4 | 5 |
| Wearing a prosthesis makes my neck hurt | 1 | 2 | 3 | 4 | 5 |
| Wearing a prosthesis makes my stump hurt | 1 | 2 | 3 | 4 | 5 |
| Wearing a prosthesis makes my stump uncomfortable | 1 | 2 | 3 | 4 | 5 |

*To score, reverse code all items, AND collapse neither category with agree (From 12345 to 43321). For those without missing data, sum items and use crosswalk.

**Prosthesis Trust Scale**

To what extent do you disagree or agree with each of the following statements?

|  | Strongly Disagree | Disagree | Neither Disagree nor Agree | Agree | Strongly Agree |
| --- | --- | --- | --- | --- | --- |
| I am afraid that I will hurt someone when wearing a prosthesis | 1 | 2 | 3 | 4 | 5 |
| I am afraid that I will scare someone, either a child or an adult, when wearing a prosthesis | 1 | 2 | 3 | 4 | 5 |
| I would avoid wearing a prosthesis when caring for a baby | 1 | 2 | 3 | 4 | 5 |

*To score, reverse items scoring for all 3 items so higher reflects higher trust (less avoidance) AND collapse neither category with disagree (from 12345 to 43321). For those without missing data, sum items and use crosswalks. *Separate crosswalks available for prosthesis users and nonsuers.

**Appearance Acceptability Scale**

In general, how often do you…

|  | Never | Rarely | Occasionally | Regularly | Always |
| --- | --- | --- | --- | --- | --- |
| Avoid wearing a prosthesis because you do not like the fit | 1 | 2 | 3 | 4 | 5 |
| Avoid wearing a prosthesis because it does not fit **under** your clothes. | 1 | 2 | 3 | 4 | 5 |
| Avoid wearing a prosthesis because of the way it fits **with** your clothes. | 1 | 2 | 3 | 4 | 5 |

*To score reverse coding for all items and collapse ‘regular’ and ‘always’ categories (from 12345 to 43211). For those without missing data, sum items and use scoring crosswalk.

**Prosthesis Desirability Scale**

To what extent do you disagree or agree with each of the following statements?

|  | Strongly Disagree | Disagree | Neither Disagree nor Agree | Agree | Strongly Agree |
| --- | --- | --- | --- | --- | --- |
| There are prostheses available that suit my needs | 1 | 2 | 3 | 4 | 5 |
| There are prostheses available that I like | 1 | 2 | 3 | 4 | 5 |
| A prosthesis always works for me | 1 | 2 | 3 | 4 | 5 |
| **[ASK IF USER]** I am satisfied with the function of the wrist of my prosthesis | 1 | 2 | 3 | 4 | 5 |
| I feel that I have enough information about current prosthetic technologies | 1 | 2 | 3 | 4 | 5 |
| I **can** get the prosthesis that I really want | 1 | 2 | 3 | 4 | 5 |

*To score, collapse neither category with disagree (from 12345 to 12234). For those without missing data, sum items and use crosswalk (separate crosswalks available for based on age [<65, 65+] and prosthesis use).

**Prosthesis Ease of Use Scale**

How often did you feel each of the following over the past 4 weeks?

|  | All the Time |  |  |  | Not at All |
| --- | --- | --- | --- | --- | --- |
| Off balance while wearing your prosthesis | 1 | 2 | 3 | 4 | 5 |
| That your prosthesis got in the way of your everyday activities | 1 | 2 | 3 | 4 | 5 |

Still thinking of the past 4 weeks, please answer each of the following questions.

|  | Extreme Amount |  |  |  | None |
| --- | --- | --- | --- | --- | --- |
| How much physical energy did it take to use your prosthesis for as long as you needed it? | 1 | 2 | 3 | 4 | 5 |
| How much mental energy did it take to use your prosthesis for as long as you needed it? | 1 | 2 | 3 | 4 | 5 |

*To score, recode 5-category *off balance* item to collapse middle three categories (from 12345 to 12223) AND recode 5-category *got in way of everyday activities* item to collapse second and third categories (from 12345 to 12234). Then, for those without missing data, sum items and use scoring crosswalk.
