# Supplementary material for: Prosthesis usability experience is associated with extent of upper limb prosthesis adoption: A Structural Equation Modeling (SEM) analysis
Source: PLoS One. 2024 Jun 25;19(6):e0299155. doi: 10.1371/journal.pone.0299155 (PMC11198835; doi:10.1371/journal.pone.0299155)
Supplement: S5 File — (DOCX) [file pone.0299155.s010.docx]

**Supplemental File 5**

**Scoring Crosswalks**

|  | Unilateral | |  | Bilateral | | |
| --- | --- | --- | --- | --- | --- | --- |
| Raw summed Score | T-score | Standard Error |  | | T-score | Standard Error |
| 4 | 30.71 | 7.12 |  | | 39.22 | 6.68 |
| 5 | 38.10 | 5.42 |  | | 44.75 | 4.37 |
| 6 | 44.64 | 4.07 |  | | 49.59 | 3.86 |
| 7 | 48.81 | 3.53 |  | | 53.73 | 3.66 |
| 8 | 52.17 | 3.22 |  | | 57.59 | 3.63 |
| 9 | 55.12 | 3.15 |  | | 61.83 | 4.10 |
| 10 | 58.03 | 3.19 |  | | 66.85 | 6.47 |
| 11 | 61.19 | 3.39 |  | | 39.22 | 6.68 |
| 12 | 65.15 | 4.10 |  | | 44.75 | 4.37 |
| 13 | 70.31 | 6.58 |  | | 49.59 | 3.86 |

**Scoring Crosswalk for Cosmesis Importance Scale.**

| Raw summed Score | T-score | Standard Error |
| --- | --- | --- |
| 4 | 22.13 | 7.79 |
| 5 | 27.87 | 4.71 |
| 6 | 32.17 | 3.89 |
| 7 | 35.74 | 3.81 |
| 8 | 39.30 | 3.89 |
| 9 | 43.03 | 3.89 |
| 10 | 46.72 | 3.93 |
| 11 | 50.66 | 4.14 |
| 12 | 55.04 | 4.30 |
| 13 | 59.51 | 4.18 |
| 14 | 63.73 | 4.22 |
| 15 | 68.65 | 4.96 |
| 16 | 74.84 | 7.95 |

**Scoring Crosswalk for Prosthesis Comfort Scale**

|  | Prosthesis Users | |  | Nonusers | |
| --- | --- | --- | --- | --- | --- |
| Raw summed Score | T-score | Standard Error |  | T-score | Standard Error |
| 3 | 21.29 | 8.99 |  | 21.20 | 8.99 |
| 4 | 28.57 | 5.81 |  | 28.39 | 5.76 |
| 5 | 34.93 | 5.21 |  | 34.56 | 5.07 |
| 6 | 40.41 | 4.84 |  | 39.82 | 4.75 |
| 7 | 45.16 | 4.56 |  | 44.38 | 4.47 |
| 8 | 49.68 | 4.52 |  | 48.62 | 4.42 |
| 9 | 54.24 | 4.65 |  | 52.90 | 4.52 |
| 10 | 59.40 | 5.21 |  | 57.47 | 4.65 |
| 11 | 67.14 | 6.87 |  | 62.81 | 5.48 |
| 12 | 76.41 | 9.59 |  | 69.63 | 8.85 |

**Scoring Crosswalk for Prosthesis Trust Scale**

| Raw summed Score | T-score | Standard Error |
| --- | --- | --- |
| 4 | 22.13 | 7.79 |
| 5 | 27.87 | 4.71 |
| 6 | 32.17 | 3.89 |
| 7 | 35.74 | 3.81 |
| 8 | 39.30 | 3.89 |
| 9 | 43.03 | 3.89 |
| 10 | 46.72 | 3.93 |
| 11 | 50.66 | 4.14 |
| 12 | 55.04 | 4.30 |
| 13 | 59.51 | 4.18 |
| 14 | 63.73 | 4.22 |
| 15 | 68.65 | 4.96 |
| 16 | 74.84 | 7.95 |

**Scoring Crosswalk for Appearance Acceptability Scale**

|  | Nonuser, <65 | |  | Nonuser, 65+ | | |  | | Prosthesis User, <65 | |  | Prosthesis User, 65+ | |
| --- | --- | --- | --- | --- | --- | --- | --- | --- | --- | --- | --- | --- | --- |
| Raw summed Score | T-score | Standard Error |  | T-score | Standard Error |  | | T-score | | Standard Error |  | T-score | Standard Error |
| 6 | 14.24 | 11.06 |  | 15.76 | 11.06 |  | | 15.71 | | 11.12 |  | 17.65 | 11.00 |
| 7 | 22.18 | 6.47 |  | 23.59 | 6.41 |  | | 23.71 | | 6.53 |  | 25.41 | 6.35 |
| 8 | 27.59 | 5.06 |  | 28.88 | 4.94 |  | | 29.18 | | 5.00 |  | 30.53 | 4.88 |
| 9 | 31.35 | 4.47 |  | 32.59 | 4.41 |  | | 32.88 | | 4.41 |  | 34.06 | 4.29 |
| 10 | 34.59 | 4.24 |  | 35.71 | 4.18 |  | | 35.94 | | 4.06 |  | 36.94 | 4.00 |
| 11 | 37.47 | 4.06 |  | 38.53 | 4.00 |  | | 38.65 | | 3.88 |  | 39.59 | 3.88 |
| 12 | 40.24 | 3.94 |  | 41.24 | 3.94 |  | | 41.12 | | 3.82 |  | 42.06 | 3.76 |
| 13 | 42.88 | 3.94 |  | 43.82 | 3.88 |  | | 43.53 | | 3.76 |  | 44.47 | 3.71 |
| 14 | 45.47 | 3.88 |  | 46.41 | 3.88 |  | | 45.94 | | 3.71 |  | 46.82 | 3.71 |
| 15 | 48.00 | 3.88 |  | 49.00 | 3.88 |  | | 48.29 | | 3.76 |  | 49.18 | 3.76 |
| 16 | 50.59 | 3.88 |  | 51.53 | 3.88 |  | | 50.65 | | 3.76 |  | 51.59 | 3.76 |
| 17 | 53.18 | 3.88 |  | 54.12 | 3.88 |  | | 53.12 | | 3.82 |  | 54.06 | 3.88 |
| 18 | 55.76 | 3.88 |  | 56.65 | 3.88 |  | | 55.71 | | 3.94 |  | 56.65 | 3.94 |
| 19 | 58.35 | 3.88 |  | 59.24 | 3.88 |  | | 58.47 | | 4.06 |  | 59.41 | 4.06 |
| 20 | 61.00 | 4.00 |  | 61.82 | 4.00 |  | | 61.41 | | 4.24 |  | 62.35 | 4.24 |
| 21 | 63.88 | 4.29 |  | 64.71 | 4.24 |  | | 64.65 | | 4.53 |  | 65.47 | 4.47 |
| 22 | 67.41 | 4.88 |  | 68.12 | 4.82 |  | | 68.47 | | 5.06 |  | 69.24 | 4.94 |
| 23 | 72.53 | 6.41 |  | 73.24 | 6.35 |  | | 73.88 | | 6.47 |  | 74.47 | 6.41 |
| 24 | 80.41 | 11.06 |  | 81.00 | 11.00 |  | | 81.76 | | 11.06 |  | 82.29 | 11.00 |

**Scoring Crosswalk for Prosthesis Desirability Scale.**

| Raw summed Score | T-score | Standard Error |
| --- | --- | --- |
| 4 | 22.13 | 7.79 |
| 5 | 27.87 | 4.71 |
| 6 | 32.17 | 3.89 |
| 7 | 35.74 | 3.81 |
| 8 | 39.30 | 3.89 |
| 9 | 43.03 | 3.89 |
| 10 | 46.72 | 3.93 |
| 11 | 50.66 | 4.14 |
| 12 | 55.04 | 4.30 |
| 13 | 59.51 | 4.18 |
| 14 | 63.73 | 4.22 |
| 15 | 68.65 | 4.96 |
| 16 | 74.84 | 7.95 |
| 17 | 67.65 | 11.36 |

**Scoring Crosswalk for Prosthesis Ease of Use Scale**
