# Supplementary material for: Prosthesis usability experience is associated with extent of upper limb prosthesis adoption: A Structural Equation Modeling (SEM) analysis
Source: PLoS One. 2024 Jun 25;19(6):e0299155. doi: 10.1371/journal.pone.0299155 (PMC11198835; doi:10.1371/journal.pone.0299155)
Supplement: S1 Data — (ZIP) [file pone.0299155.s011.zip › Data Collection Instrument.docx]

**Data Collection Instrument**

**Screener and Survey**

**Notes on how to read this survey document:**

- Text in **BOLD CAPS** in brackets represents programming instructions, which will not be visible to respondents.
- Response options in all caps indicate text that will not be read by the interviewer unless otherwise instructed. Lower case response options indicate that text will be read by the interviewer.
- **[SP] = Single punch question** - only one response allowed; variable name matches question number.
- **[MP] = Multi punch question** - more than one response allowed; variable created for each response option.
  - Naming convention used is *‘question number_response option code’* (Example: U_F2_1)
  - SAS formats: 1 = Checked, 0 = Unchecked.
- **Grid questions (MP Down, SP Across)**:
  - Variable created for each row in the grid
  - Naming convention used is *‘question number_letter of the row’* (Example: U_B3_a; U_B3_b)
- **Skip logic –** noted before each question (Example: [ASK IF S1=1 (YES)]). Questions skipped because of logic will have responses that appear as ‘.’ (missing data). They do not have a separate code.
- ***Note:*** *Question numbers in blue font represent the associated question from the Aim 1 survey; an “R” after the number indicates a revision.*

**Part A: Screener**

**[ASK ALL] [SP]**

A1. Do you have an upper limb amputation or limb difference at the wrist or higher?

1 Yes

2 No

98 DON’T KNOW/NOT SURE [DO NOT READ]

99 REFUSED [DO NOT READ]

**[ASK IF A1=1 (YES UPPER LIMB AMPUTATION/LIMB DIFFERENCE) AND RECRUITMENT=3 OR 5 (EBLAST OR OTHER PARTICIPANT)]**

A2. Is it a congenital limb difference?

1 Yes

2 No

98 DON’T KNOW/NOT SURE [DO NOT READ]

99 REFUSED [DO NOT READ]

**[ASK IF A1=1 (YES UPPER LIMB AMPUTATION/LIMB DIFFERENCE)] [SP]**

A3. On which side is your upper limb amputation? Please select one response.

1 Right side

2 Left side

3 Both sides

98 DON’T KNOW/NOT SURE [DO NOT READ]

99 REFUSED [DO NOT READ]

**[ASK IF A3=1 OR 2 (UNILATERAL AMPUTEE)] [SP]**

A4. [U_C5] Do you currently use a prosthesis?

1 YES

2 NO

98 DON’T KNOW/NOT SURE [DO NOT READ]

99 REFUSED [DO NOT READ]

**[ASK IF A4=2 (NO, DO NOT CURRENTLY USE A PROSTHESIS)] [SP]**

A5. [U_C1] Have you ever used a prosthesis?

1 YES

2 NO

98 DON’T KNOW/NOT SURE [DO NOT READ]

99 REFUSED [DO NOT READ]

**[ASK IF A3=3 (BILATERAL AMPUTEE)] [SP]**

A6. [B_C5] Do you currently use a prosthesis on your right side?

1 YES

2 NO

98 DON’T KNOW/NOT SURE [DO NOT READ]

99 REFUSED [DO NOT READ]

**[ASK IF A3=3 (BILATERAL AMPUTEE)] [SP]**

A7. [B_C27] Do you currently use a prosthesis on your left side?

1 YES

2 NO

98 DON’T KNOW/NOT SURE [DO NOT READ]

99 REFUSED [DO NOT READ]

**[ASK IF A6=2 (NO, DO NOT CURRENTLY USE A PROSTHESIS ON RIGHT SIDE] [SP]**

A8. [B_C1R] Have you ever used a prosthesis on your right side?

1 YES

2 NO

98 DON’T KNOW/NOT SURE [DO NOT READ]

99 REFUSED [DO NOT READ]

**[ASK IF A7=2 (NO, DO NOT CURRENTLY USE A PROSTHESIS ON LEFT SIDE] [SP]**

A9. [B_C1R] Have you ever used a prosthesis on your left side?

1 YES

2 NO

98 DON’T KNOW/NOT SURE [DO NOT READ]

99 REFUSED [DO NOT READ]

**Survey**

**Part B: Amputation History**

**[ASK ALL] [SP]**

U_B1. [U_B1] At what level is your upper limb amputation?

1 Chest wall level, sometimes called “forequarter”

2 At the shoulder joint

3 Above the elbow

4 At the elbow

5 Below the elbow

6 At the wrist joint

7 Through the hand

98 DON’T KNOW/NOT SURE [DO NOT READ]

99 REFUSED [DO NOT READ]

**[SHOW IF UB1=7 (THROUGH THE HAND)]**

**INELIGIBLE_NOAMP2. [SP]**

Those are all the questions we have for you. Unfortunately, you are not eligible to participate in our study. Thank you for your time.

**[ASK IF A2 NOT EQUAL TO 1 (YES, CONGENITAL LIMB DIFFERENCE)] [DROP DOWN BOX: MONTHS, RANGE JANUARY-DECEMBER]**

U_B2a. [U_B2a] What was the month and year of your initial amputation?

MONTH:______

98 DON’T KNOW/NOT SURE [DO NOT READ]

99 REFUSED [DO NOT READ]

**[ASK IF A2 NOT EQUAL TO 1 (YES, CONGENITAL LIMB DIFFERENCE)] [SHOW ON SAME SCREEN AS U_B2A] [OPEN-ENDED NUMERIC]**

U_B2b. [U_B2b]

YEAR: ______

98 DON’T KNOW/NOT SURE [DO NOT READ]

99 REFUSED [DO NOT READ]

**Part C: Prosthetic Use**

**[ASK IF USER] [SP]**

U_C7. [U_C6] How many hours a day do you typically use a prosthesis?

1 Less than 2 hours a day

2 2 to less than 4 hours a day

3 4 to less than 8 hours a day

4 8 to less than 12 hours a day

5 12 hours or more a day

98 DON’T KNOW/NOT SURE [DO NOT READ]

99 REFUSED [DO NOT READ]

U_C10. [U_C9R] What type of prosthesis do you currently use?

1 Body powered, meaning you open and close the terminal device using a harness

2 Myoelectric

3 Hybrid

4 Cosmetic

5 Sport

98 DON’T KNOW/NOT SURE [DO NOT READ]

99 REFUSED [DO NOT READ]

U_C12. [U_C11R] How often do you use this device? Would you say…

1. Daily
2. 2 to 3 times per week
3. Once a week
4. A few times a month
5. Once a month

6 Once every few months

7 1 to 2 times per year

98 DON’T KNOW/NOT SURE [DO NOT READ]

99 REFUSED [DO NOT READ]

**[ASK IF USER] [SP]**

U_C25. [U_C22] Thinking of the primary prosthesis and terminal device combination you use now, did you receive training to use it?

1 YES

2 NO

98 DON’T KNOW/NOT SURE [DO NOT READ]

99 REFUSED [DO NOT READ]

**Part D: OPUS UEFS**

**[ASK IF USER] [SP]**

U_D1. [NEW] Now please think of the past 4 weeks. Have you used your prosthesis in the past 4 weeks?

1 YES

2 NO

98 DON’T KNOW/NOT SURE [DO NOT READ]

99 REFUSED [DO NOT READ]

**[ASK IF USER AND U_D1=1 (YES, USED PROSTHESIS IN PAST 4 WEEKS)] [SP]**

U_D1a. [NEW] The first activity I want to ask about is: **Put toothpaste on a brush and brush your teeth**. Did you do this or attempt to do this in the past 4 weeks with the assistance of your prosthesis?

1 YES

2 NO

98 DON’T KNOW/NOT SURE [DO NOT READ]

99 REFUSED [DO NOT READ]

**[ASK IF U_D1a=1 (YES, DID/ATTEMPTED ACTIVITY)] [SP]**

U_D1b. [NEW] How easy or difficult was it for you to: **Put toothpaste on a brush and brush your teeth**? Would you say…

1 Very easy

2 Easy

3 Difficult

4 Very difficult

5 Could not do at all

98 DON’T KNOW/NOT SURE [DO NOT READ]

99 REFUSED [DO NOT READ]

**[ASK IF USER AND U_D1=1 (YES, USED PROSTHESIS IN PAST 4 WEEKS)] [SP]**

U_D2a. [NEW] **Wash your face** with the assistance of your prosthesis. [READ IF NECESSARY: Did you do this or attempt to do this in the past 4 weeks with the assistance of your prosthesis?]

1 YES

2 NO

98 DON’T KNOW/NOT SURE [DO NOT READ]

99 REFUSED [DO NOT READ]

**[ASK IF U_D2a=1 (YES, DID/ATTEMPTED ACTIVITY)] [SP]**

U_D2b. [NEW] How easy or difficult was it [READ IF NECESSARY: for you to **wash your face**? Would you say…]

1 Very easy

2 Easy

3 Difficult

4 Very difficult

5 Could not do at all

98 DON’T KNOW/NOT SURE [DO NOT READ]

99 REFUSED [DO NOT READ]

**[ASK IF USER AND U_D1=1 (YES, USED PROSTHESIS IN PAST 4 WEEKS)] [SP]**

U_D3a. [NEW] **Brush or comb your hair**. Did you do this or attempt to do this in the past 4 weeks with the assistance of your prosthesis?

1 YES

2 NO

98 DON’T KNOW/NOT SURE [DO NOT READ]

99 REFUSED [DO NOT READ]

**[ASK IF U_D3a=1 (YES, DID/ATTEMPTED ACTIVITY)] [SP]**

U_D3b. [NEW] How easy or difficult was it for you to: **Brush or comb your hair**? Would you say…

1 Very easy

2 Easy

3 Difficult

4 Very difficult

5 Could not do at all

98 DON’T KNOW/NOT SURE [DO NOT READ]

99 REFUSED [DO NOT READ]

**[ASK IF USER AND U_D1=1 (YES, USED PROSTHESIS IN PAST 4 WEEKS)] [SP]**

U_D4a. [NEW] **Fold a bath towel** with the assistance of your prosthesis. [READ IF NECESSARY: Did you do this or attempt to do this in the past 4 weeks with the assistance of your prosthesis?]

1 YES

2 NO

98 DON’T KNOW/NOT SURE [DO NOT READ]

99 REFUSED [DO NOT READ]

**[ASK IF U_D4a=1 (YES, DID/ATTEMPTED ACTIVITY)] [SP]**

U_D4b. [NEW] How easy or difficult was it [READ IF NECESSARY: for you to **fold a bath towel**? Would you say…]

1 Very easy

2 Easy

3 Difficult

4 Very difficult

5 Could not do at all

98 DON’T KNOW/NOT SURE [DO NOT READ]

99 REFUSED [DO NOT READ]

**[ASK IF USER AND U_D1=1 (YES, USED PROSTHESIS IN PAST 4 WEEKS) AND SAMPLE VARIABLE SEX=FEMALE] [SP]**

U_D5a. [NEW] **Fasten a bra** with the assistance of your prosthesis. [READ IF NECESSARY: Did you do this or attempt to do this in the past 4 weeks with the assistance of your prosthesis?]

1 YES

2 NO

98 DON’T KNOW/NOT SURE [DO NOT READ]

99 REFUSED [DO NOT READ]

**[ASK IF U_D5a=1 (YES, DID/ATTEMPTED ACTIVITY)] [SP]**

U_D5b. [NEW] How easy or difficult was it [READ IF NECESSARY: for you to **fasten a bra**? Would you say…]

1 Very easy

2 Easy

3 Difficult

4 Very difficult

5 Could not do at all

98 DON’T KNOW/NOT SURE [DO NOT READ]

99 REFUSED [DO NOT READ]

**[ASK IF USER AND U_D1=1 (YES, USED PROSTHESIS IN PAST 4 WEEKS)] [SP]**

U_D6a. [NEW] **Put on underwear** with the assistance of your prosthesis. [READ IF NECESSARY: Did you do this or attempt to do this in the past 4 weeks with the assistance of your prosthesis?]

1 YES

2 NO

98 DON’T KNOW/NOT SURE [DO NOT READ]

99 REFUSED [DO NOT READ]

**[ASK IF U_D6a=1 (YES, DID/ATTEMPTED ACTIVITY)] [SP]**

U_D6b. [NEW] How easy or difficult was it [READ IF NECESSARY: for you to **put on underwear**? Would you say…]

1 Very easy

2 Easy

3 Difficult

4 Very difficult

5 Could not do at all

98 DON’T KNOW/NOT SURE [DO NOT READ]

99 REFUSED [DO NOT READ]

**[ASK IF USER AND U_D1=1 (YES, USED PROSTHESIS IN PAST 4 WEEKS)] [SP]**

U_D7a. [NEW] **Put on and remove a T-shirt** with the assistance of your prosthesis. [READ IF NECESSARY: Did you do this or attempt to do this in the past 4 weeks with the assistance of your prosthesis?]

1 YES

2 NO

98 DON’T KNOW/NOT SURE [DO NOT READ]

99 REFUSED [DO NOT READ]

**[ASK IF U_D7a=1 (YES, DID/ATTEMPTED ACTIVITY)] [SP]**

U_D7b. [NEW] How easy or difficult was it for you to: **Put on and remove a T-shirt**? Would you say…

1 Very easy

2 Easy

3 Difficult

4 Very difficult

5 Could not do at all

98 DON’T KNOW/NOT SURE [DO NOT READ]

99 REFUSED [DO NOT READ]

**[ASK IF USER AND U_D1=1 (YES, USED PROSTHESIS IN PAST 4 WEEKS)] [SP]**

U_D8a. [NEW] **Button a shirt with front buttons** with the assistance of your prosthesis. [READ IF NECESSARY: Did you do this or attempt to do this in the past 4 weeks with the assistance of your prosthesis?]

1 YES

2 NO

98 DON’T KNOW/NOT SURE [DO NOT READ]

99 REFUSED [DO NOT READ]

**[ASK IF U_D8a=1 (YES, DID/ATTEMPTED ACTIVITY)] [SP]**

U_D8b. [NEW] How easy or difficult was it [READ IF NECESSARY: for you to **Button a shirt with front buttons**? Would you say…]

1 Very easy

2 Easy

3 Difficult

4 Very difficult

5 Could not do at all

98 DON’T KNOW/NOT SURE [DO NOT READ]

99 REFUSED [DO NOT READ]

**[ASK IF USER AND U_D1=1 (YES, USED PROSTHESIS IN PAST 4 WEEKS)] [SP]**

U_D9a. [NEW] **Put on socks** with the assistance of your prosthesis. [READ IF NECESSARY: Did you do this or attempt to do this in the past 4 weeks with the assistance of your prosthesis?]

1 YES

2 NO

98 DON’T KNOW/NOT SURE [DO NOT READ]

99 REFUSED [DO NOT READ]

**[ASK IF U_D9a=1 (YES, DID/ATTEMPTED ACTIVITY)] [SP]**

U_D9b. [NEW] How easy or difficult was it [READ IF NECESSARY: for you to **Put on socks**? Would you say…]

1 Very easy

2 Easy

3 Difficult

4 Very difficult

5 Could not do at all

98 DON’T KNOW/NOT SURE [DO NOT READ]

99 REFUSED [DO NOT READ]

**[ASK IF USER AND U_D1=1 (YES, USED PROSTHESIS IN PAST 4 WEEKS)] [SP]**

U_D10a. [NEW] **Tie shoe laces** with the assistance of your prosthesis. [READ IF NECESSARY: Did you do this or attempt to do this in the past 4 weeks with the assistance of your prosthesis?]

1 YES

2 NO

98 DON’T KNOW/NOT SURE [DO NOT READ]

99 REFUSED [DO NOT READ]

**[ASK IF U_D10a=1 (YES, DID/ATTEMPTED ACTIVITY)] [SP]**

U_D10b. [NEW] How easy or difficult was it [READ IF NECESSARY: for you to **tie shoe laces**? Would you say…]

1 Very easy

2 Easy

3 Difficult

4 Very difficult

5 Could not do at all

98 DON’T KNOW/NOT SURE [DO NOT READ]

99 REFUSED [DO NOT READ]

**[ASK IF USER AND U_D1=1 (YES, USED PROSTHESIS IN PAST 4 WEEKS)] [SP]**

U_D11a. [NEW] **Attach the end of a zipper and zip a jacket** with the assistance of your prosthesis. [READ IF NECESSARY: Did you do this or attempt to do this in the past 4 weeks with the assistance of your prosthesis?]

1 YES

2 NO

98 DON’T KNOW/NOT SURE [DO NOT READ]

99 REFUSED [DO NOT READ]

**[ASK IF U_D11a=1 (YES, DID/ATTEMPTED ACTIVITY)] [SP]**

U_D11b. [NEW] How easy or difficult was it [READ IF NECESSARY: for you to **Attach the end of a zipper and zip a jacket**? Would you say…]

1 Very easy

2 Easy

3 Difficult

4 Very difficult

5 Could not do at all

98 DON’T KNOW/NOT SURE [DO NOT READ]

99 REFUSED [DO NOT READ]

**[ASK IF USER AND U_D1=1 (YES, USED PROSTHESIS IN PAST 4 WEEKS)] [SP]**

U_D12a. [NEW] **Drink from a paper cup** with the assistance of your prosthesis. [READ IF NECESSARY: Did you do this or attempt to do this in the past 4 weeks with the assistance of your prosthesis?]

1 YES

2 NO

98 DON’T KNOW/NOT SURE [DO NOT READ]

99 REFUSED [DO NOT READ]

**[ASK IF U_D12a=1 (YES, DID/ATTEMPTED ACTIVITY)] [SP]**

U_D12b. [NEW] How easy or difficult was it [READ IF NECESSARY: for you to **drink from a paper cup**? Would you say…]

1 Very easy

2 Easy

3 Difficult

4 Very difficult

5 Could not do at all

98 DON’T KNOW/NOT SURE [DO NOT READ]

99 REFUSED [DO NOT READ]

**[ASK IF USER AND U_D1=1 (YES, USED PROSTHESIS IN PAST 4 WEEKS)] [SP]**

U_D13a. [NEW] **Eat with a fork or spoon** with the assistance of your prosthesis. [READ IF NECESSARY: Did you do this or attempt to do this in the past 4 weeks with the assistance of your prosthesis?]

1 YES

2 NO

98 DON’T KNOW/NOT SURE [DO NOT READ]

99 REFUSED [DO NOT READ]

**[ASK IF U_D13a=1 (YES, DID/ATTEMPTED ACTIVITY)] [SP]**

U_D13b. [NEW] How easy or difficult was it [READ IF NECESSARY: for you to **eat with a fork or spoon**? Would you say…]

1 Very easy

2 Easy

3 Difficult

4 Very difficult

5 Could not do at all

98 DON’T KNOW/NOT SURE [DO NOT READ]

99 REFUSED [DO NOT READ]

**[ASK IF USER AND U_D1=1 (YES, USED PROSTHESIS IN PAST 4 WEEKS)] [SP]**

U_D14a. [NEW] **Eat a deli sandwich** with the assistance of your prosthesis. [READ IF NECESSARY: Did you do this or attempt to do this in the past 4 weeks with the assistance of your prosthesis?]

1 YES

2 NO

98 DON’T KNOW/NOT SURE [DO NOT READ]

99 REFUSED [DO NOT READ]

**[ASK IF U_D14a=1 (YES, DID/ATTEMPTED ACTIVITY)] [SP]**

U_D14b. [NEW] How easy or difficult was it [READ IF NECESSARY: for you to **eat a deli sandwich**? Would you say…]

1 Very easy

2 Easy

3 Difficult

4 Very difficult

5 Could not do at all

98 DON’T KNOW/NOT SURE [DO NOT READ]

99 REFUSED [DO NOT READ]

**[ASK IF USER AND U_D1=1 (YES, USED PROSTHESIS IN PAST 4 WEEKS)] [SP]**

U_D15a. [NEW] **Cut meat with a knife and fork** with the assistance of your prosthesis. [READ IF NECESSARY: Did you do this or attempt to do this in the past 4 weeks with the assistance of your prosthesis?]

1 YES

2 NO

98 DON’T KNOW/NOT SURE [DO NOT READ]

99 REFUSED [DO NOT READ]

**[ASK IF U_D15a=1 (YES, DID/ATTEMPTED ACTIVITY)] [SP]**

U_D15b. [NEW] How easy or difficult was it [READ IF NECESSARY: for you to **cut meat with a knife and fork**? Would you say…]

1 Very easy

2 Easy

3 Difficult

4 Very difficult

5 Could not do at all

98 DON’T KNOW/NOT SURE [DO NOT READ]

99 REFUSED [DO NOT READ]

**[ASK IF USER AND U_D1=1 (YES, USED PROSTHESIS IN PAST 4 WEEKS)] [SP]**

U_D16a. [NEW] **Chop vegetables at a counter** with the assistance of your prosthesis. [READ IF NECESSARY: Did you do this or attempt to do this in the past 4 weeks with the assistance of your prosthesis?]

1 YES

2 NO

98 DON’T KNOW/NOT SURE [DO NOT READ]

99 REFUSED [DO NOT READ]

**[ASK IF U_D16a=1 (YES, DID/ATTEMPTED ACTIVITY)] [SP]**

U_D16b. [NEW] How easy or difficult was it [READ IF NECESSARY: for you to **chop vegetables at a counter**? Would you say…]

1 Very easy

2 Easy

3 Difficult

4 Very difficult

5 Could not do at all

98 DON’T KNOW/NOT SURE [DO NOT READ]

99 REFUSED [DO NOT READ]

**[ASK IF USER AND U_D1=1 (YES, USED PROSTHESIS IN PAST 4 WEEKS)] [SP]**

U_D17a. [NEW] **Spread peanut butter on fresh bread, using your prosthesis**. [READ IF NECESSARY: Did you do this or attempt to do this in the past 4 weeks with the assistance of your prosthesis?]

1 YES

2 NO

98 DON’T KNOW/NOT SURE [DO NOT READ]

99 REFUSED [DO NOT READ]

**[ASK IF U_D17a=1 (YES, DID/ATTEMPTED ACTIVITY)] [SP]**

U_D17b. [NEW] How easy or difficult was it [READ IF NECESSARY: for you to **Spread peanut butter on fresh bread, using your prosthesis**? Would you say…]

1 Very easy

2 Easy

3 Difficult

4 Very difficult

5 Could not do at all

98 DON’T KNOW/NOT SURE [DO NOT READ]

99 REFUSED [DO NOT READ]

**[ASK IF USER AND U_D1=1 (YES, USED PROSTHESIS IN PAST 4 WEEKS)] [SP]**

U_D18a. [NEW] **Pour from a 12 ounce can with the assistance of your prosthesis without spilling or crushing it**. [READ IF NECESSARY: Did you do this or attempt to do this in the past 4 weeks with the assistance of your prosthesis?]

1 YES

2 NO

98 DON’T KNOW/NOT SURE [DO NOT READ]

99 REFUSED [DO NOT READ]

**[ASK IF U_D18a=1 (YES, DID/ATTEMPTED ACTIVITY)] [SP]**

U_D18b. [NEW] How easy or difficult was it [READ IF NECESSARY: for you to **pour from a 12 ounce can without spilling or crushing it**? Would you say…]

1 Very easy

2 Easy

3 Difficult

4 Very difficult

5 Could not do at all

98 DON’T KNOW/NOT SURE [DO NOT READ]

99 REFUSED [DO NOT READ]

**[ASK IF USER AND U_D1=1 (YES, USED PROSTHESIS IN PAST 4 WEEKS)] [SP]**

U_D19a. [NEW] **Hold a small bottle to twist off the lid** with the assistance of your prosthesis. [READ IF NECESSARY: Did you do this or attempt to do this in the past 4 weeks with the assistance of your prosthesis?]

1 YES

2 NO

98 DON’T KNOW/NOT SURE [DO NOT READ]

99 REFUSED [DO NOT READ]

**[ASK IF U_D19a=1 (YES, DID/ATTEMPTED ACTIVITY)] [SP]**

U_D19b. [NEW] How easy or difficult was it [READ IF NECESSARY: for you to **hold a small bottle to twist off the lid**? Would you say…]

1 Very easy

2 Easy

3 Difficult

4 Very difficult

5 Could not do at all

98 DON’T KNOW/NOT SURE [DO NOT READ]

99 REFUSED [DO NOT READ]

**[ASK IF USER AND U_D1=1 (YES, USED PROSTHESIS IN PAST 4 WEEKS)] [SP]**

U_D20a. [NEW] **Hold a bowl to stir** with the assistance of your prosthesis. [READ IF NECESSARY: Did you do this or attempt to do this in the past 4 weeks with the assistance of your prosthesis?]

1 YES

2 NO

98 DON’T KNOW/NOT SURE [DO NOT READ]

99 REFUSED [DO NOT READ]

**[ASK IF U_D20a=1 (YES, DID/ATTEMPTED ACTIVITY)] [SP]**

U_D20b. [NEW] How easy or difficult was it [READ IF NECESSARY: for you to **hold a bowl to stir**? Would you say…]

1 Very easy

2 Easy

3 Difficult

4 Very difficult

5 Could not do at all

98 DON’T KNOW/NOT SURE [DO NOT READ]

99 REFUSED [DO NOT READ]

**[ASK IF USER AND U_D1=1 (YES, USED PROSTHESIS IN PAST 4 WEEKS)] [SP]**

U_D21a. [NEW] **Hold potatoes or fruit to peel them** with the assistance of your prosthesis. [READ IF NECESSARY: Did you do this or attempt to do this in the past 4 weeks with the assistance of your prosthesis?]

1 YES

2 NO

98 DON’T KNOW/NOT SURE [DO NOT READ]

99 REFUSED [DO NOT READ]

**[ASK IF U_D21a=1 (YES, DID/ATTEMPTED ACTIVITY)] [SP]**

U_D21b. [NEW] How easy or difficult was it [READ IF NECESSARY: for you to **hold potatoes or fruit to peel them**? Would you say…]

1 Very easy

2 Easy

3 Difficult

4 Very difficult

5 Could not do at all

98 DON’T KNOW/NOT SURE [DO NOT READ]

99 REFUSED [DO NOT READ]

**[ASK IF USER AND U_D1=1 (YES, USED PROSTHESIS IN PAST 4 WEEKS)] [SP]**

U_D22a. [NEW] **Open a bag of chips using both hands** with the assistance of your prosthesis. [READ IF NECESSARY: Did you do this or attempt to do this in the past 4 weeks with the assistance of your prosthesis?]

1 YES

2 NO

98 DON’T KNOW/NOT SURE [DO NOT READ]

99 REFUSED [DO NOT READ]

**[ASK IF U_D22a=1 (YES, DID/ATTEMPTED ACTIVITY)] [SP]**

U_D22b. [NEW] How easy or difficult was it [READ IF NECESSARY: for you to **open a bag of chips using both hands**? Would you say…]

1 Very easy

2 Easy

3 Difficult

4 Very difficult

5 Could not do at all

98 DON’T KNOW/NOT SURE [DO NOT READ]

99 REFUSED [DO NOT READ]

**[ASK IF USER AND U_D1=1 (YES, USED PROSTHESIS IN PAST 4 WEEKS)] [SP]**

U_D23a. [NEW] **Hold an envelope to open it** with the assistance of your prosthesis. [READ IF NECESSARY: Did you do this or attempt to do this in the past 4 weeks with the assistance of your prosthesis?]

1 YES

2 NO

98 DON’T KNOW/NOT SURE [DO NOT READ]

99 REFUSED [DO NOT READ]

**[ASK IF U_D23a=1 (YES, DID/ATTEMPTED ACTIVITY)] [SP]**

U_D23b. [NEW] How easy or difficult was it [READ IF NECESSARY: for you to **hold an envelope to open it**? Would you say…]

1 Very easy

2 Easy

3 Difficult

4 Very difficult

5 Could not do at all

98 DON’T KNOW/NOT SURE [DO NOT READ]

99 REFUSED [DO NOT READ]

**[ASK IF USER AND U_D1=1 (YES, USED PROSTHESIS IN PAST 4 WEEKS)] [SP]**

U_D24a. [NEW] **Type on a computer keyboard** with the assistance of your prosthesis. [READ IF NECESSARY: Did you do this or attempt to do this in the past 4 weeks with the assistance of your prosthesis?]

1 YES

2 NO

98 DON’T KNOW/NOT SURE [DO NOT READ]

99 REFUSED [DO NOT READ]

**[ASK IF U_D24a=1 (YES, DID/ATTEMPTED ACTIVITY)] [SP]**

U_D24b. [NEW] How easy or difficult was it [READ IF NECESSARY: for you to **type on a computer keyboard**? Would you say…]

1 Very easy

2 Easy

3 Difficult

4 Very difficult

5 Could not do at all

98 DON’T KNOW/NOT SURE [DO NOT READ]

99 REFUSED [DO NOT READ]

**[ASK IF USER AND U_D1=1 (YES, USED PROSTHESIS IN PAST 4 WEEKS)] [SP]**

U_D25a. [NEW] **Write your name legibly** with the assistance of your prosthesis. [READ IF NECESSARY: Did you do this or attempt to do this in the past 4 weeks with the assistance of your prosthesis?]

1 YES

2 NO

98 DON’T KNOW/NOT SURE [DO NOT READ]

99 REFUSED [DO NOT READ]

**[ASK IF U_D25a=1 (YES, DID/ATTEMPTED ACTIVITY)] [SP]**

U_D25b. [NEW] How easy or difficult was it [READ IF NECESSARY: for you to **write your name legibly**? Would you say…]

1 Very easy

2 Easy

3 Difficult

4 Very difficult

5 Could not do at all

98 DON’T KNOW/NOT SURE [DO NOT READ]

99 REFUSED [DO NOT READ]

**[ASK IF USER AND U_D1=1 (YES, USED PROSTHESIS IN PAST 4 WEEKS)] [SP]**

U_D26a. [NEW] **Take a dollar bill out of a wallet** with the assistance of your prosthesis. [READ IF NECESSARY: Did you do this or attempt to do this in the past 4 weeks with the assistance of your prosthesis?]

1 YES

2 NO

98 DON’T KNOW/NOT SURE [DO NOT READ]

99 REFUSED [DO NOT READ]

**[ASK IF U_D26a=1 (YES, DID/ATTEMPTED ACTIVITY)] [SP]**

U_D26b. [NEW] How easy or difficult was it [READ IF NECESSARY: for you to **take a dollar bill out of a wallet**? Would you say…]

1 Very easy

2 Easy

3 Difficult

4 Very difficult

5 Could not do at all

98 DON’T KNOW/NOT SURE [DO NOT READ]

99 REFUSED [DO NOT READ]

**[ASK IF USER AND U_D1=1 (YES, USED PROSTHESIS IN PAST 4 WEEKS)] [SP]**

U_D27a. [NEW] **Use scissors** with the assistance of your prosthesis. [READ IF NECESSARY: Did you do this or attempt to do this in the past 4 weeks with the assistance of your prosthesis?]

1 YES

2 NO

98 DON’T KNOW/NOT SURE [DO NOT READ]

99 REFUSED [DO NOT READ]

**[ASK IF U_D27a=1 (YES, DID/ATTEMPTED ACTIVITY)] [SP]**

U_D27b. [NEW] How easy or difficult was it [READ IF NECESSARY: for you to **use scissors**? Would you say…]

1 Very easy

2 Easy

3 Difficult

4 Very difficult

5 Could not do at all

98 DON’T KNOW/NOT SURE [DO NOT READ]

99 REFUSED [DO NOT READ]

**[ASK IF USER AND U_D1=1 (YES, USED PROSTHESIS IN PAST 4 WEEKS)] [SP]**

U_D28a. [NEW] **Use a key in a lock** with the assistance of your prosthesis. [READ IF NECESSARY: Did you do this or attempt to do this in the past 4 weeks with the assistance of your prosthesis?]

1 YES

2 NO

98 DON’T KNOW/NOT SURE [DO NOT READ]

99 REFUSED [DO NOT READ]

**[ASK IF U_D28a=1 (YES, DID/ATTEMPTED ACTIVITY)] [SP]**

U_D28b. [NEW] How easy or difficult was it [READ IF NECESSARY: for you to **use a key in a lock**? Would you say…]

1 Very easy

2 Easy

3 Difficult

4 Very difficult

5 Could not do at all

98 DON’T KNOW/NOT SURE [DO NOT READ]

99 REFUSED [DO NOT READ]

**[ASK IF USER AND U_D1=1 (YES, USED PROSTHESIS IN PAST 4 WEEKS)] [SP]**

U_D29a. [NEW] **Open a door with a round knob** with the assistance of your prosthesis. [READ IF NECESSARY: Did you do this or attempt to do this in the past 4 weeks with the assistance of your prosthesis?]

1 YES

2 NO

98 DON’T KNOW/NOT SURE [DO NOT READ]

99 REFUSED [DO NOT READ]

**[ASK IF U_D29a=1 (YES, DID/ATTEMPTED ACTIVITY)] [SP]**

U_D29b. [NEW] How easy or difficult was it [READ IF NECESSARY: for you to **open a door with a round knob**? Would you say…]

1 Very easy

2 Easy

3 Difficult

4 Very difficult

5 Could not do at all

98 DON’T KNOW/NOT SURE [DO NOT READ]

99 REFUSED [DO NOT READ]

**[ASK IF USER AND U_D1=1 (YES, USED PROSTHESIS IN PAST 4 WEEKS)] [SP]**

U_D30a. [NEW] **Hold a nail to hammer** with the assistance of your prosthesis. [READ IF NECESSARY: Did you do this or attempt to do this in the past 4 weeks with the assistance of your prosthesis?]

1 YES

2 NO

98 DON’T KNOW/NOT SURE [DO NOT READ]

99 REFUSED [DO NOT READ]

**[ASK IF U_D30a=1 (YES, DID/ATTEMPTED ACTIVITY)] [SP]**

U_D30b. [NEW] How easy or difficult was it [READ IF NECESSARY: for you to **hold a nail to hammer**? Would you say…]

1 Very easy

2 Easy

3 Difficult

4 Very difficult

5 Could not do at all

98 DON’T KNOW/NOT SURE [DO NOT READ]

99 REFUSED [DO NOT READ]

**[ASK IF USER AND U_D1=1 (YES, USED PROSTHESIS IN PAST 4 WEEKS)] [SP]**

U_D31a. [NEW] **Carry a laundry basket** with the assistance of your prosthesis. [READ IF NECESSARY: Did you do this or attempt to do this in the past 4 weeks with the assistance of your prosthesis?]

1 YES

2 NO

98 DON’T KNOW/NOT SURE [DO NOT READ]

99 REFUSED [DO NOT READ]

**[ASK IF U_D31a=1 (YES, DID/ATTEMPTED ACTIVITY)] [SP]**

U_D31b. [NEW] How easy or difficult was it [READ IF NECESSARY: for you to **carry a laundry basket**? Would you say…]

1 Very easy

2 Easy

3 Difficult

4 Very difficult

5 Could not do at all

98 DON’T KNOW/NOT SURE [DO NOT READ]

99 REFUSED [DO NOT READ]

**[ASK IF USER AND U_D1=1 (YES, USED PROSTHESIS IN PAST 4 WEEKS)] [SP]**

U_D32a. [NEW] **Lift a shopping bag containing about 10 pounds, using your prosthesis only**. [READ IF NECESSARY: Did you do this or attempt to do this in the past 4 weeks with the assistance of your prosthesis?]

1 YES

2 NO

98 DON’T KNOW/NOT SURE [DO NOT READ]

99 REFUSED [DO NOT READ]

**[ASK IF U_D32a=1 (YES, DID/ATTEMPTED ACTIVITY)] [SP]**

U_D32b. [NEW] How easy or difficult was it [READ IF NECESSARY: for you to **Lift a shopping bag containing about 10 pounds, using your prosthesis only**? Would you say…]

1 Very easy

2 Easy

3 Difficult

4 Very difficult

5 Could not do at all

98 DON’T KNOW/NOT SURE [DO NOT READ]

99 REFUSED [DO NOT READ]

**[ASK IF USER AND U_D1=1 (YES, USED PROSTHESIS IN PAST 4 WEEKS)] [SP]**

U_D33a. [NEW] **Lift a shopping bag containing about 20 pounds, using your prosthesis**. [READ IF NECESSARY: Did you do this or attempt to do this in the past 4 weeks with the assistance of your prosthesis?]

1 YES

2 NO

98 DON’T KNOW/NOT SURE [DO NOT READ]

99 REFUSED [DO NOT READ]

**[ASK IF U_D33a=1 (YES, DID/ATTEMPTED ACTIVITY)] [SP]**

U_D33b. [NEW] How easy or difficult was it [READ IF NECESSARY: for you to **Lift a shopping bag containing about 20 pounds, using your prosthesis**? Would you say…]

1 Very easy

2 Easy

3 Difficult

4 Very difficult

5 Could not do at all

98 DON’T KNOW/NOT SURE [DO NOT READ]

99 REFUSED [DO NOT READ]

**[ASK IF USER AND U_D1=1 (YES, USED PROSTHESIS IN PAST 4 WEEKS)] [SP]**

U_D34a. [NEW] **Lift and carry bulky objects like grocery bags or crates that weigh more than 15 pounds, using your prosthesis**. [READ IF NECESSARY: Did you do this or attempt to do this in the past 4 weeks with the assistance of your prosthesis?]

1 YES

2 NO

98 DON’T KNOW/NOT SURE [DO NOT READ]

99 REFUSED [DO NOT READ]

**[ASK IF U_D34a=1 (YES, DID/ATTEMPTED ACTIVITY)] [SP]**

U_D34b. [NEW] How easy or difficult was it [READ IF NECESSARY: for you to **Lift and carry bulky objects like grocery bags or crates that weigh more than 15 pounds, using your prosthesis**? Would you say…]

1 Very easy

2 Easy

3 Difficult

4 Very difficult

5 Could not do at all

98 DON’T KNOW/NOT SURE [DO NOT READ]

99 REFUSED [DO NOT READ]

**Part G: Prosthetic Acceptance**

**[ASK IF USER] [GRID; MP DOWN, SP ACROSS]**

U_G1 [NEW] Thinking of prostheses, how important are the following to you?

|  | Not at all important | Somewhat important | Very important | DON’T KNOW/NOT SURE/NOT APPLICABLE [DO NOT READ] | REFUSED [DO NOT READ] |
| --- | --- | --- | --- | --- | --- |
| 1. To have a prosthesis that does not restrict the type of clothing you wear [Would you say Not at All Important, Somewhat Important, or Very Important?] | 0 | 1 | 2 | 98 | 99 |
| 1. To have a prosthesis that allows you to wear jewelry on your artificial limb, such as a watch or ring | 0 | 1 | 2 | 98 | 99 |
| 1. To have a prosthesis that looks good with your clothing | 0 | 1 | 2 | 98 | 99 |
| 1. To like the way you look while wearing your prosthesis | 0 | 1 | 2 | 98 | 99 |

**[ASK ALL] [GRID; MP DOWN, SP ACROSS]**

U_G2 [NEW] To what extent do you disagree or agree with each of the following statements?

|  | Strongly Disagree | Disagree | Neither Disagree nor Agree | Agree | Strongly Agree | DON’T KNOW/ NOT SURE/NOT APPLICABLE [DO NOT READ] | REFUSED [DO NOT READ] |
| --- | --- | --- | --- | --- | --- | --- | --- |
| 1. There are prostheses available that suit my needs [Would you say Strongly Disagree, Disagree, Neither Disagree nor Agree, Agree, or Strongly Agree?] | 1 | 2 | 3 | 4 | 5 | 98 | 99 |
| 1. There are prostheses available that I like | 1 | 2 | 3 | 4 | 5 | 98 | 99 |
| 1. I am afraid that I will hurt someone when wearing a prosthesis | 1 | 2 | 3 | 4 | 5 | 98 | 99 |
| 1. I am afraid that I will scare someone, either a child or an adult, when wearing a prosthesis | 1 | 2 | 3 | 4 | 5 | 98 | 99 |
| 1. I would avoid wearing a prosthesis when caring for a baby | 1 | 2 | 3 | 4 | 5 | 98 | 99 |
| 1. **[ASK IF USER OR NON_USER]** A prosthesis always works for me | 1 | 2 | 3 | 4 | 5 | 98 | 99 |
| 1. **[ASK IF USER OR NON_USER]** Wearing a prosthesis makes my back hurt | 1 | 2 | 3 | 4 | 5 | 98 | 99 |
| 1. **[ASK IF USER OR NON_USER]** Wearing a prosthesis makes my neck hurt | 1 | 2 | 3 | 4 | 5 | 98 | 99 |
| 1. **[ASK IF USER OR NON_USER]** Wearing a prosthesis makes my stump hurt | 1 | 2 | 3 | 4 | 5 | 98 | 99 |
| 1. **[ASK IF USER OR NON_USER]** Wearing a prosthesis makes my stump uncomfortable | 1 | 2 | 3 | 4 | 5 | 98 | 99 |
| 1. **[ASK IF USER]** I am satisfied with the function of the wrist of my prosthesis | 1 | 2 | 3 | 4 | 5 | 98 | 99 |
| 1. I prefer a prosthesis that has a natural-looking hand with fingernails | 1 | 2 | 3 | 4 | 5 | 98 | 99 |
| 1. **[ASK IF USER OR NON_USER]** I am more likely to wear a prosthesis now than when I was younger | 1 | 2 | 3 | 4 | 5 | 98 | 99 |
| 1. I feel that I have enough information about current prosthetic technologies | 1 | 2 | 3 | 4 | 5 | 98 | 99 |
| 1. I **can** get the prosthesis that I really want | 1 | 2 | 3 | 4 | 5 | 98 | 99 |
| 1. Some things are just easier to do without a prosthesis | 1 | 2 | 3 | 4 | 5 | 98 | 99 |

**[ASK ALL] [GRID; MP DOWN, SP ACROSS]**

U_G3 [NEW]

**[ASK IF USER]** In general, how often do you do each of the following?

**[ASK IF NON_USER OR NEVER_USER]** In general, how often do you…

|  | Never | Rarely | Occasionally | Regularly | Always | DON’T KNOW/ NOT SURE [DO NOT READ] | REFUSED [DO NOT READ] |
| --- | --- | --- | --- | --- | --- | --- | --- |
| 1. **[ASK IF USER]** Avoid wearing a prosthesis because you do not like the fit [Would you say Never, Rarely, Occasionally, Regularly, or Always?] | 0 | 1 | 2 | 3 | 4 | 98 | 99 |
| 1. **[ASK IF USER]** Avoid wearing a prosthesis because it does not fit **under** your clothes. | 0 | 1 | 2 | 3 | 4 | 98 | 99 |
| 1. **[ASK IF USER]** Avoid wearing a prosthesis because of the way it fits **with** your clothes. | 0 | 1 | 2 | 3 | 4 | 98 | 99 |
| 1. Use assistive devices or adaptive equipment, like a button hook, or special kitchen tool, to help you do everyday tasks | 0 | 1 | 2 | 3 | 4 | 98 | 99 |

**[ASK IF USER] [SP]**

U_G4. [NEW] Do you pay any out-of-pocket expenses related to your prosthesis?

1 YES

2 NO

98 DON’T KNOW/NOT SURE [DO NOT READ]

99 REFUSED [DO NOT READ]

**[ASK IF NON_USER] [SP]**

U_G4a. [NEW] Have you ever paid any out-of-pocket expenses related to a prosthesis?

1 YES

2 NO

98 DON’T KNOW/NOT SURE [DO NOT READ]

99 REFUSED [DO NOT READ]

**[ASK IF U_G4=1 OR U_G4a =1 (YES, PAY OUT-OF-POCKET EXPENSES)] [GRID; MP DOWN, SP ACROSS]**

U_G5 [NEW] To what extent do you disagree or agree with each of the following statements?

|  | Strongly Disagree | Disagree | Neither Disagree nor Agree | Agree | Strongly Agree | DON’T KNOW/ NOT SURE [DO NOT READ] | REFUSED [DO NOT READ] |
| --- | --- | --- | --- | --- | --- | --- | --- |
| 1. I can afford the out-of-pocket expenses to purchase a prosthesis [Would you say Strongly Disagree, Disagree, Neither Disagree nor Agree, Agree, or Strongly Agree?] | 1 | 2 | 3 | 4 | 5 | 98 | 99 |
| 1. I can afford the out-of-pocket expenses to maintain a prosthesis | 1 | 2 | 3 | 4 | 5 | 98 | 99 |
| 1. I can afford the out-of-pocket expenses to repair a prosthesis as soon as needed | 1 | 2 | 3 | 4 | 5 | 98 | 99 |
| 1. I can afford the out-of-pocket expenses to replace a prosthesis as soon as needed | 1 | 2 | 3 | 4 | 5 | 98 | 99 |

**Part N: Questions about You**

**[ASK ALL] [SP]**

U_N1. [U_N3] What is your current military status?

1 Veteran

2 Civilian

3 Active Duty

98 DON’T KNOW/NOT SURE [DO NOT READ]

99 REFUSED [DO NOT READ]

**[ASK ALL] [MP]**

U_N4. [U_N7A] What is your race? I will read you a list of choices. You may choose one or more.

**[**CHECK ALL THAT APPLY**]**

1 White or Caucasian

2 Black or African-American

3 Asian

4 American Indian or Alaska Native

5 Native Hawaiian or Pacific Islander

6 Other [Please explain]

98 DON’T KNOW/NOT SURE [DO NOT READ]

99 REFUSED [DO NOT READ]

**[ASK IF U_N4=6 (OTHER)] [TEXT BOX]**

U_N4_SPECIFY. __________________________________

**Part O: Follow-Up Permission**

**[ASK ALL] [SP]**

U_O1.  [U_O1] Thank you for taking the time to complete this survey. May we contact you in the future to see if you are interested in participating in other study activities?

1 Yes

2 No

98 DON’T KNOW/NOT SURE [DO NOT READ]

99 REFUSED [DO NOT READ]

**Part B: Amputation History**

**[ASK ALL] [SP]**

B_B1. [U_B1] At what level is your upper limb amputation on your right side?

1 Chest wall level, sometimes called “forequarter”

2 At the shoulder joint

3 Above the elbow

4 At the elbow

5 Below the elbow

6 At the wrist joint

7 Through the hand

98 DON’T KNOW/NOT SURE [DO NOT READ]

99 REFUSED [DO NOT READ]

**[ASK IF A2 NOT EQUAL TO 1 (YES, CONGENITAL LIMB DIFFERENCE)] [DROP DOWN BOX: MONTHS, RANGE JANUARY-DECEMBER]**

B_B2a. [U_B2a] What was the month and year of your initial amputation on your right side?

MONTH:______

98 DON’T KNOW/NOT SURE [DO NOT READ]

99 REFUSED [DO NOT READ]

**[ASK IF A2 NOT EQUAL TO 1 (YES, CONGENITAL LIMB DIFFERENCE)] [SHOW ON SAME SCREEN AS B_B2A] [OPEN-ENDED NUMERIC]**

B_B2b. [U_B2b]

YEAR: ______

98 DON’T KNOW/NOT SURE [DO NOT READ]

99 REFUSED [DO NOT READ]

**[ASK ALL] [SP]**

B_B4. [U_B1] At what level is your upper limb amputation on your left side?

1 Chest wall level, sometimes called “forequarter”

2 At the shoulder joint

3 Above the elbow

4 At the elbow

5 Below the elbow

6 At the wrist joint

7 Through the hand

98 DON’T KNOW/NOT SURE [DO NOT READ]

99 REFUSED [DO NOT READ]

**[SHOW IF B_B1=7 AND B_B4=7 (THROUGH THE HAND ON BOTH SIDES)]**

**B_INELIGIBLE_NOAMP2 [SP]**

Those are all the questions we have for you. Unfortunately, you are not eligible to participate in our study. Thank you for your time.

**[ASK IF A2 NOT EQUAL TO 1 (YES, CONGENITAL LIMB DIFFERENCE)] [DROP DOWN BOX: MONTHS, RANGE JANUARY-DECEMBER]**

B_B5a. [U_B2a] What was the month and year of your initial amputation on your left side?

MONTH:______

98 DON’T KNOW/NOT SURE [DO NOT READ]

99 REFUSED [DO NOT READ]

**[ASK IF A2 NOT EQUAL TO 1 (YES, CONGENITAL LIMB DIFFERENCE)] [SHOW ON SAME SCREEN AS B_B5A] [OPEN-ENDED NUMERIC]**

B_B5b. [U_B2b]

YEAR: ______

98 DON’T KNOW/NOT SURE [DO NOT READ]

99 REFUSED [DO NOT READ]

**Part C: Prosthetic Use**

**[ASK IF A8=2 (NO, NEVER USED PROSTHESIS ON RIGHT SIDE) OR A9=2 (NO, NEVER USED PROSTHESIS ON LEFT SIDE)] [SP]**

**[ASK IF A6=1 (YES, CURRENTLY USE PROSTHESIS ON RIGHT)] [SP]**

B_C8. [U_C7] How many hours a day do you typically use a prosthesis on your right side?

1 Less than 2 hours a day

2 2 to less than 4 hours a day

3 4 to less than 8 hours a day

4 8 to less than 12 hours a day

5 12 hours or more a day

98 DON’T KNOW/NOT SURE [DO NOT READ]

99 REFUSED [DO NOT READ]

**[ASK IF B_C10=1 (ONE TYPE OF PROSTHESIS ON RIGHT)] [SP]**

B_C11. [U_C10] What type of prosthesis do you currently use on your right side?

1 Body powered, meaning you open and close the terminal device using a harness

2 Myoelectric

3 Hybrid

4 Cosmetic

5 Sport

98 DON’T KNOW/NOT SURE [DO NOT READ]

99 REFUSED [DO NOT READ]

**[ASK IF B_C10=1 (ONE TYPE OF PROSTHESIS ON RIGHT)] [SP]**

B_C13. [U_C12] How often do you use this device that is for your right side? Would you say…

1. Daily
2. 2 to 3 times per week
3. Once a week
4. A few times a month
5. Once a month
6. Once every few months
7. 1 to 2 times per year

98 DON’T KNOW/NOT SURE [DO NOT READ]

99 REFUSED [DO NOT READ]

**[ASK IF A6=1 (YES, CURRENTLY USE PROSTHESIS ON RIGHT)] [SP]**

B_C26. [U_C25] Thinking of the primary prosthesis and terminal device combination you use now on your right side, did you receive training to use it?

1 YES

2 NO

98 DON’T KNOW/NOT SURE [DO NOT READ]

99 REFUSED [DO NOT READ]

**[ASK IF A7=1 (YES, CURRENTLY USE PROSTHESIS ON LEFT)] [SP]**

B_C28. [U_C7] How many hours a day do you typically use a prosthesis on your left side?

1 Less than 2 hours a day

2 2 to less than 4 hours a day

3 4 to less than 8 hours a day

4 8 to less than 12 hours a day

5 12 hours or more a day

98 DON’T KNOW/NOT SURE [DO NOT READ]

99 REFUSED [DO NOT READ]

**[ASK IF B_C30=1 (ONE TYPE OF PROSTHESIS ON LEFT)] [SP]**

B_C31. [U_C10] What type of prosthesis do you currently use on your left side?

1 Body powered, meaning you open and close the terminal device using a harness

2 Myoelectric

3 Hybrid

4 Cosmetic

5 Sport

98 DON’T KNOW/NOT SURE [DO NOT READ]

99 REFUSED [DO NOT READ]

**[ASK IF B_C30=1 (ONE TYPE OF PROSTHESIS ON LEFT)] [SP]**

B_C33. [U_C12] How often do you use this device that is for your left side? Would you say…

1. Daily
2. 2 to 3 times per week
3. Once a week
4. A few times a month
5. Once a month
6. Once every few months
7. 1 to 2 times per year

98 DON’T KNOW/NOT SURE [DO NOT READ]

99 REFUSED [DO NOT READ]

**[ASK IF A7=1 (YES, CURRENTLY USE PROSTHESIS ON LEFT)] [SP]**

B_C46. [U_C25] Thinking of the primary prosthesis and terminal device combination you use now on your left side, did you receive training to use it?

1 YES

2 NO

98 DON’T KNOW/NOT SURE [DO NOT READ]

99 REFUSED [DO NOT READ]

**Part D: OPUS UEFS**

**[ASK ALL] [SP]**

B_D0. For this question, please think of your dominant side. If you do not believe you have a dominant side, please think of the side you use most often. What is your dominant side now?

1 LEFT

2 RIGHT

3 BOTH

98 DON’T KNOW/NOT SURE [DO NOT READ]

99 REFUSED [DO NOT READ]

**[ASK IF USER] [SP]**

B_D1. [U_D1] Now please think of the past 4 weeks. Have you used your prosthesis on your dominant side in the past 4 weeks?

1 YES

2 NO

98 DON’T KNOW/NOT SURE [DO NOT READ]

99 REFUSED [DO NOT READ]

**[ASK IF USER AND B_D1=1 (YES, USED PROSTHESIS ON DOMINANT SIDE IN PAST 4 WEEKS)] [SP]**

B_D1a. [U_D1a] The first activity I want to ask about is: **Put toothpaste on a brush and brush your teeth**. Did you do this or attempt to do this in the past 4 weeks with the assistance of your prosthesis?

1 YES

2 NO

98 DON’T KNOW/NOT SURE [DO NOT READ]

99 REFUSED [DO NOT READ]

**[ASK IF B_D1a=1 (YES, DID/ATTEMPTED ACTIVITY)] [SP]**

B_D1b. [U_D1b] How easy or difficult was it for you to: **Put toothpaste on a brush and brush your teeth**? Would you say…

1 Very easy

2 Easy

3 Difficult

4 Very difficult

5 Could not do at all

98 DON’T KNOW/NOT SURE [DO NOT READ]

99 REFUSED [DO NOT READ]

**[ASK IF USER AND B_D1=1 (YES, USED PROSTHESIS ON DOMINANT SIDE IN PAST 4 WEEKS)] [SP]**

B_D2a. [U_D2a] **Wash your face** with the assistance of your prosthesis. [READ IF NECESSARY: Did you do this or attempt to do this in the past 4 weeks with the assistance of your prosthesis?]

1 YES

2 NO

98 DON’T KNOW/NOT SURE [DO NOT READ]

99 REFUSED [DO NOT READ]

**[ASK IF B_D2a=1 (YES, DID/ATTEMPTED ACTIVITY)] [SP]**

B_D2b. [U_D2b] How easy or difficult was it [READ IF NECESSARY: for you to **wash your face**? Would you say…]

1 Very easy

2 Easy

3 Difficult

4 Very difficult

5 Could not do at all

98 DON’T KNOW/NOT SURE [DO NOT READ]

99 REFUSED [DO NOT READ]

**[ASK IF USER AND B_D1=1 (YES, USED PROSTHESIS ON DOMINANT SIDE IN PAST 4 WEEKS)] [SP]**

B_D3a. [U_D3a] **Brush or comb your hair**. Did you do this or attempt to do this in the past 4 weeks with the assistance of your prosthesis?

1 YES

2 NO

98 DON’T KNOW/NOT SURE [DO NOT READ]

99 REFUSED [DO NOT READ]

**[ASK IF B_D3a=1 (YES, DID/ATTEMPTED ACTIVITY)] [SP]**

B_D3b. [U_D3b] How easy or difficult was it for you to: **Brush or comb your hair**? Would you say…

1 Very easy

2 Easy

3 Difficult

4 Very difficult

5 Could not do at all

98 DON’T KNOW/NOT SURE [DO NOT READ]

99 REFUSED [DO NOT READ]

**[ASK IF USER AND B_D1=1 (YES, USED PROSTHESIS ON DOMINANT SIDE IN PAST 4 WEEKS)] [SP]**

B_D4a. [U_D4a] **Fold a bath towel** with the assistance of your prosthesis. [READ IF NECESSARY: Did you do this or attempt to do this in the past 4 weeks with the assistance of your prosthesis?]

1 YES

2 NO

98 DON’T KNOW/NOT SURE [DO NOT READ]

99 REFUSED [DO NOT READ]

**[ASK IF B_D4a=1 (YES, DID/ATTEMPTED ACTIVITY)] [SP]**

B_D4b. [U_D4b] How easy or difficult was it [READ IF NECESSARY: for you to **fold a bath towel**? Would you say…]

1 Very easy

2 Easy

3 Difficult

4 Very difficult

5 Could not do at all

98 DON’T KNOW/NOT SURE [DO NOT READ]

99 REFUSED [DO NOT READ]

**[ASK IF USER AND B_D1=1 (YES, USED PROSTHESIS ON DOMINANT SIDE IN PAST 4 WEEKS) AND SAMPLE VARIABLE SEX=FEMALE] [SP]**

B_D5a. [U_D5a] **Fasten a bra** with the assistance of your prosthesis. [READ IF NECESSARY: Did you do this or attempt to do this in the past 4 weeks with the assistance of your prosthesis?]

1 YES

2 NO

98 DON’T KNOW/NOT SURE [DO NOT READ]

99 REFUSED [DO NOT READ]

**[ASK IF B_D5a=1 (YES, DID/ATTEMPTED ACTIVITY)] [SP]**

B_D5b. [U_D5b] How easy or difficult was it [READ IF NECESSARY: for you to **fasten a bra**? Would you say…]

1 Very easy

2 Easy

3 Difficult

4 Very difficult

5 Could not do at all

98 DON’T KNOW/NOT SURE [DO NOT READ]

99 REFUSED [DO NOT READ]

**[ASK IF USER AND B_D1=1 (YES, USED PROSTHESIS ON DOMINANT SIDE IN PAST 4 WEEKS)] [SP]**

B_D6a. [U_D6a] **Put on underwear** with the assistance of your prosthesis. [READ IF NECESSARY: Did you do this or attempt to do this in the past 4 weeks with the assistance of your prosthesis?]

1 YES

2 NO

98 DON’T KNOW/NOT SURE [DO NOT READ]

99 REFUSED [DO NOT READ]

**[ASK IF B_D6a=1 (YES, DID/ATTEMPTED ACTIVITY)] [SP]**

B_D6b. [U_D6b] How easy or difficult was it [READ IF NECESSARY: for you to **put on underwear**? Would you say…]

1 Very easy

2 Easy

3 Difficult

4 Very difficult

5 Could not do at all

98 DON’T KNOW/NOT SURE [DO NOT READ]

99 REFUSED [DO NOT READ]

**[ASK IF USER AND B_D1=1 (YES, USED PROSTHESIS ON DOMINANT SIDE IN PAST 4 WEEKS)] [SP]**

B_D7a. [U_D7a] **Put on and remove a T-shirt** with the assistance of your prosthesis. [READ IF NECESSARY: Did you do this or attempt to do this in the past 4 weeks with the assistance of your prosthesis?]

1 YES

2 NO

98 DON’T KNOW/NOT SURE [DO NOT READ]

99 REFUSED [DO NOT READ]

**[ASK IF B_D7a=1 (YES, DID/ATTEMPTED ACTIVITY)] [SP]**

B_D7b. [U_D7b] How easy or difficult was it for you to: **Put on and remove a T-shirt**? Would you say…

1 Very easy

2 Easy

3 Difficult

4 Very difficult

5 Could not do at all

98 DON’T KNOW/NOT SURE [DO NOT READ]

99 REFUSED [DO NOT READ]

**[ASK IF USER AND B_D1=1 (YES, USED PROSTHESIS ON DOMINANT SIDE IN PAST 4 WEEKS)] [SP]**

B_D8a. [U_D8a] **Button a shirt with front buttons** with the assistance of your prosthesis. [READ IF NECESSARY: Did you do this or attempt to do this in the past 4 weeks with the assistance of your prosthesis?]

1 YES

2 NO

98 DON’T KNOW/NOT SURE [DO NOT READ]

99 REFUSED [DO NOT READ]

**[ASK IF B_D8a=1 (YES, DID/ATTEMPTED ACTIVITY)] [SP]**

B_D8b. [U_D8b] How easy or difficult was it [READ IF NECESSARY: for you to **Button a shirt with front buttons**? Would you say…]

1 Very easy

2 Easy

3 Difficult

4 Very difficult

5 Could not do at all

98 DON’T KNOW/NOT SURE [DO NOT READ]

99 REFUSED [DO NOT READ]

**[ASK IF USER AND B_D1=1 (YES, USED PROSTHESIS ON DOMINANT SIDE IN PAST 4 WEEKS)] [SP]**

B_D9a. [B_D9a] **Put on socks** with the assistance of your prosthesis. [READ IF NECESSARY: Did you do this or attempt to do this in the past 4 weeks with the assistance of your prosthesis?]

1 YES

2 NO

98 DON’T KNOW/NOT SURE [DO NOT READ]

99 REFUSED [DO NOT READ]

**[ASK IF B_D9a=1 (YES, DID/ATTEMPTED ACTIVITY)] [SP]**

B_D9b. [U_D9b] How easy or difficult was it [READ IF NECESSARY: for you to **Put on socks**? Would you say…]

1 Very easy

2 Easy

3 Difficult

4 Very difficult

5 Could not do at all

98 DON’T KNOW/NOT SURE [DO NOT READ]

99 REFUSED [DO NOT READ]

**[ASK IF USER AND B_D1=1 (YES, USED PROSTHESIS ON DOMINANT SIDE IN PAST 4 WEEKS)] [SP]**

B_D10a. [U_D10a] **Tie shoe laces** with the assistance of your prosthesis. [READ IF NECESSARY: Did you do this or attempt to do this in the past 4 weeks with the assistance of your prosthesis?]

1 YES

2 NO

98 DON’T KNOW/NOT SURE [DO NOT READ]

99 REFUSED [DO NOT READ]

**[ASK IF B_D10a=1 (YES, DID/ATTEMPTED ACTIVITY)] [SP]**

B_D10b. [U_10b] How easy or difficult was it [READ IF NECESSARY: for you to **tie shoe laces**? Would you say…]

1 Very easy

2 Easy

3 Difficult

4 Very difficult

5 Could not do at all

98 DON’T KNOW/NOT SURE [DO NOT READ]

99 REFUSED [DO NOT READ]

**[ASK IF USER AND B_D1=1 (YES, USED PROSTHESIS ON DOMINANT SIDE IN PAST 4 WEEKS)] [SP]**

B_D11a. [U_D11a] **Attach the end of a zipper and zip a jacket** with the assistance of your prosthesis. [READ IF NECESSARY: Did you do this or attempt to do this in the past 4 weeks with the assistance of your prosthesis?]

1 YES

2 NO

98 DON’T KNOW/NOT SURE [DO NOT READ]

99 REFUSED [DO NOT READ]

**[ASK IF B_D11a=1 (YES, DID/ATTEMPTED ACTIVITY)] [SP]**

B_D11b. [U_D11b] How easy or difficult was it [READ IF NECESSARY: for you to **Attach the end of a zipper and zip a jacket**? Would you say…]

1 Very easy

2 Easy

3 Difficult

4 Very difficult

5 Could not do at all

98 DON’T KNOW/NOT SURE [DO NOT READ]

99 REFUSED [DO NOT READ]

**[ASK IF USER AND B_D1=1 (YES, USED PROSTHESIS ON DOMINANT SIDE IN PAST 4 WEEKS)] [SP]**

B_D12a. [U_D12a] **Drink from a paper cup** with the assistance of your prosthesis. [READ IF NECESSARY: Did you do this or attempt to do this in the past 4 weeks with the assistance of your prosthesis?]

1 YES

2 NO

98 DON’T KNOW/NOT SURE [DO NOT READ]

99 REFUSED [DO NOT READ]

**[ASK IF B_D12a=1 (YES, DID/ATTEMPTED ACTIVITY)] [SP]**

B_D12b. [U_D12b] How easy or difficult was it [READ IF NECESSARY: for you to **drink from a paper cup**? Would you say…]

1 Very easy

2 Easy

3 Difficult

4 Very difficult

5 Could not do at all

98 DON’T KNOW/NOT SURE [DO NOT READ]

99 REFUSED [DO NOT READ]

**[ASK IF USER AND B_D1=1 (YES, USED PROSTHESIS ON DOMINANT SIDE IN PAST 4 WEEKS)] [SP]**

B_D13a. [U_D13a] **Eat with a fork or spoon** with the assistance of your prosthesis. [READ IF NECESSARY: Did you do this or attempt to do this in the past 4 weeks with the assistance of your prosthesis?]

1 YES

2 NO

98 DON’T KNOW/NOT SURE [DO NOT READ]

99 REFUSED [DO NOT READ]

**[ASK IF B_D13a=1 (YES, DID/ATTEMPTED ACTIVITY)] [SP]**

B_D13b. [U_D13b] How easy or difficult was it [READ IF NECESSARY: for you to **eat with a fork or spoon**? Would you say…]

1 Very easy

2 Easy

3 Difficult

4 Very difficult

5 Could not do at all

98 DON’T KNOW/NOT SURE [DO NOT READ]

99 REFUSED [DO NOT READ]

**[ASK IF USER AND B_D1=1 (YES, USED PROSTHESIS ON DOMINANT SIDE IN PAST 4 WEEKS)] [SP]**

B_D14a. [U_D14a] **Eat a deli sandwich** with the assistance of your prosthesis. [READ IF NECESSARY: Did you do this or attempt to do this in the past 4 weeks with the assistance of your prosthesis?]

1 YES

2 NO

98 DON’T KNOW/NOT SURE [DO NOT READ]

99 REFUSED [DO NOT READ]

**[ASK IF B_D14a=1 (YES, DID/ATTEMPTED ACTIVITY)] [SP]**

B_D14b. [U_D14b] How easy or difficult was it [READ IF NECESSARY: for you to **eat a deli sandwich**? Would you say…]

1 Very easy

2 Easy

3 Difficult

4 Very difficult

5 Could not do at all

98 DON’T KNOW/NOT SURE [DO NOT READ]

99 REFUSED [DO NOT READ]

**[ASK IF USER AND B_D1=1 (YES, USED PROSTHESIS ON DOMINANT SIDE IN PAST 4 WEEKS)] [SP]**

B_D15a. [U_D15a] **Cut meat with a knife and fork** with the assistance of your prosthesis. [READ IF NECESSARY: Did you do this or attempt to do this in the past 4 weeks with the assistance of your prosthesis?]

1 YES

2 NO

98 DON’T KNOW/NOT SURE [DO NOT READ]

99 REFUSED [DO NOT READ]

**[ASK IF B_D15a=1 (YES, DID/ATTEMPTED ACTIVITY)] [SP]**

B_D15b. [U_D15b] How easy or difficult was it [READ IF NECESSARY: for you to **cut meat with a knife and fork**? Would you say…]

1 Very easy

2 Easy

3 Difficult

4 Very difficult

5 Could not do at all

98 DON’T KNOW/NOT SURE [DO NOT READ]

99 REFUSED [DO NOT READ]

**[ASK IF USER AND B_D1=1 (YES, USED PROSTHESIS ON DOMINANT SIDE IN PAST 4 WEEKS)] [SP]**

B_D16a. [U_D16a] **Chop vegetables at a counter** with the assistance of your prosthesis. [READ IF NECESSARY: Did you do this or attempt to do this in the past 4 weeks with the assistance of your prosthesis?]

1 YES

2 NO

98 DON’T KNOW/NOT SURE [DO NOT READ]

99 REFUSED [DO NOT READ]

**[ASK IF B_D16a=1 (YES, DID/ATTEMPTED ACTIVITY)] [SP]**

B_D16b. [U_D16b] How easy or difficult was it [READ IF NECESSARY: for you to **chop vegetables at a counter**? Would you say…]

1 Very easy

2 Easy

3 Difficult

4 Very difficult

5 Could not do at all

98 DON’T KNOW/NOT SURE [DO NOT READ]

99 REFUSED [DO NOT READ]

**[ASK IF USER AND B_D1=1 (YES, USED PROSTHESIS ON DOMINANT SIDE IN PAST 4 WEEKS)] [SP]**

B_D17a. [U_D17a] **Spread peanut butter on fresh bread, using your prosthesis**. [READ IF NECESSARY: Did you do this or attempt to do this in the past 4 weeks with the assistance of your prosthesis?]

1 YES

2 NO

98 DON’T KNOW/NOT SURE [DO NOT READ]

99 REFUSED [DO NOT READ]

**[ASK IF B_D17a=1 (YES, DID/ATTEMPTED ACTIVITY)] [SP]**

B_D17b. [U_D17b] How easy or difficult was it [READ IF NECESSARY: for you to **Spread peanut butter on fresh bread, using your prosthesis**? Would you say…]

1 Very easy

2 Easy

3 Difficult

4 Very difficult

5 Could not do at all

98 DON’T KNOW/NOT SURE [DO NOT READ]

99 REFUSED [DO NOT READ]

**[ASK IF USER AND B_D1=1 (YES, USED PROSTHESIS ON DOMINANT SIDE IN PAST 4 WEEKS)] [SP]**

B_D18a. [U_D18a] **Pour from a 12 ounce can with the assistance of your prosthesis without spilling or crushing it**. [READ IF NECESSARY: Did you do this or attempt to do this in the past 4 weeks with the assistance of your prosthesis?]

1 YES

2 NO

98 DON’T KNOW/NOT SURE [DO NOT READ]

99 REFUSED [DO NOT READ]

**[ASK IF B_D18a=1 (YES, DID/ATTEMPTED ACTIVITY)] [SP]**

B_D18b. [U_D18b] How easy or difficult was it [READ IF NECESSARY: for you to **pour from a 12 ounce can without spilling or crushing it**? Would you say…]

1 Very easy

2 Easy

3 Difficult

4 Very difficult

5 Could not do at all

98 DON’T KNOW/NOT SURE [DO NOT READ]

99 REFUSED [DO NOT READ]

**[ASK IF USER AND B_D1=1 (YES, USED PROSTHESIS ON DOMINANT SIDE IN PAST 4 WEEKS)] [SP]**

B_D19a. [U_D19a] **Hold a small bottle to twist off the lid** with the assistance of your prosthesis. [READ IF NECESSARY: Did you do this or attempt to do this in the past 4 weeks with the assistance of your prosthesis?]

1 YES

2 NO

98 DON’T KNOW/NOT SURE [DO NOT READ]

99 REFUSED [DO NOT READ]

**[ASK IF B_D19a=1 (YES, DID/ATTEMPTED ACTIVITY)] [SP]**

B_D19b. [U_D19b] How easy or difficult was it [READ IF NECESSARY: for you to **hold a small bottle to twist off the lid**? Would you say…]

1 Very easy

2 Easy

3 Difficult

4 Very difficult

5 Could not do at all

98 DON’T KNOW/NOT SURE [DO NOT READ]

99 REFUSED [DO NOT READ]

**[ASK IF USER AND B_D1=1 (YES, USED PROSTHESIS ON DOMINANT SIDE IN PAST 4 WEEKS)] [SP]**

B_D20a. [U_D20a] **Hold a bowl to stir** with the assistance of your prosthesis. [READ IF NECESSARY: Did you do this or attempt to do this in the past 4 weeks with the assistance of your prosthesis?]

1 YES

2 NO

98 DON’T KNOW/NOT SURE [DO NOT READ]

99 REFUSED [DO NOT READ]

**[ASK IF B_D20a=1 (YES, DID/ATTEMPTED ACTIVITY)] [SP]**

B_D20b. [U_D20b] How easy or difficult was it [READ IF NECESSARY: for you to **hold a bowl to stir**? Would you say…]

1 Very easy

2 Easy

3 Difficult

4 Very difficult

5 Could not do at all

98 DON’T KNOW/NOT SURE [DO NOT READ]

99 REFUSED [DO NOT READ]

**[ASK IF USER AND B_D1=1 (YES, USED PROSTHESIS ON DOMINANT SIDE IN PAST 4 WEEKS)] [SP]**

B_D21a. [U_D21a] **Hold potatoes or fruit to peel them** with the assistance of your prosthesis. [READ IF NECESSARY: Did you do this or attempt to do this in the past 4 weeks with the assistance of your prosthesis?]

1 YES

2 NO

98 DON’T KNOW/NOT SURE [DO NOT READ]

99 REFUSED [DO NOT READ]

**[ASK IF B_D21a=1 (YES, DID/ATTEMPTED ACTIVITY)] [SP]**

B_D21b. [U_D21b] How easy or difficult was it [READ IF NECESSARY: for you to **hold potatoes or fruit to peel them**? Would you say…]

1 Very easy

2 Easy

3 Difficult

4 Very difficult

5 Could not do at all

98 DON’T KNOW/NOT SURE [DO NOT READ]

99 REFUSED [DO NOT READ]

**[ASK IF USER AND B_D1=1 (YES, USED PROSTHESIS ON DOMINANT SIDE IN PAST 4 WEEKS)] [SP]**

B_D22a. [U_D22a] **Open a bag of chips using both hands** with the assistance of your prosthesis. [READ IF NECESSARY: Did you do this or attempt to do this in the past 4 weeks with the assistance of your prosthesis?]

1 YES

2 NO

98 DON’T KNOW/NOT SURE [DO NOT READ]

99 REFUSED [DO NOT READ]

**[ASK IF B_D22a=1 (YES, DID/ATTEMPTED ACTIVITY)] [SP]**

B_D22b. [U_D22b] How easy or difficult was it [READ IF NECESSARY: for you to **open a bag of chips using both hands**? Would you say…]

1 Very easy

2 Easy

3 Difficult

4 Very difficult

5 Could not do at all

98 DON’T KNOW/NOT SURE [DO NOT READ]

99 REFUSED [DO NOT READ]

**[ASK IF USER AND B_D1=1 (YES, USED PROSTHESIS ON DOMINANT SIDE IN PAST 4 WEEKS)] [SP]**

B_D23a. [U_D23a] **Hold an envelope to open it** with the assistance of your prosthesis. [READ IF NECESSARY: Did you do this or attempt to do this in the past 4 weeks with the assistance of your prosthesis?]

1 YES

2 NO

98 DON’T KNOW/NOT SURE [DO NOT READ]

99 REFUSED [DO NOT READ]

**[ASK IF B_D23a=1 (YES, DID/ATTEMPTED ACTIVITY)] [SP]**

B_D23b. [U_D23b] How easy or difficult was it [READ IF NECESSARY: for you to **hold an envelope to open it**? Would you say…]

1 Very easy

2 Easy

3 Difficult

4 Very difficult

5 Could not do at all

98 DON’T KNOW/NOT SURE [DO NOT READ]

99 REFUSED [DO NOT READ]

**[ASK IF USER AND B_D1=1 (YES, USED PROSTHESIS ON DOMINANT SIDE IN PAST 4 WEEKS)] [SP]**

B_D24a. [U_D24a] **Type on a computer keyboard** with the assistance of your prosthesis. [READ IF NECESSARY: Did you do this or attempt to do this in the past 4 weeks with the assistance of your prosthesis?]

1 YES

2 NO

98 DON’T KNOW/NOT SURE [DO NOT READ]

99 REFUSED [DO NOT READ]

**[ASK IF B_D24a=1 (YES, DID/ATTEMPTED ACTIVITY)] [SP]**

B_D24b. [U_D24b] How easy or difficult was it [READ IF NECESSARY: for you to **type on a computer keyboard**? Would you say…]

1 Very easy

2 Easy

3 Difficult

4 Very difficult

5 Could not do at all

98 DON’T KNOW/NOT SURE [DO NOT READ]

99 REFUSED [DO NOT READ]

**[ASK IF USER AND B_D1=1 (YES, USED PROSTHESIS ON DOMINANT SIDE IN PAST 4 WEEKS)] [SP]**

B_D25a. [U_D25a] **Write your name legibly** with the assistance of your prosthesis. [READ IF NECESSARY: Did you do this or attempt to do this in the past 4 weeks with the assistance of your prosthesis?]

1 YES

2 NO

98 DON’T KNOW/NOT SURE [DO NOT READ]

99 REFUSED [DO NOT READ]

**[ASK IF B_D25a=1 (YES, DID/ATTEMPTED ACTIVITY)] [SP]**

B_D25b. [U_D25b] How easy or difficult was it [READ IF NECESSARY: for you to **write your name legibly**? Would you say…]

1 Very easy

2 Easy

3 Difficult

4 Very difficult

5 Could not do at all

98 DON’T KNOW/NOT SURE [DO NOT READ]

99 REFUSED [DO NOT READ]

**[ASK IF USER AND B_D1=1 (YES, USED PROSTHESIS ON DOMINANT SIDE IN PAST 4 WEEKS)] [SP]**

B_D26a. [U_D26a] **Take a dollar bill out of a wallet** with the assistance of your prosthesis. [READ IF NECESSARY: Did you do this or attempt to do this in the past 4 weeks with the assistance of your prosthesis?]

1 YES

2 NO

98 DON’T KNOW/NOT SURE [DO NOT READ]

99 REFUSED [DO NOT READ]

**[ASK IF B_D26a=1 (YES, DID/ATTEMPTED ACTIVITY)] [SP]**

B_D26b. [U_D26b] How easy or difficult was it [READ IF NECESSARY: for you to **take a dollar bill out of a wallet**? Would you say…]

1 Very easy

2 Easy

3 Difficult

4 Very difficult

5 Could not do at all

98 DON’T KNOW/NOT SURE [DO NOT READ]

99 REFUSED [DO NOT READ]

**[ASK IF USER AND B_D1=1 (YES, USED PROSTHESIS ON DOMINANT SIDE IN PAST 4 WEEKS)] [SP]**

B_D27a. [U_D27a] **Use scissors** with the assistance of your prosthesis. [READ IF NECESSARY: Did you do this or attempt to do this in the past 4 weeks with the assistance of your prosthesis?]

1 YES

2 NO

98 DON’T KNOW/NOT SURE [DO NOT READ]

99 REFUSED [DO NOT READ]

**[ASK IF B_D27a=1 (YES, DID/ATTEMPTED ACTIVITY)] [SP]**

B_D27b. [U_D27b] How easy or difficult was it [READ IF NECESSARY: for you to **use scissors**? Would you say…]

1 Very easy

2 Easy

3 Difficult

4 Very difficult

5 Could not do at all

98 DON’T KNOW/NOT SURE [DO NOT READ]

99 REFUSED [DO NOT READ]

**[ASK IF USER AND B_D1=1 (YES, USED PROSTHESIS ON DOMINANT SIDE IN PAST 4 WEEKS)] [SP]**

B_D28a. [U_D28a] **Use a key in a lock** with the assistance of your prosthesis. [READ IF NECESSARY: Did you do this or attempt to do this in the past 4 weeks with the assistance of your prosthesis?]

1 YES

2 NO

98 DON’T KNOW/NOT SURE [DO NOT READ]

99 REFUSED [DO NOT READ]

**[ASK IF B_D28a=1 (YES, DID/ATTEMPTED ACTIVITY)] [SP]**

B_D28b. [U_D28b] How easy or difficult was it [READ IF NECESSARY: for you to **use a key in a lock**? Would you say…]

1 Very easy

2 Easy

3 Difficult

4 Very difficult

5 Could not do at all

98 DON’T KNOW/NOT SURE [DO NOT READ]

99 REFUSED [DO NOT READ]

**[ASK IF USER AND B_D1=1 (YES, USED PROSTHESIS ON DOMINANT SIDE IN PAST 4 WEEKS)] [SP]**

B_D29a. [U_D29a] **Open a door with a round knob** with the assistance of your prosthesis. [READ IF NECESSARY: Did you do this or attempt to do this in the past 4 weeks with the assistance of your prosthesis?]

1 YES

2 NO

98 DON’T KNOW/NOT SURE [DO NOT READ]

99 REFUSED [DO NOT READ]

**[ASK IF B_D29a=1 (YES, DID/ATTEMPTED ACTIVITY)] [SP]**

B_D29b. [U_D29b] How easy or difficult was it [READ IF NECESSARY: for you to **open a door with a round knob**? Would you say…]

1 Very easy

2 Easy

3 Difficult

4 Very difficult

5 Could not do at all

98 DON’T KNOW/NOT SURE [DO NOT READ]

99 REFUSED [DO NOT READ]

**[ASK IF USER AND B_D1=1 (YES, USED PROSTHESIS ON DOMINANT SIDE IN PAST 4 WEEKS)] [SP]**

B_D30a. [U_D30a] **Hold a nail to hammer** with the assistance of your prosthesis. [READ IF NECESSARY: Did you do this or attempt to do this in the past 4 weeks with the assistance of your prosthesis?]

1 YES

2 NO

98 DON’T KNOW/NOT SURE [DO NOT READ]

99 REFUSED [DO NOT READ]

**[ASK IF B_D30a=1 (YES, DID/ATTEMPTED ACTIVITY)] [SP]**

B_D30b. [U_D30b] How easy or difficult was it [READ IF NECESSARY: for you to **hold a nail to hammer**? Would you say…]

1 Very easy

2 Easy

3 Difficult

4 Very difficult

5 Could not do at all

98 DON’T KNOW/NOT SURE [DO NOT READ]

99 REFUSED [DO NOT READ]

**[ASK IF USER AND B_D1=1 (YES, USED PROSTHESIS ON DOMINANT SIDE IN PAST 4 WEEKS)] [SP]**

B_D31a. [U_D31a] **Carry a laundry basket** with the assistance of your prosthesis. [READ IF NECESSARY: Did you do this or attempt to do this in the past 4 weeks with the assistance of your prosthesis?]

1 YES

2 NO

98 DON’T KNOW/NOT SURE [DO NOT READ]

99 REFUSED [DO NOT READ]

**[ASK IF B_D31a=1 (YES, DID/ATTEMPTED ACTIVITY)] [SP]**

B_D31b. [U_D31b] How easy or difficult was it [READ IF NECESSARY: for you to **carry a laundry basket**? Would you say…]

1 Very easy

2 Easy

3 Difficult

4 Very difficult

5 Could not do at all

98 DON’T KNOW/NOT SURE [DO NOT READ]

99 REFUSED [DO NOT READ]

**[ASK IF USER AND B_D1=1 (YES, USED PROSTHESIS ON DOMINANT SIDE IN PAST 4 WEEKS)] [SP]**

B_D32a. [U_D32a] **Lift a shopping bag containing about 10 pounds, using your prosthesis only**. [READ IF NECESSARY: Did you do this or attempt to do this in the past 4 weeks with the assistance of your prosthesis?]

1 YES

2 NO

98 DON’T KNOW/NOT SURE [DO NOT READ]

99 REFUSED [DO NOT READ]

**[ASK IF B_D32a=1 (YES, DID/ATTEMPTED ACTIVITY)] [SP]**

B_D32b. [U_D32b] How easy or difficult was it [READ IF NECESSARY: for you to **Lift a shopping bag containing about 10 pounds, using your prosthesis only**? Would you say…]

1 Very easy

2 Easy

3 Difficult

4 Very difficult

5 Could not do at all

98 DON’T KNOW/NOT SURE [DO NOT READ]

99 REFUSED [DO NOT READ]

**[ASK IF USER AND B_D1=1 (YES, USED PROSTHESIS ON DOMINANT SIDE IN PAST 4 WEEKS)] [SP]**

B_D33a. [U_D33a] **Lift a shopping bag containing about 20 pounds, using your prosthesis**. [READ IF NECESSARY: Did you do this or attempt to do this in the past 4 weeks with the assistance of your prosthesis?]

1 YES

2 NO

98 DON’T KNOW/NOT SURE [DO NOT READ]

99 REFUSED [DO NOT READ]

**[ASK IF B_D33a=1 (YES, DID/ATTEMPTED ACTIVITY)] [SP]**

B_D33b. [U_D33b] How easy or difficult was it [READ IF NECESSARY: for you to **Lift a shopping bag containing about 20 pounds, using your prosthesis**? Would you say…]

1 Very easy

2 Easy

3 Difficult

4 Very difficult

5 Could not do at all

98 DON’T KNOW/NOT SURE [DO NOT READ]

99 REFUSED [DO NOT READ]

**[ASK IF USER AND B_D1=1 (YES, USED PROSTHESIS ON DOMINANT SIDE IN PAST 4 WEEKS)] [SP]**

B_D34a. [U_D34a] **Lift and carry bulky objects like grocery bags or crates that weigh more than 15 pounds, using your prosthesis**. [READ IF NECESSARY: Did you do this or attempt to do this in the past 4 weeks with the assistance of your prosthesis?]

1 YES

2 NO

98 DON’T KNOW/NOT SURE [DO NOT READ]

99 REFUSED [DO NOT READ]

**[ASK IF B_D34a=1 (YES, DID/ATTEMPTED ACTIVITY)] [SP]**

B_D34b. [U_D34b] How easy or difficult was it [READ IF NECESSARY: for you to **Lift and carry bulky objects like grocery bags or crates that weigh more than 15 pounds, using your prosthesis**? Would you say…]

1 Very easy

2 Easy

3 Difficult

4 Very difficult

5 Could not do at all

98 DON’T KNOW/NOT SURE [DO NOT READ]

99 REFUSED [DO NOT READ]

**Part G: Prosthetic Acceptance**

**[ASK IF USER] [GRID; MP DOWN, SP ACROSS]**

B_G1 [U_G1] Thinking of prostheses, how important are the following to you?

|  | Not at all important | Somewhat important | Very important | DON’T KNOW/NOT SURE/NOT APPLICABLE [DO NOT READ] | REFUSED [DO NOT READ] |
| --- | --- | --- | --- | --- | --- |
| 1. To have a prosthesis that does not restrict the type of clothing you wear [Would you say Not at All Important, Somewhat Important, or Very Important?] | 0 | 1 | 2 | 98 | 99 |
| 1. To have a prosthesis that allows you to wear jewelry on your artificial limb, such as a watch or ring | 0 | 1 | 2 | 98 | 99 |
| 1. To have a prosthesis that looks good with your clothing | 0 | 1 | 2 | 98 | 99 |
| 1. To like the way you look while wearing your prosthesis | 0 | 1 | 2 | 98 | 99 |

**[ASK ALL] [GRID; MP DOWN, SP ACROSS]**

B_G2a [U_G2] To what extent do you disagree or agree with each of the following statements?

|  | Strongly Disagree | Disagree | Neither Disagree nor Agree | Agree | Strongly Agree | DON’T KNOW/ NOT SURE/NOT APPLICABLE [DO NOT READ] | REFUSED [DO NOT READ] |
| --- | --- | --- | --- | --- | --- | --- | --- |
| 1. There are prostheses available that suit my needs [Would you say Strongly Disagree, Disagree, Neither Disagree nor Agree, Agree, or Strongly Agree?] | 1 | 2 | 3 | 4 | 5 | 98 | 99 |
| 1. There are prostheses available that I like | 1 | 2 | 3 | 4 | 5 | 98 | 99 |
| 1. I am afraid that I will hurt someone when wearing a prosthesis | 1 | 2 | 3 | 4 | 5 | 98 | 99 |
| 1. I am afraid that I will scare someone, either a child or an adult, when wearing a prosthesis | 1 | 2 | 3 | 4 | 5 | 98 | 99 |
| 1. I would avoid wearing a prosthesis when caring for a baby | 1 | 2 | 3 | 4 | 5 | 98 | 99 |
| l. I prefer a prosthesis that has a natural-looking hand with fingernails | 1 | 2 | 3 | 4 | 5 | 98 | 99 |
| n. I feel that I have enough information about current prosthetic technologies | 1 | 2 | 3 | 4 | 5 | 98 | 99 |
| o. I **can** get the prosthesis that I really want | 1 | 2 | 3 | 4 | 5 | 98 | 99 |
| p. Some things are just easier to do without a prosthesis | 1 | 2 | 3 | 4 | 5 | 98 | 99 |

**[ASK IF USER OR NON-USER] [GRID; MP DOWN, SP ACROSS]**

B_G2b [U_G2] Thinking of the prosthesis you use or have used on either side, to what extent do you disagree or agree with each of the following statements?

|  | Strongly Disagree | Disagree | Neither Disagree nor Agree | Agree | Strongly Agree | DON’T KNOW/ NOT SURE/NOT APPLICABLE [DO NOT READ] | REFUSED [DO NOT READ] |
| --- | --- | --- | --- | --- | --- | --- | --- |
| **f. [ASK IF USER OR NON_USER]** A prosthesis always works for me | 1 | 2 | 3 | 4 | 5 | 98 | 99 |
| **g. [ASK IF USER OR NON_USER]** Wearing a prosthesis makes my back hurt | 1 | 2 | 3 | 4 | 5 | 98 | 99 |
| **h. [ASK IF USER OR NON_USER]** Wearing a prosthesis makes my neck hurt | 1 | 2 | 3 | 4 | 5 | 98 | 99 |
| **i. [ASK IF USER OR NON_USER]** Wearing a prosthesis makes my stump hurt | 1 | 2 | 3 | 4 | 5 | 98 | 99 |
| **j. [ASK IF USER OR NON_USER]** Wearing a prosthesis makes my stump uncomfortable | 1 | 2 | 3 | 4 | 5 | 98 | 99 |
| **k. [ASK IF USER]** I am satisfied with the function of the wrist of my prosthesis | 1 | 2 | 3 | 4 | 5 | 98 | 99 |
| m. **[ASK IF USER OR NON_USER]** I am more likely to wear a prosthesis now than when I was younger | 1 | 2 | 3 | 4 | 5 | 98 | 99 |

**[ASK ALL] [GRID; MP DOWN, SP ACROSS]**

B_G3 [U_G3]

**[ASK IF USER]** Thinking about either your left or right side, in general, how often do you do each of the following?

**[ASK IF NON_USER OR NEVER_USER]** Thinking about either your left or right side, in general, how often do you…

|  | Never | Rarely | Occasionally | Regularly | Always | DON’T KNOW/ NOT SURE [DO NOT READ] | REFUSED [DO NOT READ] |
| --- | --- | --- | --- | --- | --- | --- | --- |
| 1. **[ASK IF USER]** Avoid wearing a prosthesis because you do not like the fit [Would you say Never, Rarely, Occasionally, Regularly, or Always?] | 0 | 1 | 2 | 3 | 4 | 98 | 99 |
| 1. **[ASK IF USER]** Avoid wearing a prosthesis because it does not fit **under** your clothes. | 0 | 1 | 2 | 3 | 4 | 98 | 99 |
| 1. **[ASK IF USER]** Avoid wearing a prosthesis because of the way it fits **with** your clothes. | 0 | 1 | 2 | 3 | 4 | 98 | 99 |
| 1. Use assistive devices or adaptive equipment, like a button hook, or special kitchen tool, to help you do everyday tasks | 0 | 1 | 2 | 3 | 4 | 98 | 99 |

**[ASK IF USER] [SP]**

B_G4. [U_G4] Do you pay any out-of-pocket expenses related to your prostheses?

1 YES

2 NO

98 DON’T KNOW/NOT SURE [DO NOT READ]

99 REFUSED [DO NOT READ]

**[ASK IF NON_USER] [SP]**

B_G4a. [U_G4a] Have you ever paid any out-of-pocket expenses related to a prosthesis?

1 YES

2 NO

98 DON’T KNOW/NOT SURE [DO NOT READ]

99 REFUSED [DO NOT READ]

**[ASK IF B_G4=1 OR B_G4a =1 (YES, PAY OUT-OF-POCKET EXPENSES)] [GRID; MP DOWN, SP ACROSS]**

B_G5 [U_G5] To what extent do you disagree or agree with each of the following statements?

|  | Strongly Disagree | Disagree | Neither Disagree nor Agree | Agree | Strongly Agree | DON’T KNOW/ NOT SURE [DO NOT READ] | REFUSED [DO NOT READ] |
| --- | --- | --- | --- | --- | --- | --- | --- |
| 1. I can afford the out-of-pocket expenses to purchase a prosthesis [Would you say Strongly Disagree, Disagree, Neither Disagree nor Agree, Agree, or Strongly Agree?] | 1 | 2 | 3 | 4 | 5 | 98 | 99 |
| 1. I can afford the out-of-pocket expenses to maintain a prosthesis | 1 | 2 | 3 | 4 | 5 | 98 | 99 |
| 1. I can afford the out-of-pocket expenses to repair a prosthesis as soon as needed | 1 | 2 | 3 | 4 | 5 | 98 | 99 |
| 1. I can afford the out-of-pocket expenses to replace a prosthesis as soon as needed | 1 | 2 | 3 | 4 | 5 | 98 | 99 |

**Part N: Questions about You**

**[ASK ALL] [SP]**

B_N1. [U_N1] What is your current military status?

1 Veteran

2 Civilian

3 Active Duty

98 DON’T KNOW/NOT SURE [DO NOT READ]

99 REFUSED [DO NOT READ]

**[ASK ALL] [MP]**

B_N4. [U_N4] What is your race? I will read you a list of choices. You may choose one or more.

**[**CHECK ALL THAT APPLY**]**

1 White or Caucasian

2 Black or African-American

3 Asian

4 American Indian or Alaska Native

5 Native Hawaiian or Pacific Islander

6 Other [Please explain]

98 DON’T KNOW/NOT SURE [DO NOT READ]

99 REFUSED [DO NOT READ]

**[ASK IF B_N4=6 (OTHER)] [TEXT BOX]**

B_N4_SPECIFY. __________________________________
