# Supplementary material for: Prosthesis usability experience is associated with extent of upper limb prosthesis adoption: A Structural Equation Modeling (SEM) analysis
Source: PLoS One. 2024 Jun 25;19(6):e0299155. doi: 10.1371/journal.pone.0299155 (PMC11198835; doi:10.1371/journal.pone.0299155)
Supplement: S1 Data — (ZIP) [file pone.0299155.s011.zip › List of Code and Metadata files .docx]

**Code and Metadata files uploaded to the Brown University Digital Repository**

1. [Prosthesis usability experience: Study Protocol](https://repository.library.brown.edu/studio/item/bdr:jsav4qgs/)  <https://repository.library.brown.edu/studio/item/bdr:jsav4qgs/>
2. [Data collection instrument: Screener and Survey](https://repository.library.brown.edu/studio/item/bdr:7f78hvmu/)  https://repository.library.brown.edu/studio/item/bdr:7f78hvmu/
3. [Codebook for Structural Equation Model](https://repository.library.brown.edu/studio/item/bdr:udb6b9e9/)  https://repository.library.brown.edu/studio/item/bdr:udb6b9e9/
4. [MPLUS syntax and output for Structural Equation Model](https://repository.library.brown.edu/studio/item/bdr:jzu827p7/)  https://repository.library.brown.edu/studio/item/bdr:jzu827p7/
