# Supplementary material for: Prosthesis usability experience is associated with extent of upper limb prosthesis adoption: A Structural Equation Modeling (SEM) analysis
Source: PLoS One. 2024 Jun 25;19(6):e0299155. doi: 10.1371/journal.pone.0299155 (PMC11198835; doi:10.1371/journal.pone.0299155)
Supplement: S1 Data — (ZIP) [file pone.0299155.s011.zip › Mplus Syntax and Output of Structural Equation Model.rtf]

Syntax and Output from Mplus Structural Equation Models

Mplus VERSION 8.10
MUTHEN & MUTHEN
10/10/2023  12:29 PM

INPUT INSTRUCTIONS

  TITLE:      SEM mediation moderate,
              interaction effect;
  DATA:       FILE = data.dat;
              TYPE = INDIVIDUAL;
  VARIABLE:   NAMES =
  hours_use
  days_use
  age
  male
  black
  race_uk
  race_gt1
  SH
  TH
  bilateral
  single_dof
  multi_dof
  cosmetic
  appearance
  trust
  desirability
  comfort
  ease_use
  onehand
  twohand
  ;

              USEVARIABLES =

  appearance
  trust
  desirability
  comfort
  ease_use

  hours_use
  days_use
  onehand
  twohand


  age
  male
  black race_uk race_gt1
  SH TH bilateral
  single_dof multi_dof cosmetic
  ;

  categorical=
  hours_use
  days_use
  ;

              MISSING = ALL(999);

  ANALYSIS:   TYPE = RANDOM;

  MODEL:
  experience by
  appearance *
  trust
  desirability
  comfort
  ease_use
  ;

  experience@1;

  adoption by
  hours_use *
  days_use
  onehand
  twohand
  ;

  adoption@1;

  onehand with twohand ;

  s_SH | experience xwith SH ;
  s_TH | experience xwith TH ;
  s_bilateral | experience xwith bilateral ;

  s_single_dof | experience xwith single_dof ;
  s_multi_dof | experience xwith multi_dof ;
  s_cosmetic | experience xwith cosmetic ;

  adoption on
  experience (b1)
  age
  male
  black
  race_uk
  race_gt1
  SH (c11)
  TH (c12)
  bilateral  (c13)
  single_dof (c21)
  multi_dof  (c22)
  cosmetic   (c23)

  s_SH        (b11)
  s_TH        (b12)
  s_bilateral (b13)

  s_single_dof (b21)
  s_multi_dof  (b22)
  s_cosmetic   (b23)
  ;


  experience on
  SH (a11)
  TH (a12)
  bilateral (a13)
  single_dof (a21)
  multi_dof (a22)
  cosmetic (a23)
  ;


  MODEL CONSTRAINT:
  new(
  cid11_21, cid11_22, cid11_23,
  cid12_21, cid12_22, cid12_23,
  cid13_21, cid13_22, cid13_23,

  cid21_11, cid21_12, cid21_13,
  cid22_11, cid22_12, cid22_13,
  cid23_11, cid23_12, cid23_13
  );

  cid11_21=a11*(b1+b11+b21); ! SH throught single_dof to adoption
  cid11_22=a11*(b1+b11+b22); ! SH throught multi_dof to adoption
  cid11_23=a11*(b1+b11+b23); ! SH throught cosmetic to adoption

  cid12_21=a12*(b1+b12+b21); ! TH throught single_dof to adoption
  cid12_22=a12*(b1+b12+b22); ! TH throught multi_dof to adoption
  cid12_23=a12*(b1+b12+b23); ! TH throught cosmetic to adoption

  cid13_21=a13*(b1+b13+b21); ! bilateral throught single_dof to adoption
  cid13_22=a13*(b1+b13+b22); ! bilateral throught multi_dof to adoption
  cid13_23=a13*(b1+b13+b23); ! bilateral throught cosmetic to adoption

  cid21_11=a21*(b1+b21+b11); ! single_dof throught SH to adoption
  cid21_12=a21*(b1+b21+b12); ! single_dof throught TH to adoption
  cid21_13=a21*(b1+b21+b13); ! single_dof throught bilateral to adoption

  cid22_11=a22*(b1+b22+b11); ! multi_dof throught SH to adoption
  cid22_12=a22*(b1+b22+b12); ! multi_dof throught TH to adoption
  cid22_13=a22*(b1+b22+b13); ! multi_dof throught bilateral to adoption

  cid23_11=a23*(b1+b23+b11); ! cosmetic throught SH to adoption
  cid23_12=a23*(b1+b23+b12); ! cosmetic throught TH to adoption
  cid23_13=a23*(b1+b23+b13); ! cosmetic throught bilateral to adoption


  output:  sampstat  standardized;


*** WARNING in VARIABLE command
  Note that only the first 8 characters of variable names are used in the output.
  Shorten variable names to avoid any confusion.
   1 WARNING(S) FOUND IN THE INPUT INSTRUCTIONS


SEM mediation moderate,
interaction effect;

SUMMARY OF ANALYSIS

Number of groups                                                 1
Number of observations                                         402

Number of dependent variables                                    9
Number of independent variables                                 11
Number of continuous latent variables                            8

Observed dependent variables

  Continuous
   APPEARANCE  TRUST       DESIRABILI  COMFORT     EASE_USE    ONEHAND
   TWOHAND

  Binary and ordered categorical (ordinal)
   HOURS_US    DAYS_USE

Observed independent variables
   AGE         MALE        BLACK       RACE_UK     RACE_GT1    SH
   TH          BILATERA    SINGLE_D    MULTI_DO    COSMETIC

Continuous latent variables
   EXPERIEN    ADOPTION    S_SH        S_TH        S_BILATE    S_SINGLE
   S_MULTI_    S_COSMET


Estimator                                                      MLR
Information matrix                                        OBSERVED
Optimization Specifications for the Quasi-Newton Algorithm for
Continuous Outcomes
  Maximum number of iterations                                 100
  Convergence criterion                                  0.100D-05
Optimization Specifications for the EM Algorithm
  Maximum number of iterations                                 500
  Convergence criteria
    Loglikelihood change                                 0.100D-02
    Relative loglikelihood change                        0.100D-05
    Derivative                                           0.100D-02
Optimization Specifications for the M step of the EM Algorithm for
Categorical Latent variables
  Number of M step iterations                                    1
  M step convergence criterion                           0.100D-02
  Basis for M step termination                           ITERATION
Optimization Specifications for the M step of the EM Algorithm for
Censored, Binary or Ordered Categorical (Ordinal), Unordered
Categorical (Nominal) and Count Outcomes
  Number of M step iterations                                    1
  M step convergence criterion                           0.100D-02
  Basis for M step termination                           ITERATION
  Maximum value for logit thresholds                            15
  Minimum value for logit thresholds                           -15
  Minimum expected cell size for chi-square              0.100D-01
Maximum number of iterations for H1                           2000
Convergence criterion for H1                             0.100D-03
Optimization algorithm                                         EMA
Integration Specifications
  Type                                                    STANDARD
  Number of integration points                                  15
  Dimensions of numerical integration                            2
  Adaptive quadrature                                           ON
Link                                                         LOGIT
Cholesky                                                       OFF

Input data file(s)
  data.dat
Input data format  FREE


SUMMARY OF DATA

     Number of missing data patterns             1
     Number of y missing data patterns           1
     Number of u missing data patterns           1


COVARIANCE COVERAGE OF DATA

Minimum covariance coverage value   0.100


     PROPORTION OF DATA PRESENT FOR Y


           Covariance Coverage
              APPEARAN      TRUST         DESIRABI      COMFORT       EASE_USE
              ________      ________      ________      ________      ________
 APPEARAN       1.000
 TRUST          1.000         1.000
 DESIRABI       1.000         1.000         1.000
 COMFORT        1.000         1.000         1.000         1.000
 EASE_USE       1.000         1.000         1.000         1.000         1.000
 ONEHAND        1.000         1.000         1.000         1.000         1.000
 TWOHAND        1.000         1.000         1.000         1.000         1.000
 AGE            1.000         1.000         1.000         1.000         1.000
 MALE           1.000         1.000         1.000         1.000         1.000
 BLACK          1.000         1.000         1.000         1.000         1.000
 RACE_UK        1.000         1.000         1.000         1.000         1.000
 RACE_GT1       1.000         1.000         1.000         1.000         1.000
 SH             1.000         1.000         1.000         1.000         1.000
 TH             1.000         1.000         1.000         1.000         1.000
 BILATERA       1.000         1.000         1.000         1.000         1.000
 SINGLE_D       1.000         1.000         1.000         1.000         1.000
 MULTI_DO       1.000         1.000         1.000         1.000         1.000
 COSMETIC       1.000         1.000         1.000         1.000         1.000


           Covariance Coverage
              ONEHAND       TWOHAND       AGE           MALE          BLACK
              ________      ________      ________      ________      ________
 ONEHAND        1.000
 TWOHAND        1.000         1.000
 AGE            1.000         1.000         1.000
 MALE           1.000         1.000         1.000         1.000
 BLACK          1.000         1.000         1.000         1.000         1.000
 RACE_UK        1.000         1.000         1.000         1.000         1.000
 RACE_GT1       1.000         1.000         1.000         1.000         1.000
 SH             1.000         1.000         1.000         1.000         1.000
 TH             1.000         1.000         1.000         1.000         1.000
 BILATERA       1.000         1.000         1.000         1.000         1.000
 SINGLE_D       1.000         1.000         1.000         1.000         1.000
 MULTI_DO       1.000         1.000         1.000         1.000         1.000
 COSMETIC       1.000         1.000         1.000         1.000         1.000


           Covariance Coverage
              RACE_UK       RACE_GT1      SH            TH            BILATERA
              ________      ________      ________      ________      ________
 RACE_UK        1.000
 RACE_GT1       1.000         1.000
 SH             1.000         1.000         1.000
 TH             1.000         1.000         1.000         1.000
 BILATERA       1.000         1.000         1.000         1.000         1.000
 SINGLE_D       1.000         1.000         1.000         1.000         1.000
 MULTI_DO       1.000         1.000         1.000         1.000         1.000
 COSMETIC       1.000         1.000         1.000         1.000         1.000


           Covariance Coverage
              SINGLE_D      MULTI_DO      COSMETIC
              ________      ________      ________
 SINGLE_D       1.000
 MULTI_DO       1.000         1.000
 COSMETIC       1.000         1.000         1.000


UNIVARIATE PROPORTIONS AND COUNTS FOR CATEGORICAL VARIABLES

    HOURS_US
      Category 1    0.132           53.000
      Category 2    0.109           44.000
      Category 3    0.164           66.000
      Category 4    0.249          100.000
      Category 5    0.346          139.000
    DAYS_USE
      Category 1    0.007            3.000
      Category 2    0.025           10.000
      Category 3    0.037           15.000
      Category 4    0.187           75.000
      Category 5    0.744          299.000


SAMPLE STATISTICS


     ESTIMATED SAMPLE STATISTICS


           Means
              APPEARAN      TRUST         DESIRABI      COMFORT       EASE_USE
              ________      ________      ________      ________      ________
                2.191        -0.101         0.859         0.956         1.354


           Means
              ONEHAND       TWOHAND       AGE           MALE          BLACK
              ________      ________      ________      ________      ________
               -1.677        -1.996        61.714         0.803         0.080


           Means
              RACE_UK       RACE_GT1      SH            TH            BILATERA
              ________      ________      ________      ________      ________
                0.042         0.035         0.060         0.199         0.080


           Means
              SINGLE_D      MULTI_DO      COSMETIC
              ________      ________      ________
                0.149         0.112         0.057


           Covariances
              APPEARAN      TRUST         DESIRABI      COMFORT       EASE_USE
              ________      ________      ________      ________      ________
 APPEARAN       4.933
 TRUST          1.309         4.097
 DESIRABI       1.187         0.724         2.379
 COMFORT        1.869         1.940         1.281         6.035
 EASE_USE       1.408         0.913         0.867         1.352         2.674
 ONEHAND        0.520         0.285         0.595         0.470         0.329
 TWOHAND        1.040         0.504         0.981         0.934         0.602
 AGE            2.588        -0.140         1.807         2.484         3.193
 MALE           0.075        -0.021         0.110         0.043         0.057
 BLACK         -0.054         0.026        -0.020         0.033        -0.052
 RACE_UK        0.013         0.032        -0.007        -0.018        -0.019
 RACE_GT1      -0.031        -0.017        -0.003         0.000        -0.002
 SH            -0.024         0.008        -0.013        -0.019        -0.034
 TH            -0.001         0.019        -0.016        -0.033        -0.060
 BILATERA      -0.022        -0.018        -0.017        -0.074        -0.039
 SINGLE_D       0.007         0.050         0.018         0.094         0.027
 MULTI_DO      -0.071         0.019        -0.034         0.060        -0.038
 COSMETIC      -0.018         0.067        -0.017        -0.011         0.022


           Covariances
              ONEHAND       TWOHAND       AGE           MALE          BLACK
              ________      ________      ________      ________      ________
 ONEHAND        3.486
 TWOHAND        1.992         3.058
 AGE            0.269        -0.786       207.488
 MALE          -0.036        -0.057         1.737         0.158
 BLACK         -0.047        -0.072        -0.159         0.008         0.073
 RACE_UK       -0.026        -0.022        -0.279         0.006        -0.003
 RACE_GT1       0.004        -0.011        -0.107        -0.001        -0.003
 SH            -0.084        -0.067        -0.142        -0.001        -0.002
 TH            -0.195        -0.152        -0.376         0.009         0.007
 BILATERA       0.245         0.010        -0.022        -0.014        -0.004
 SINGLE_D       0.081         0.063        -0.694        -0.030         0.001
 MULTI_DO      -0.023        -0.033        -1.204        -0.018         0.001
 COSMETIC      -0.061        -0.073        -0.155        -0.021         0.005


           Covariances
              RACE_UK       RACE_GT1      SH            TH            BILATERA
              ________      ________      ________      ________      ________
 RACE_UK        0.041
 RACE_GT1      -0.001         0.034
 SH             0.005        -0.002         0.056
 TH             0.007         0.006        -0.012         0.159
 BILATERA       0.002        -0.003        -0.005        -0.016         0.073
 SINGLE_D      -0.006        -0.003        -0.001        -0.017         0.001
 MULTI_DO       0.003         0.004         0.003        -0.007         0.001
 COSMETIC       0.000        -0.002         0.007        -0.001        -0.002


           Covariances
              SINGLE_D      MULTI_DO      COSMETIC
              ________      ________      ________
 SINGLE_D       0.127
 MULTI_DO      -0.017         0.099
 COSMETIC      -0.009        -0.006         0.054


           Correlations
              APPEARAN      TRUST         DESIRABI      COMFORT       EASE_USE
              ________      ________      ________      ________      ________
 APPEARAN       1.000
 TRUST          0.291         1.000
 DESIRABI       0.346         0.232         1.000
 COMFORT        0.342         0.390         0.338         1.000
 EASE_USE       0.388         0.276         0.344         0.336         1.000
 ONEHAND        0.125         0.076         0.207         0.102         0.108
 TWOHAND        0.268         0.142         0.364         0.217         0.211
 AGE            0.081        -0.005         0.081         0.070         0.136
 MALE           0.085        -0.026         0.179         0.044         0.088
 BLACK         -0.090         0.048        -0.047         0.050        -0.118
 RACE_UK        0.029         0.079        -0.021        -0.036        -0.059
 RACE_GT1      -0.077        -0.046        -0.010        -0.001        -0.008
 SH            -0.046         0.017        -0.037        -0.033        -0.088
 TH            -0.001         0.023        -0.026        -0.034        -0.091
 BILATERA      -0.037        -0.033        -0.041        -0.111        -0.087
 SINGLE_D       0.009         0.069         0.034         0.108         0.046
 MULTI_DO      -0.101         0.030        -0.070         0.078        -0.074
 COSMETIC      -0.035         0.142        -0.049        -0.019         0.057


           Correlations
              ONEHAND       TWOHAND       AGE           MALE          BLACK
              ________      ________      ________      ________      ________
 ONEHAND        1.000
 TWOHAND        0.610         1.000
 AGE            0.010        -0.031         1.000
 MALE          -0.048        -0.083         0.304         1.000
 BLACK         -0.092        -0.153        -0.041         0.076         1.000
 RACE_UK       -0.070        -0.063        -0.096         0.073        -0.062
 RACE_GT1       0.013        -0.036        -0.040        -0.008        -0.056
 SH            -0.191        -0.162        -0.042        -0.007        -0.035
 TH            -0.262        -0.218        -0.065         0.058         0.061
 BILATERA       0.485         0.021        -0.006        -0.132        -0.053
 SINGLE_D       0.122         0.100        -0.135        -0.214         0.006
 MULTI_DO      -0.039        -0.060        -0.265        -0.142         0.012
 COSMETIC      -0.141        -0.179        -0.046        -0.229         0.086


           Correlations
              RACE_UK       RACE_GT1      SH            TH            BILATERA
              ________      ________      ________      ________      ________
 RACE_UK        1.000
 RACE_GT1      -0.040         1.000
 SH             0.104        -0.048         1.000
 TH             0.081         0.075        -0.126         1.000
 BILATERA       0.030        -0.056        -0.074        -0.147         1.000
 SINGLE_D      -0.088        -0.041        -0.017        -0.121         0.006
 MULTI_DO       0.043         0.062         0.044        -0.058         0.012
 COSMETIC       0.001        -0.047         0.119        -0.015        -0.033


           Correlations
              SINGLE_D      MULTI_DO      COSMETIC
              ________      ________      ________
 SINGLE_D       1.000
 MULTI_DO      -0.149         1.000
 COSMETIC      -0.103        -0.087         1.000


     MAXIMUM LOG-LIKELIHOOD VALUE FOR THE UNRESTRICTED (H1) MODEL IS -7389.618


UNIVARIATE SAMPLE STATISTICS


     UNIVARIATE HIGHER-ORDER MOMENT DESCRIPTIVE STATISTICS

         Variable/         Mean/     Skewness/   Minimum/ % with                Percentiles
        Sample Size      Variance    Kurtosis    Maximum  Min/Max      20%/60%    40%/80%    Median

     APPEARANCE            2.191      -0.667      -4.450    0.75%       0.200      1.490      2.660
             402.000       4.933      -0.552       4.370   19.40%       4.220      4.220
     TRUST                -0.101      -0.147      -5.830    2.49%      -1.790     -0.790      0.120
             402.000       4.097       0.947       4.690    4.23%       0.120      1.050
     DESIRABILITY          0.859       0.288      -5.130    0.25%      -0.450      0.500      0.770
             402.000       2.379       1.209       5.730    0.75%       1.210      1.840
     COMFORT               0.956       0.279      -6.210    1.00%      -1.110      0.750      0.750
             402.000       6.035       0.770       6.650    6.72%       1.820      1.820
     EASE_USE              1.354       0.128      -3.950    0.50%       0.020      0.760      1.160
             402.000       2.674      -0.237       4.210   13.68%       1.610      2.950
     ONEHAND              -1.677       0.817      -4.200    0.25%      -3.730     -2.350     -2.350
             402.000       3.486       0.262       4.290    0.75%      -1.390     -0.130
     TWOHAND              -1.996      -0.222      -5.890    0.25%      -3.270     -2.130     -1.720
             402.000       3.058       0.823       4.700    0.25%      -1.350     -0.670
     AGE                  61.714      -0.708      18.000    0.25%      49.000     62.000     65.000
             402.000     207.488      -0.072      95.000    0.25%      69.000     73.000
     MALE                  0.803      -1.527       0.000   19.65%       1.000      1.000      1.000
             402.000       0.158       0.333       1.000   80.35%       1.000      1.000
     BLACK                 0.080       3.106       0.000   92.04%       0.000      0.000      0.000
             402.000       0.073       7.649       1.000    7.96%       0.000      0.000
     RACE_UK               0.042       4.549       0.000   95.77%       0.000      0.000      0.000
             402.000       0.041      18.691       1.000    4.23%       0.000      0.000
     RACE_GT1              0.035       5.074       0.000   96.52%       0.000      0.000      0.000
             402.000       0.034      23.750       1.000    3.48%       0.000      0.000
     SH                    0.060       3.717       0.000   94.03%       0.000      0.000      0.000
             402.000       0.056      11.813       1.000    5.97%       0.000      0.000
     TH                    0.199       1.508       0.000   80.10%       0.000      0.000      0.000
             402.000       0.159       0.273       1.000   19.90%       0.000      0.000
     BILATERAL             0.080       3.106       0.000   92.04%       0.000      0.000      0.000
             402.000       0.073       7.649       1.000    7.96%       0.000      0.000
     SINGLE_DOF            0.149       1.969       0.000   85.07%       0.000      0.000      0.000
             402.000       0.127       1.875       1.000   14.93%       0.000      0.000
     MULTI_DOF             0.112       2.462       0.000   88.81%       0.000      0.000      0.000
             402.000       0.099       4.059       1.000   11.19%       0.000      0.000
     COSMETIC              0.057       3.813       0.000   94.28%       0.000      0.000      0.000
             402.000       0.054      12.539       1.000    5.72%       0.000      0.000


THE MODEL ESTIMATION TERMINATED NORMALLY

  ERROR OCCURRED IN THE BRANT WALD TEST FOR PROPORTIONAL ODDS FOR DAYS_USE.


MODEL FIT INFORMATION

Number of Free Parameters                       56

Loglikelihood

          H0 Value                       -6249.639
          H0 Scaling Correction Factor      1.0541
            for MLR

Information Criteria

          Akaike (AIC)                   12611.278
          Bayesian (BIC)                 12835.079
          Sample-Size Adjusted BIC       12657.386
            (n* = (n + 2) / 24)


MODEL RESULTS

                                                    Two-Tailed
                    Estimate       S.E.  Est./S.E.    P-Value

 EXPERIEN BY
    APPEARANCE         1.397      0.111     12.639      0.000
    TRUST              0.977      0.127      7.699      0.000
    DESIRABILI         0.862      0.082     10.499      0.000
    COMFORT            1.384      0.158      8.749      0.000
    EASE_USE           0.939      0.089     10.506      0.000

 ADOPTION BY
    HOURS_USE          2.199      0.439      5.013      0.000
    DAYS_USE           3.000      0.804      3.731      0.000
    ONEHAND            0.678      0.108      6.254      0.000
    TWOHAND            0.747      0.084      8.910      0.000

 ADOPTION   ON
    EXPERIENCE         0.906      0.201      4.511      0.000
    S_SH              -0.201      0.215     -0.934      0.350
    S_TH              -0.408      0.214     -1.907      0.057
    S_BILATERA         0.153      0.930      0.164      0.870
    S_SINGLE_D         0.221      0.197      1.123      0.261
    S_MULTI_DO        -0.467      0.272     -1.717      0.086
    S_COSMETIC        -0.483      0.204     -2.361      0.018

 ADOPTION   ON
    AGE               -0.007      0.005     -1.409      0.159
    MALE              -0.166      0.173     -0.962      0.336
    BLACK             -0.004      0.188     -0.022      0.982
    RACE_UK            0.380      0.242      1.571      0.116
    RACE_GT1          -0.512      0.432     -1.185      0.236
    SH                -1.009      0.262     -3.851      0.000
    TH                -0.827      0.203     -4.082      0.000
    BILATERAL          1.325      1.024      1.294      0.196
    SINGLE_DOF        -0.215      0.211     -1.019      0.308
    MULTI_DOF         -0.687      0.233     -2.945      0.003
    COSMETIC          -0.336      0.225     -1.493      0.135

 EXPERIENCE ON
    SH                -0.398      0.251     -1.586      0.113
    TH                -0.186      0.150     -1.240      0.215
    BILATERAL         -0.449      0.194     -2.321      0.020
    SINGLE_DOF         0.194      0.204      0.952      0.341
    MULTI_DOF         -0.165      0.228     -0.724      0.469
    COSMETIC           0.109      0.233      0.468      0.640

 ONEHAND  WITH
    TWOHAND            0.961      0.195      4.920      0.000

 Intercepts
    APPEARANCE         2.303      0.144     15.977      0.000
    TRUST             -0.023      0.121     -0.194      0.846
    DESIRABILI         0.927      0.093      9.980      0.000
    COMFORT            1.066      0.153      6.966      0.000
    EASE_USE           1.429      0.105     13.625      0.000
    ONEHAND           -1.089      0.250     -4.355      0.000
    TWOHAND           -1.349      0.294     -4.581      0.000

 Thresholds
    HOURS_US$1        -5.890      1.219     -4.832      0.000
    HOURS_US$2        -4.459      1.100     -4.052      0.000
    HOURS_US$3        -2.881      0.976     -2.952      0.003
    HOURS_US$4        -0.588      0.847     -0.694      0.488
    DAYS_USE$1       -13.127      2.810     -4.672      0.000
    DAYS_USE$2       -10.816      2.418     -4.473      0.000
    DAYS_USE$3        -9.317      2.247     -4.146      0.000
    DAYS_USE$4        -5.720      1.646     -3.475      0.001

 Residual Variances
    APPEARANCE         2.919      0.275     10.624      0.000
    TRUST              3.110      0.295     10.528      0.000
    DESIRABILI         1.612      0.161     10.006      0.000
    COMFORT            4.057      0.445      9.111      0.000
    EASE_USE           1.764      0.149     11.876      0.000
    ONEHAND            2.550      0.296      8.604      0.000
    TWOHAND            1.922      0.177     10.861      0.000
    EXPERIENCE         1.000      0.000    999.000    999.000
    ADOPTION           1.000      0.000    999.000    999.000

New/Additional Parameters
    CID11_21          -0.368      0.248     -1.482      0.138
    CID11_22          -0.094      0.113     -0.832      0.405
    CID11_23          -0.088      0.104     -0.851      0.395
    CID12_21          -0.133      0.108     -1.237      0.216
    CID12_22          -0.006      0.055     -0.103      0.918
    CID12_23          -0.003      0.041     -0.068      0.946
    CID13_21          -0.575      0.508     -1.131      0.258
    CID13_22          -0.266      0.413     -0.644      0.520
    CID13_23          -0.259      0.426     -0.607      0.544
    CID21_11           0.180      0.200      0.898      0.369
    CID21_12           0.139      0.160      0.872      0.383
    CID21_13           0.248      0.268      0.927      0.354
    CID22_11          -0.039      0.063     -0.616      0.538
    CID22_12          -0.005      0.049     -0.102      0.919
    CID22_13          -0.097      0.233     -0.418      0.676
    CID23_11           0.024      0.058      0.414      0.679
    CID23_12           0.002      0.025      0.066      0.947
    CID23_13           0.063      0.157      0.400      0.689


QUALITY OF NUMERICAL RESULTS

     Condition Number for the Information Matrix              0.703E-05
       (ratio of smallest to largest eigenvalue)


BRANT WALD TEST FOR PROPORTIONAL ODDS

                                   Degrees of
                      Chi-Square     Freedom   P-Value

  HOURS_USE
    Overall test         122.354        33      0.000
    AGE                    2.265         3      0.519
    MALE                   1.821         3      0.610
    BLACK                  3.625         3      0.305
    RACE_UK                1.189         3      0.756
    RACE_GT1               2.749         3      0.432
    SH                     1.134         3      0.769
    TH                     2.218         3      0.528
    BILATERAL              2.187         3      0.534
    SINGLE_DOF             1.569         3      0.666
    MULTI_DOF              8.437         3      0.038
    COSMETIC              14.940         3      0.002


STANDARDIZED MODEL RESULTS


STDYX Standardization

                                                    Two-Tailed
                    Estimate       S.E.  Est./S.E.    P-Value

 EXPERIEN BY
    APPEARANCE         0.639      0.041     15.420      0.000
    TRUST              0.491      0.054      9.038      0.000
    DESIRABILI         0.568      0.043     13.298      0.000
    COMFORT            0.573      0.056     10.263      0.000
    EASE_USE           0.584      0.046     12.674      0.000

 ADOPTION BY
    HOURS_USE          0.867      0.034     25.465      0.000
    DAYS_USE           0.922      0.035     25.987      0.000
    ONEHAND            0.521      0.098      5.333      0.000
    TWOHAND            0.612      0.045     13.697      0.000

 ADOPTION   ON
    EXPERIENCE         0.641      0.116      5.537      0.000
    S_SH              -0.034      0.035     -0.954      0.340
    S_TH              -0.115      0.057     -2.022      0.043
    S_BILATERA         0.029      0.176      0.166      0.868
    S_SINGLE_D         0.056      0.051      1.093      0.274
    S_MULTI_DO        -0.104      0.056     -1.868      0.062
    S_COSMETIC        -0.079      0.030     -2.614      0.009

 ADOPTION   ON
    AGE               -0.072      0.053     -1.367      0.172
    MALE              -0.046      0.048     -0.968      0.333
    BLACK             -0.001      0.035     -0.022      0.982
    RACE_UK            0.053      0.034      1.573      0.116
    RACE_GT1          -0.065      0.056     -1.165      0.244
    SH                -0.166      0.041     -4.019      0.000
    TH                -0.230      0.051     -4.488      0.000
    BILATERAL          0.250      0.174      1.438      0.150
    SINGLE_DOF        -0.053      0.054     -0.990      0.322
    MULTI_DOF         -0.151      0.052     -2.873      0.004
    COSMETIC          -0.054      0.035     -1.533      0.125

 EXPERIENCE ON
    SH                -0.093      0.058     -1.598      0.110
    TH                -0.073      0.059     -1.245      0.213
    BILATERAL         -0.120      0.051     -2.348      0.019
    SINGLE_DOF         0.068      0.071      0.956      0.339
    MULTI_DOF         -0.051      0.071     -0.725      0.468
    COSMETIC           0.025      0.053      0.468      0.640

 ONEHAND  WITH
    TWOHAND            0.434      0.061      7.072      0.000

 Intercepts
    APPEARANCE         1.037      0.081     12.721      0.000
    TRUST             -0.012      0.060     -0.194      0.846
    DESIRABILI         0.601      0.060      9.979      0.000
    COMFORT            0.434      0.062      6.951      0.000
    EASE_USE           0.874      0.067     12.997      0.000
    ONEHAND           -0.582      0.141     -4.126      0.000
    TWOHAND           -0.769      0.170     -4.520      0.000

 Thresholds
    HOURS_US$1        -1.617      0.262     -6.182      0.000
    HOURS_US$2        -1.224      0.250     -4.893      0.000
    HOURS_US$3        -0.791      0.239     -3.315      0.001
    HOURS_US$4        -0.161      0.228     -0.706      0.480
    DAYS_USE$1        -2.808      0.357     -7.867      0.000
    DAYS_USE$2        -2.313      0.302     -7.653      0.000
    DAYS_USE$3        -1.993      0.290     -6.873      0.000
    DAYS_USE$4        -1.223      0.257     -4.769      0.000

 Residual Variances
    APPEARANCE         0.591      0.053     11.164      0.000
    TRUST              0.759      0.053     14.237      0.000
    DESIRABILI         0.678      0.048     13.977      0.000
    COMFORT            0.672      0.064     10.514      0.000
    EASE_USE           0.659      0.054     12.265      0.000
    ONEHAND            0.729      0.102      7.171      0.000
    TWOHAND            0.625      0.055     11.434      0.000
    EXPERIENCE         0.968      0.021     46.649      0.000
    ADOPTION           0.485      0.105      4.628      0.000


STDY Standardization

                                                    Two-Tailed
                    Estimate       S.E.  Est./S.E.    P-Value

 EXPERIEN BY
    APPEARANCE         0.639      0.041     15.420      0.000
    TRUST              0.491      0.054      9.038      0.000
    DESIRABILI         0.568      0.043     13.298      0.000
    COMFORT            0.573      0.056     10.263      0.000
    EASE_USE           0.584      0.046     12.674      0.000

 ADOPTION BY
    HOURS_USE          0.867      0.034     25.465      0.000
    DAYS_USE           0.922      0.035     25.987      0.000
    ONEHAND            0.521      0.098      5.333      0.000
    TWOHAND            0.612      0.045     13.697      0.000

 ADOPTION   ON
    EXPERIENCE         0.641      0.116      5.537      0.000
    S_SH              -0.142      0.149     -0.955      0.340
    S_TH              -0.289      0.142     -2.026      0.043
    S_BILATERA         0.108      0.650      0.166      0.868
    S_SINGLE_D         0.156      0.143      1.094      0.274
    S_MULTI_DO        -0.331      0.177     -1.872      0.061
    S_COSMETIC        -0.342      0.130     -2.625      0.009

 ADOPTION   ON
    AGE               -0.005      0.004     -1.368      0.171
    MALE              -0.116      0.120     -0.969      0.333
    BLACK             -0.003      0.131     -0.022      0.982
    RACE_UK            0.265      0.168      1.575      0.115
    RACE_GT1          -0.356      0.306     -1.166      0.243
    SH                -0.702      0.173     -4.056      0.000
    TH                -0.576      0.127     -4.537      0.000
    BILATERAL          0.922      0.641      1.440      0.150
    SINGLE_DOF        -0.149      0.151     -0.991      0.322
    MULTI_DOF         -0.478      0.166     -2.887      0.004
    COSMETIC          -0.234      0.152     -1.535      0.125

 EXPERIENCE ON
    SH                -0.391      0.244     -1.601      0.109
    TH                -0.183      0.147     -1.247      0.213
    BILATERAL         -0.442      0.188     -2.356      0.018
    SINGLE_DOF         0.191      0.200      0.956      0.339
    MULTI_DOF         -0.162      0.224     -0.726      0.468
    COSMETIC           0.107      0.229      0.468      0.640

 ONEHAND  WITH
    TWOHAND            0.434      0.061      7.072      0.000

 Intercepts
    APPEARANCE         1.037      0.081     12.721      0.000
    TRUST             -0.012      0.060     -0.194      0.846
    DESIRABILI         0.601      0.060      9.979      0.000
    COMFORT            0.434      0.062      6.951      0.000
    EASE_USE           0.874      0.067     12.997      0.000
    ONEHAND           -0.582      0.141     -4.126      0.000
    TWOHAND           -0.769      0.170     -4.520      0.000

 Thresholds
    HOURS_US$1        -1.617      0.262     -6.182      0.000
    HOURS_US$2        -1.224      0.250     -4.893      0.000
    HOURS_US$3        -0.791      0.239     -3.315      0.001
    HOURS_US$4        -0.161      0.228     -0.706      0.480
    DAYS_USE$1        -2.808      0.357     -7.867      0.000
    DAYS_USE$2        -2.313      0.302     -7.653      0.000
    DAYS_USE$3        -1.993      0.290     -6.873      0.000
    DAYS_USE$4        -1.223      0.257     -4.769      0.000

 Residual Variances
    APPEARANCE         0.591      0.053     11.164      0.000
    TRUST              0.759      0.053     14.237      0.000
    DESIRABILI         0.678      0.048     13.977      0.000
    COMFORT            0.672      0.064     10.514      0.000
    EASE_USE           0.659      0.054     12.265      0.000
    ONEHAND            0.729      0.102      7.171      0.000
    TWOHAND            0.625      0.055     11.434      0.000
    EXPERIENCE         0.968      0.021     46.649      0.000
    ADOPTION           0.485      0.105      4.628      0.000


STD Standardization

                                                    Two-Tailed
                    Estimate       S.E.  Est./S.E.    P-Value

 EXPERIEN BY
    APPEARANCE         1.420      0.111     12.799      0.000
    TRUST              0.994      0.128      7.753      0.000
    DESIRABILI         0.876      0.084     10.381      0.000
    COMFORT            1.407      0.162      8.702      0.000
    EASE_USE           0.955      0.092     10.407      0.000

 ADOPTION BY
    HOURS_USE          3.159      0.500      6.314      0.000
    DAYS_USE           4.309      1.102      3.911      0.000
    ONEHAND            0.974      0.207      4.715      0.000
    TWOHAND            1.073      0.110      9.799      0.000

 ADOPTION   ON
    EXPERIENCE         0.641      0.116      5.537      0.000
    S_SH              -0.142      0.149     -0.955      0.340
    S_TH              -0.289      0.142     -2.026      0.043
    S_BILATERA         0.108      0.650      0.166      0.868
    S_SINGLE_D         0.156      0.143      1.094      0.274
    S_MULTI_DO        -0.331      0.177     -1.872      0.061
    S_COSMETIC        -0.342      0.130     -2.625      0.009

 ADOPTION   ON
    AGE               -0.005      0.004     -1.368      0.171
    MALE              -0.116      0.120     -0.969      0.333
    BLACK             -0.003      0.131     -0.022      0.982
    RACE_UK            0.265      0.168      1.575      0.115
    RACE_GT1          -0.356      0.306     -1.166      0.243
    SH                -0.702      0.173     -4.056      0.000
    TH                -0.576      0.127     -4.537      0.000
    BILATERAL          0.922      0.641      1.440      0.150
    SINGLE_DOF        -0.149      0.151     -0.991      0.322
    MULTI_DOF         -0.478      0.166     -2.887      0.004
    COSMETIC          -0.234      0.152     -1.535      0.125

 EXPERIENCE ON
    SH                -0.391      0.244     -1.601      0.109
    TH                -0.183      0.147     -1.247      0.213
    BILATERAL         -0.442      0.188     -2.356      0.018
    SINGLE_DOF         0.191      0.200      0.956      0.339
    MULTI_DOF         -0.162      0.224     -0.726      0.468
    COSMETIC           0.107      0.229      0.468      0.640

 ONEHAND  WITH
    TWOHAND            0.961      0.195      4.920      0.000

 Intercepts
    APPEARANCE         2.303      0.144     15.977      0.000
    TRUST             -0.023      0.121     -0.194      0.846
    DESIRABILI         0.927      0.093      9.980      0.000
    COMFORT            1.066      0.153      6.966      0.000
    EASE_USE           1.429      0.105     13.625      0.000
    ONEHAND           -1.089      0.250     -4.355      0.000
    TWOHAND           -1.349      0.294     -4.581      0.000

 Thresholds
    HOURS_US$1        -5.890      1.219     -4.832      0.000
    HOURS_US$2        -4.459      1.100     -4.052      0.000
    HOURS_US$3        -2.881      0.976     -2.952      0.003
    HOURS_US$4        -0.588      0.847     -0.694      0.488
    DAYS_USE$1       -13.127      2.810     -4.672      0.000
    DAYS_USE$2       -10.816      2.418     -4.473      0.000
    DAYS_USE$3        -9.317      2.247     -4.146      0.000
    DAYS_USE$4        -5.720      1.646     -3.475      0.001

 Residual Variances
    APPEARANCE         2.919      0.275     10.624      0.000
    TRUST              3.110      0.295     10.528      0.000
    DESIRABILI         1.612      0.161     10.006      0.000
    COMFORT            4.057      0.445      9.111      0.000
    EASE_USE           1.764      0.149     11.876      0.000
    ONEHAND            2.550      0.296      8.604      0.000
    TWOHAND            1.922      0.177     10.861      0.000
    EXPERIENCE         0.968      0.021     46.649      0.000
    ADOPTION           0.485      0.105      4.628      0.000


R-SQUARE

    Observed                                        Two-Tailed
    Variable        Estimate       S.E.  Est./S.E.    P-Value

    HOURS_US           0.752      0.059     12.733      0.000
    DAYS_USE           0.850      0.065     12.994      0.000
    APPEARAN           0.409      0.053      7.710      0.000
    TRUST              0.241      0.053      4.519      0.000
    DESIRABI           0.322      0.048      6.649      0.000
    COMFORT            0.328      0.064      5.132      0.000
    EASE_USE           0.341      0.054      6.337      0.000
    ONEHAND            0.271      0.102      2.667      0.008
    TWOHAND            0.375      0.055      6.848      0.000

     Latent                                         Two-Tailed
    Variable        Estimate       S.E.  Est./S.E.    P-Value

    EXPERIEN           0.032      0.021      1.555      0.120
    ADOPTION           0.515      0.105      4.921      0.000


DIAGRAM INFORMATION

  Use View Diagram under the Diagram menu in the Mplus Editor to view the diagram.
  If running Mplus from the Mplus Diagrammer, the diagram opens automatically.

  Diagram output
    c:\ni\linda resnik\tams_final_data\partf\sem10052022\draft_manuscript_on_sem_predicting_extent_of_prosthesis_use\sem

     Beginning Time:  12:29:38
        Ending Time:  12:29:56
       Elapsed Time:  00:00:18


MUTHEN & MUTHEN
3463 Stoner Ave.
Los Angeles, CA  90066

Tel: (310) 391-9971
Fax: (310) 391-8971
Web: www.StatModel.com
Support: Support@StatModel.com

Copyright (c) 1998-2023 Muthen & Muthen
