# Supplementary material for: Prosthesis usability experience is associated with extent of upper limb prosthesis adoption: A Structural Equation Modeling (SEM) analysis
Source: PLoS One. 2024 Jun 25;19(6):e0299155. doi: 10.1371/journal.pone.0299155 (PMC11198835; doi:10.1371/journal.pone.0299155)
Supplement: S1 Data — (ZIP) [file pone.0299155.s011.zip › Prosthesis usability experience_Study Protocol.doc]

**Study Protocol**

The protocol for the study reported in the manuscript, “Prosthesis usability experience is associated with extent of upper limb prosthesis adoption: A structural equation modeling analysis,” was approved as the fourth aim (Aim 4) of a larger, multi-study protocol approved by the Central IRB of the Department of Veterans Affairs titled, Needs, Preferences and Functional Abilities of Veterans and Service Members with Upper Limb Amputation. Only the portions of the larger protocol that are relevant to this manuscript are provided below.

Principal Investigator/Study Chair: Linda Resnik, PT, PhD

Initial Approval for Aim 4 : 7/5/2018

Modification for Aim 4 May 1, 2020

**Abstract**

**BACKGROUND** Quality gaps in care of military and Veterans with upper limb amputation have been reported. In 2008, amputees receiving prosthetic care in the VA were reported to be less satisfied than counterparts receiving care in the private sector. In 2011, reported widespread dissatisfaction amongst combat Veterans with upper limb loss led to calls for efforts to evaluate needs of Veterans with traumatic upper limb amputations to improve satisfaction. Major efforts to improve quality of prosthetic care have been made since these studies were conducted. In 2009, the VA reorganized its amputation system of care, and in 2014 the VA and DoD released the Evidence-Based Clinical Practice Guidelines (CPGs) for the rehabilitation of persons with upper limb amputation. It is now time for a comprehensive study to assess the current state of quality and outcomes of amputation rehabilitation for upper limb amputees and to track quality and outcomes over time.

**OBJECTIVES** Our objective is to provide comprehensive cross-sectional and longitudinal data on function, needs, preferences, and satisfaction of Veterans and service members with major upper limb amputation.

**SPECIFIC AIMS**

1) Describe patterns of prosthesis use; identify the impact of amputation and prosthesis use on function, activities and participation; and identify unmet prosthetic needs.
2) Conduct a one year longitudinal follow-up survey to examine changes in satisfaction with care and prosthetic services, physical performance, self-reported quality of life and physical function to assess the implementation of new clinical practice guidelines (CPGs).
3) Quantify physical function using a battery of performance based tests.
4) Develop and validate patient-reported outcome measures for persons with upper limb amputation.
5) Compare the effectiveness of prosthesis type and components, and quantify the impact of prosthesis form (e.g. weight, shape) on outcomes.

**STUDY DESIGN** This is a 5-part study. Part 1 will survey a stratified random sample of 1834 persons with upper limb amputation that receive care in the VA and all service members with upper limb amputation who have not transitioned to VA care. This N represents ~44% of the population. We will stratify by level of amputation, oversample the smallest groups, and use sampling weights in analyses. The survey will include items from prior surveys, standardized measures (SF-12, QuickDASH, TAPES satisfaction, and OPUS care satisfaction) and new items on risk-benefit assessment and elements of quality. Part 2 is a one year longitudinal follow-up survey of 667 respondents from Part 1. Part 3 will involve 5 data collection sites that will administer measures of dexterity and activity performance to 125 persons at two time points one year apart. Part 4 will develop, refine and validate patient reported outcome measures for persons with upper limb amputation. Part 5 will involved 5 data collection sites that will administer measures of dexterity and activity performance to 200 persons during 1 study visit. Data from Part 5 will be pooled with data from Part 3 to achieve Aim 5.

**List of Abbreviations**

| AAOP | American Association of Orthotics & Prosthetics |
| --- | --- |
| AE | Adverse Event |
| AM-ULA | Activities Measure for Upper Limb Amputation |
| ANOVAs | Analysis of Variance |
| ATO | Authorization to Operate |
| BAMC | Brooke Army Medical Center |
| CAG | Citrix Access Gateway |
| CCTV | Closed-circuit television |
| CDMRP | Congressionally Directed Medical Research Program |
| CDW | Corporate Data Warehouse |
| CFI | Center for the Intrepid |
| CPG | Clinical Practice Guidelines |
| CSD | Client Satisfaction with device scale (OPUS) |
| CTD | Cumulative Trauma Disorders |
| DARPA | Defense Advanced Research Projects Agency |
| DART | Data Access Request Tracker |
| DASH | Disabilities of the Arm and Shoulder (DASH) |
| DIF | Differential Item Functioning |
| DoD | Department of Defense |
| EACE | DoD-VA Extremity Trauma & Amputation Center of Excellence |
| EMED | Expeditionary Medical Encounter Database |
| EMG | Electromyography |
| FDA | Food & Drug Administration |
| FISMA | Federal Information Security Management Act |
| GVAMC | Gainesville VA Medical Center |
| HIPAA | Health Insurance Portability and Accountability Act of 1996 |
| HJF | Henry Jackson Foundation for the Advancement of Military Medicine, Inc. |
| HRPO | Human Research Protection Office (DoD) |
| ICC | Intraclass correlation coefficient |
| ISENS | Implanted Somatosensory Electrical Neurostimulation and Sensing System |
| ISR Burn Center  MCS | United States Army Institute of Surgical Research Burn CenterMental Component Summary (VR-12) |
| MDR | Military Health System Data Repository |
| NCI | National Cancer Institute |
| NDS | National Data Systems |
| NIST | National Institute of Standards and Technology |
| NPPD | VA National Prosthetics Patient Database |
| OEF | Operation Enduring Freedom |
| OI&T | VA Office of Information and Technology |
| OIF | Operation Iraqi Freedom |
| OIG | Office of the Inspector General |
| OPORP | Orthotics and Prosthetics Outcomes Research Program |
| OPUS | Orthotics and Prosthetics Users Survey |
| OPUS-CSD-R | Client Satisfaction with device scale (OPUS), modified |
| ORP | Office of Research Protections (DoD) |
| OSR | Office of Survey Research (at UMMS) |
| OSRI | Ocean State Research Institute |
| P-UEFS | Prosthesis Upper Extremity Functional Scale (OPUS) |
| PCS | Physical Component Summary (VR-12) |
| PEM | Patient Experience Measure |
| PEQ | Prosthetics Evaluation Questionnaire |
| PI | Principal Investigator |
| PSHS | VA Puget Sound Healthcare System |
| PVAMC | Providence VA Medical Center |
| PROMS | Patient Reported Outcome Measure |
| QuickDASH | Disabilities of the Arm, Shoulder and Hand Score |
| RVAMC | Richmond VA Medical Center |
| SD | Should Disarticulation |
| SF-12 | The Short Form 12-item Survey |
| SF-36 | 36-Item Short Form Health Survey |
| SRS | Sensory Restoration System |
| TAIHOD | Total Army Injury Health Outcomes Database |
| TAPES | Trinity Prosthetic Experience Scale |
| TAPES-MOD | Trinity Amputation and Prosthetic Experience Scale, Modified |
| TAPES-UPPER | Trinity Amputation and Prosthetic Experience Scale, Modified for upper limb amputees |
| TH | Transhumeral |
| TIRR | TIRR Memorial Hermann |
| TR | Transradial |
| UEFS | Upper Extremity Functional Scale (OPUS) |
| UMMS | University of Massachusetts Medical School |
| USAMRMC | The US Army Medical Research and Materiel Command (Fort Detrick) |
| USF | University of South Florida |
| VA | Department of Veterans Affairs |
| VA CIRB | Veterans Administration Central Institutional Review Board |
| VA MedSAS | VA Medical Statistical Analysis System Database |
| VA RR&D | Veterans Administration Rehabilitation Research and Development |
| VINCI | VA Informatics and Computing Infrastructure |
| VPN | Virtual Private Network |
| VR-12 | Veterans Rand 12-item Health Survey |
| WOC | Without Compensation (VA Appointment) |

**Protocol Title:** **Needs, Preferences and Functional Abilities of Veterans and Service Members with Upper Limb Amputation**

Objectives (Aim 4 only)

**4) Develop and validate patient-reported outcome measures for persons with upper limb amputation.**

4a. Refine the Trinity Amputation and Prosthetic Experience Scale (TAPES) Satisfaction subscale and the Orthotics Prosthetics Users Survey (OPUS) Client Satisfaction with Device (CSD) scales to incorporate issues of concern to women, and then evaluate reliability, validity, and gender-based scoring.

4b. Develop and evaluate reliability, validity, and gender-based scoring of a modified version of the OPUS Upper Extremity Functional Scale, the P-UEFS, which measures perceived difficulty in activity performance when using an upper limb prosthesis.

4c. Refine and test the validity of a new Patient Experience Measure (PEM) designed to capture the impact of a sensory restoration system (SRS).

4d. Assess the reliability, validity and gender based scoring of the Prosthetics Evaluation Questionnaire (PEQ) utility and residual limb health subscales.

Aim 4 is a measure development initiative and thus the major hypotheses relate to validation and are described in the data analysis section.

# 1.0 Resources and Personnel

Site: Providence VA Medical Center:

**Linda Resnik, PT, PhD** Principal Investigator (Contributed RR&D Research Career Scientist) - Dr. Linda Resnik is a VA RR&D Career Scientist who focuses on rehabilitation outcomes and health services research. Dr. Resnik will oversee all scientific aspects of the project, and conduct of study activities, direct the staff training, and disseminate study related findings. Dr. Resnik will have access to protected health information and will perform data analysis.

**Sarah Ekerholm, MPH,** Project Manager

Ms. Ekerholm will be the main contact for PVAMC and will maintain regular communication with the investigative teams at all sites. She will be responsible for day-to-day project coordination and management of all study activities at the PVAMC. Ms. Ekerholm will supervise the research assistant and Statistician/Programmer at PVAMC. She will be responsible for planning work to be accomplished, assigning work, setting and adjusting short-term priorities, and preparing schedules for completion of work. She will work closely with Dr. Clark to coordinate the mailings and operations for study Parts 1 and 2, and with Drs. Resnik and Highsmith to develop training materials for study Part 3. She will travel as needed to support staff training and pre-implementation activities. She will provide oversight and quality assurance monitoring for all collaborating sites. She will serve as a resource for the Research Assistants/Study Coordinators at each of the Part 3 sites, audit data, and meet with RAs regularly by telephone during the recruitment and data collection phase to ensure they are following study procedures. Ms. Ekerholm will establish the database for primary data collection for Part 3 of the study and will serve as the data manager. She will perform periodic audits of all data entry completed the by research assistant. Ms. Ekerholm will also receive data from the University of Massachusetts, Center for the Intrepid (CFI) and Institute of Surgical Research (ISR) Burn Center teams. Ms. Ekerholm will have access to protected health information.

**Matthew Borgia, AM,** Statistical Programmer/Biostatistician

Mr. Borgia will serve as a statistical programmer/biostatistician at the PVAMC. In Year 1 he will request a VA VINCI workspace for the study data, complete the required Data Access Request Tracker Application (DART) request (once IRB approval is received). He will then use VA data to identify the sampling frame. Mr. Borgia will also receive the list of SSNs from the DOD to check against the Veteran list and identify unique persons. Mr. Borgia will work with Drs. Resnik and Clark to calculate the sampling fraction and make random selection of persons with amputation from within each target strata. In Year 1 he will conduct preliminary analyses of Part 1 surveys. In Years 2-3, under the direction of Dr. Resnik, he will conduct preliminary and final analyses to address each of the study aims and hypotheses. Mr. Borgia will participate in regular meetings with Dr. Resnik throughout all years of the project and in weekly meetings with the entire analytic team during years 2 and 3. Mr. Borgia will run reports and provide regular updates to Dr. Resnik, and will be responsible for preparing data according to the Data Sharing Plan. Mr. Borgia will have access to protected health information and will perform data analysis.

Site: University of Massachusetts Medical School

**Melissa Clark, Ph.D.** Site Principal Investigator
Dr. Clark is Senior Director of the Research and Evaluation Unit, Center for Health Policy and Research and Professor of Quantitative Health Sciences, University of Massachusetts Medical School. She is a Survey Methodologist with extensive experience in questionnaire development, data collection methods, and psychometric analyses with a focus on underserved and vulnerable populations.

Dr. Clark has led data collection efforts for a number of large scale surveys. For instance, Dr. Clark served as Principal Investigator of the Measures, Methods, and Quality Control Core of the renewal of a National Cancer Institute funded program project [NCI funded P-50 SPORE - Transdisciplinary Tobacco Use Research Center]. She was also the Principal Investigator of the Primary Data Collection Core of “Shaping Long Term Care in America”, a Program Project Grant sponsored by the National Institute on Aging. She also led the data collection activities for a study assessing the long-term quality of life experiences of breast cancer survivors. Prior to that, she was the Principal Investigator of a NCI-funded grant that evaluated three survey protocols (mailed questionnaire, telephone interview, and computerized self-administered questionnaire) for collecting cancer-related data from women ages 40 to 75 years. She was also the Principal Investigator of a project funded by Komen for the Cure to study the cancer-screening related experiences of women with disabilities. As PI or Study Director of each of these large, multidisciplinary projects, she has directed the development of all study protocols and has been responsible for leading all data collection activities. Dr. Clark will be responsible for all administrative and scientific activities of the project related to the University of Massachusetts Medical School subcontract. Dr. Clark and her staff will have access to identifiable data.

# 2.0 Study Procedures

## Study Design

**The study** will involve cognitive interviewing, pilot testing of new and refined measures, with measure administration through telephone and paper and pencil surveys**.**

Cognitive interviewing will be conducted in a sample of approximately 20 persons. Findings from cognitive interviewing will be used to refine the new measures which will then be pilot tested with approximately 20 persons. 50 persons will participate in reliability testing. Calibration data from the telephone administered surveys will be collected and used for Rasch modeling. One hundred persons in the calibration sample will also participate in the mode of administration testing and will complete the survey twice, half randomly selected to complete by phone first, and the other half by paper and pencil first.

**Risks/Benefits Assessment:**
We consider the risks of this Aim of this study to be minimal. However, there is some risk of loss of confidentiality in sharing personal experiences or opinions and having performance evaluated through surveys and interviews.

All risks are considered low and safeguards are listed below.

s
**Risk management and emergency response:**
1) Risk of loss of confidentiality: Participants must verbally consent (over the telephone) indicating their willingness to participate in the study, their willingness to be videotaped or audio taped/recorded, their understanding of risks and benefits and their understanding that participating is voluntary. Unless required by law, only the study investigators, members of the investigator’s staff and the IRB will have authority to review study records. To protect confidentiality, each subject will be given a unique identification number that will be used in the name field of all study related paperwork. The code for the study numbers will be kept separate from the data and will not contain any personal information. All paper data regarding subjects (will be kept confidential and stored in a secure location (locked filing cabinets) at each participating study site. Study materials will be stored in accordance with VA Record Control Schedule 10.1 and may also be used for scientific presentation.

All electronic copies of study files will be maintained on the VAMC secure research server. All video recordings of study sessions recorded via handheld video camera (Aims 3 and 5) will be transferred using one of the following secure methods: a) uploaded to the secure study SharePoint, b) saved directly into Dr. Resnik’s secure research folder, or c) burned onto a CD and encrypted or loaded onto an encrypted drive before mailing by secure carrier to the PVAMC research team. After videos have been uploaded and saved in Dr. Linda Resnik’s secure research folder on the Providence VAMC secure server, the local site will be notified, after which they will be immediately deleted from the recording device and/or SharePoint. OI&T staff is responsible for all electronic data back-up.

UMMS Survey Center: Once received, participant information will be processed within the secure environment of the University of Massachusetts Medical School (UMMS) Office of Survey Research. After completion of all telephone surveys, and midway through data collection for each aim, analytic datasets will be created without identifiers and sent to PVAMC via approved VA methods – CAG, encrypted thumb drive/external hard drive, SAFE (Safe Access File Exchange), encrypted CD and/or encrypted email. Note: All survey center staff working on this project will have VA Without Compensation Appointments (WOC).

A deidentified dataset will be shared with our collaborators at Boston University to assist with planned analyses. We will share this data by email or SAFE (Safe Access File Exchange).

**Potential benefits:**

There are no direct benefits to participants for taking part in this research study. However, the information we get from this study might help to treat future patients with upper limb amputation.

Minorities will not be discriminated against in the recruitment of subjects for this study. Although the population of Veteran and service member upper limb amputees is predominantly Caucasian males, we will make every attempt to include minority subjects in the study. All female major upper limb amputees will be recruited.

## Recruitment Methods

Several methods will be used to recruit participants as described below:

1. Participants will be recruited from Aims 2 and 3 completers of the overall parent study. Only those agreed to be contacted for additional study activities will be mailed recruitment packages containing the following documents
   a. Invitation letter
   b. Information sheet

Participants will have the opportunity to opt out of Aim 4 participation by calling the survey center, or by opting out when the survey center reaches the participant.

2. We will work with VINCI to modify our currently approved DART and identify any Veterans who have received care at the VA between Jauary 1 2016 – June 1 2019 with a diagnosis of major upper limb amputation. The sample will be identified from Corporate Data Warehouse (CDW) sources including VA MedSAS and the VHA Benefits file. We will remove all persons who previously participated in our study, those who indicated they did not want to participate in our early study aims and also those who indicated they were ineligible because they did not have an upper limb amputation. We will mail recruitment packages containing an invitation letter, an information sheet explaining the study and a post card with a subject number to return if they wish to opt out of participation. Participants will have the opportunity to opt out by mailing in the card, contacting the PVAMC study team, contacting the survey center, or notifying survey center team when they call the participant to complete the survey. If we have not received an opt-out card within 30 days, the University of Massachusetts survey center team will begin calling potential participants. If a person does not want to take part in our study, they will have the opportunity to opt-out when reached by the survey center team.
3. Amputee Coalition of America: Participants will be recruited through the Amputee Coalition of America using the following methods:
   a. Email: Recruitment emails (e-blast) will be distributed to all persons with upper limb loss or limb difference within the Amputee Coalition of America database who have signed up for their e-blast email service. The email will contain information about study activities. Those who are interested in participating or finding out more will be able to provide their contact information to the Amputee Coalition who will then transfer this information, (using secure methods) to the PVAMC study team. Interested parties may also call the PVAMC study team directly, or contact the UMMS team directly by phone or web based link. All email recruitment materials will be submitted to the CIRB for approval. We will include a study information sheet in the e-blast, and if participants request hard copies we will mail an information sheet and letter.
   b. Social media: The Amputee Coalition will post information about the study and a link or phone number to call if interested on their website, facebook and twitter. All website, facebook and twitter language will be submitted to the CIRB for approval.
   c. We may also contact amputee support group leaders through the Amputee Coalition website and ask that they share recruitment documents at their support group meetings (support groups can be virtual or in-person). If a person is interested in being part of the study, they would call the Local Site study staff to learn more about the study. The support group leaders will not make any referrals, or share any contact information with study staff.
4. **Hanger Clinics:** We will partner with Hanger Clinics, a company that provides prosthetic and orthotic care to both Veterans and non-Veterans with upper limb amputation in communities across the US, to assist with recruitment. We will share IRB-approved recruitment materials (study poster and flier) with contacts at Hanger and Hanger staff will share recruitment materials and discuss the study at clinic visits, support groups, etc. Hanger staff may also use their clinic database to identify potential participants, and send a mailing with IRB-approved recruitment materials. Potential participants who receive study information from Hanger staff or from a Hanger mailing can call the PVAMC study staff at the University of Massachusetts Survey Center if they are interested in learning more about the study. Study staff will screen potential participants, and if eligible and interested, the Survey Center team will then complete the phone survey. Hanger providers will not make any referrals, and will not provide any contact information to the PVAMC study team. Hanger activities will be approved by the Research Review Committee at Hanger.
5. We may partner with non-VA community based colleagues (PTs, OTs, physicians, nurses, prosthetists, etc), amputee support groups, peer mentors, or other resource groups to identify potential participants. The non-VA community-based providers may share our recruitment materials at a clinic visit, in the waiting room or exam room, through a mailing, etc. If a potential participant learns of the study through a non-VA provider, the potential participant will call the PVAMC or UMass research staff listed on the recruitment materials for more information. The non-VA providers and support groups will not make any referrals and will not share any patient information with the PVAMC or UMass study teams. Interested persons will be mailed a study information sheet and letter.

**Incentives for Participation**
Veterans and Service members will receive remuneration for completing each part of the study

$20 per study activity

Payment will be sent in the form of a gift card and will be mailed upon confirmation that a study activity has been completed.

## Informed Consent Procedures

The new Veterans identified using our modified DART, and all participants from earlier study Aims who have not opted out of participation in future study activities will be sent a recruitment package containing an Aim 4 invitation letter and information sheet, and an opt out card for those who were not a part of Aims 2 or 3. These participants will have the opportunity to opt out of participation by calling the UMMS Survey Center or the PVAMC study team, or mailing in the opt out card if it was part of their package.

Participants recruited through the Amputation Coalition of America will receive an email with study information and a link or phone number to call for additional information, and a study information sheet. If they are not interested in the study, they will not call the phone number or click the link. Participants who are interested will provide their contact information to the ACA, UMSS or the PVAMC research team.

Participants recruited through non-VA community based providers will opt-in to participation, and if they do not want to participate they will not call the PVAMC or UMass study teams. If they call the teams and then decide they do not want to participate, they can opt-out at that time. They will also be mailed a study information sheet.

All participants (regardless of recruitment method) who did not opt out of participation will have the study explained to them by the University of Massachusetts Survey Center staff. They will have the opportunity to ask questions, and may decline participation at this time. We have a Waiver of Documentation of Informed Consent in place for the survey portion of this study and are amending it to include Aim 4, so Consent will not be formally documented for Aim 4.

##  Inclusion/Exclusion Criteria

**Inclusion Criteria**

- Persons who have completed Aim 2 or 3 of the parent study and consented to be contacted for additional study activities
- Persons who are identified through our new data pull and are non-deceased with a diagnosis of major upper limb amputation (forequarter, shoulder disarticulation, transhumeral, elbow disarticulation, transradial, or wrist disarticulation level) and are able to understand study requirements. They must be able to hear well enough to comprehend questions administered over the telephone.
- Persons (Veterans or non-Veterans) who are recruited directly into Aim 4 using other approved recruitment methods.

Participants who are recruited directly into the study (and not identified through VA or DoD data sources) must meet the following criteria:

- Must have a major upper limb amputation (forequarter, shoulder disarticulation, transhumeral, elbow disarticulation, transradial, or wrist disarticulation level)
- Must be able to hear well enough to comprehend questions administered over the telephone

**Exclusion Criteria**

- Only amputation of fingers or hand
- Difficulty with hearing over a telephone that creates inability to comprehend questions administered over the telephone (Excluded from telephone survey only, may be eligible for paper and pencil survey)
- Major cognitive impairment that would limit the ability to participate in a phone survey This will be assessed by the study interviewer while talking to a participant, or by family member.

## 5.5 Study Evaluations

**Screening**

The telephone surveyor will ask the participant a few questions about their amputation to ensure that inclusion/exclusion criteria are met.

**Study Survey:**

The metrics collected include:

- Modified version of the TAPES Satisfaction Scale (TAPES-MOD)
- Modified OPUS Client Satisfaction with Device (CSD)-R
- Modified OPUS Prosthesis Upper Extremity Functional Scale (P-UEFS)
- Modified PEQ-Utility and Residual Limb Health subscales
- QuickDASH
- SF-12
- Refined Patient Experience Measure (PEM)
- Amputation, demographic and prosthetic use questions from Aim 1 survey
- PROMIS Upper Extremity 7 item Short Form and additional items
- Prosthesis Acceptance Measure

**Modified Version TAPES Satisfaction subscale (TAPES-MOD)** Our preliminary, cognitive and pilot testing will develop a modified version of the TAPES prosthetic satisfaction scale. Although the TAPES’ content and scoring were developed originally for lower limb amputees,^70,71^ its structural validity was examined and scoring modified for use with upper limb amputees (TAPES-Upper).^51^ While the TAPES for lower limb amputees has 3 satisfaction related scales, the TAPES-Upper has only 1 satisfaction scale with evidence supporting its structural validity for use in upper limb amputees. Nevertheless, the appropriateness of TAPES scoring has never been examined in women. In fact only 1 of the 101 participants in the study validating the TAPES in upper limb amputees was female, leading the authors to recommend further research to investigate the replicability of the factor structure for women.^51^ This research has not been conducted. Thus, it is not clear if the TAPES captures elements of satisfaction that are important to women.

**Modified OPUS Client Satisfaction with Device Scale (CSD-R)** Our cognitive and pilot testing will result in a modification to the OPUS Client Satisfaction with Device Scale (CSD), one of five instruments of the Orthotics and Prosthetics User Survey.^52^ The OPUS CSD, like the other OPUS instruments, was developed using contemporary measurement methods. It contains 11 items relating to prosthesis weight, ease of donning, durability, fit, appearance, comfort, wear and tear from clothes, pain of wearing, skin abrasions, cost of maintenance and cost of repair. Respondents indicate their extent of agreement with statements about these aspects of their prosthesis using a 4 point scale (strongly agree, agree, disagree, strongly disagree). Nine of the items are summed to achieve the final score recorded (cost items are excluded).

In Aim 1 of this study, we added questions to the OPUS CSD about 1) appropriateness of the size of the terminal device, 2) appropriateness of size of the prosthesis, 3) importance of having a prosthesis that does not restrict choice of clothing, and 4) importance of prosthesis that allows wearing jewelry on artificial limb.. Analysis of the OPUS CSD data from our Aim 1 will provide an opportunity for us to modify the measure, to create the OPUS CSD-R.

**Modified OPUS Upper Extremity Functional Scale (UEFS-P)** We will develop a modified version of the OPUS UEFS to capture the patient perspective on difficulty of activity performance when using a prosthesis. The OPUS UEFS is a component of the Orthotics and Prosthetics User Survey.^52^ The 23- item measure includes activities varying from washing, buttoning shirt, tying shoelaces, using a fork or spoon, and writing name, to donning and doffing the prosthesis.

The OPUS UEFS also asks respondents to indicate whether they usually perform each of the activities using or not using their prosthesis (or orthosis). However, prosthesis use information is not utilized in scoring the original version. The UEFS-P will take prosthesis use into account..

**Patient Experience Measure (PEM):** Our work will refine the PEM, originally developed for use in the ISENS study at Case Western University. [1-3] PEM items are grouped into 5 major domains of enhanced self-efficacy, greater sense of device embodiment, improvements in body image, greater efficiency of the prosthesis, and increased engagement of the prosthesis in social situations. Respondents rate each item within each subscale using a 5 point Likert Scale (strongly disagree to strongly agree). The self-efficacy subscale asks respondents to rate their confidence using the prosthesis to complete 7 items that are typically challenging for prosthesis users. Activities include: carrying a small object, holding someone’s hand while walking, picking up a soda bottle without crushing it, picking up a paper cup without crushing it, carrying a slippery object without dropping it, and shaking hands without hurting or pinching. The embodiment subscale consists of 8 items that ask about prosthetic embodiment (e.g. the prosthesis is a part of me). Respondents will be asked to indicate their level of agreement with statements about the prosthesis being part of themselves, an extension of themselves, using the prosthesis to express themselves, feelings of completeness when wearing the prosthesis, and looking forward to removing the prosthesis. The 9-item body Image subscale asks about impact of the prosthesis on self-image (e.g. when I remove my prosthesis I feel more confident). Items ask about confidence when removing the prosthesis, happiness after removing the prosthesis, looking forward to removing the prosthesis, feelings of differences from others, relief, and shyness in public after removing the prosthesis. The 3-item prosthesis efficiency scale includes items relating to speed and focus required to use the prosthesis. Items ask participants to agree or disagree with statements indicating that the prosthesis requires a lot of focus, is much slower than using the other hand, and whether they avoid using the prosthesis to do things because it slows them down. Finally, the social touch subscale consists of 11 items pertaining to prosthesis use in social interactions. These items relate to comfort holding hands and shaking hands with persons close to them, comfort holding hands and shaking hands with persons they just met, ability to gently squeeze someone else’s hand using the prosthesis, their ability to perceive someone else gently squeezing their hand, using the prosthesis in an embrace, using the prosthesis to convey a friendly touch, to communicate anger, to gently pat a dog or cat, and their ability to control the firmness of their touch when patting someone on the back. Finally, participants are asked to indicate whether the prosthesis interferes with physical and intimate relationships

**Modified Prosthetic Evaluation Questionnaire (PEQ)** The PEQ is a multi-dimensional measure developed for lower limb amputees. ^39^ The PEQ utility and residual limb health subscales, if proven reliable and valid in upper limb amputees, may be particularly useful for quantifying the impact of osseointegration; the utility subscale contains 6 of 8 items that we believe are applicable to the osseointegrated prosthesis. These items ask about the fit of the prosthesis, weight of the prosthesis, comfort while using the prosthesis standing and sitting, the feeling of the prosthesis (temperature, texture on residual limb), and ease of donning. The residual limb health subscale consists of 6 items pertaining to sweating of the residual limb, smell of the prosthesis, swelling of residual limb, rashes on residual limb, ingrown hairs of the residual limb and blisters/sores on the residual limb.

QuickDASH and SF-12 will be administered with the Aim 4 survey.

The demographic, amputation history, and prosthetic use questions from the baseline survey (Aim 1) will be administered to directly recruited participants.

**Patient Reported Outcomes Measurement Information System (PROMIS) Upper Extremity 7 item Short Form and additional items** The Patient Reported Outcomes Measurement Information System (PROMIS®) Upper Extremity Item Bank was developed to assess patient reported upper limb function. Short form versions of the full Item Bank (which contains 29 items) have been developed. The full item bank and short forms have been validated in a variety of patient populations, but have never been examined in amputees.  Given, the national initiatives to utilize PROMIS measures, when possible, we added the 7-item short form to our study so that we could examine its validity in upper limb amputees.  Given that many of the PROMIS items are unilateral activities, which would not require prosthesis use, and the fact that the instructions do not specify which hand would be used to perform activities, we suspected that the PROMIS short form measure would not perform well in our population. Thus, we also added all those items from the full item set that ask about activities that typically require two hands (cut food, use zipper, button shirt, cut paper, tie knot/bow, open milk carton).

**Prosthesis Acceptance Measure** The Prosthetic Acceptance Measure is a new 20 item survey designed for persons with upper limb loss that can be used by both prosthesis users and non-users. The purpose of this scale was to capture attitudes towards prosthesis use to identify barriers and facilitators to use. We utilized findings from our prior literature review, and national survey to identify the content areas for this scale.

## Data Analysis

Item generation: Prior to cognitive testing, we will conduct additional qualitative analyses of responses to data we are currently collecting for the female Veteran survey study (see preliminary study #3) to identify new candidate items for the modified TAPES satisfaction scale (TAPES-MOD), OPUS-CSD-R, OPUS P-UEFS, PEM, and PEQ. Open-ended comments will be transcribed and reviewed independently by two investigators who will scrutinize the data using constant comparative methods to identify aspects of prosthetic satisfaction, self-reported activity difficulty, prosthetic utility and residual limb health. Aspects of each construct identified by the female participants in our study will be compared to the aspects addressed in the existing measures. New items will be generated to address areas not captured by the existing measures. Proposed item wording will be reviewed and refined by the survey methodology team at OSR at the University of Massachusetts.

Cognitive interviewing: We will purposefully select participants to maximize variation, and ensure that the items are relevant and comprehensible for both male and female participants. We expect to conduct 10 interviews focusing on the revised TAPES and CSD, and 10 interviews focused on the UEFS-P and the PEQ. The refined measures will then be pilot tested in a sample of 20 persons, also recruited from completers of the baseline survey. We expect to include at least 4 persons (from each of the following sub-categories: TR amputation, TH amputation, Shoulder level amputation, bilateral amputation, non-prosthesis users. Cognitive interviews will be audio-recorded. Data analysis of cognitive interviews will be an iterative process. The Principal Investigator will review audiotaped interviews several times to identify problematic items, and make modifications to test in subsequent interviews. Thus, items will be iteratively refined and re-administered. This process will continue until no major issues are discovered. If multiple iterations are needed to address issues discovered during pilot testing, we will increase the pilot sample size until we achieve results indicating that the new metrics are ready for wider field testing.

Pilot testing: Findings from cognitive interviewing will be used to refine the new measures which will then be pilot tested with approximately 20 persons. Problematic items will be modified or removed. We believe that this sample size will be adequate, although there are no definitive rules for determining appropriate sample sizes for cognitive interviewing and pilot testing. If multiple iterations are needed to address issues discovered during cognitive or pilot testing, we will increase the sample size until we achieve results indicating that the new metrics are ready for wide field testing. At the conclusion of pilot testing we will have developed two versions of each measure; one for paper and pencil administration and the other for interviewer-administration.

Reliability testing: Once the measure is ready for wider testing, 50 participants will take part in reliability testing. Fifty participants will complete paper and pencil versions twice within 2 weeks, and 50 will complete the telephone interview twice within 2 weeks. Test-retest reliability comparing scores for Visit 1 and Visit 2 will be examined using repeated measures analysis of variance and the Shrout and Fleiss intraclass correlation coefficient (ICC) type 3,1. ICC(3,1) is a two-way mixed model, single measure of reliability.

Calibration analyses: Precision of the measures will be maximized through use of contemporary measurement methods: using calibration data. A Rasch model will be used to place person ability and item difficulty on the same linear continuum, and then item parameters will be calculated. Next, raw-scores of measures will be converted into interval-based measures- the logs-odds metric or logit to generate Rasch based scoring algorithm. If a mode effect or ordering effect is discovered in the mode of administration analyses, we will conduct a sensitivity analysis to determine whether or not to include the 50 participants who completed the telephone survey at Visit 2 in the calibration analyses.

Mode equivalence analyses: The equivalence of interviewer-administered and self-administered versions will be evaluated using ICC (2,1) to evaluate correspondence between modes for item scores and summary scores of each measure. Analyses of Variance (ANOVA’s) with mode order as a covariate will be used to test for the presence of an ordering effect.

Validation We will use data from the calibration sample to examine various aspects of validity, including concurrent, divergent and known group validity. Each validity test and expected results are summarized below.

For concurrent and divergent validity, We will use Pearson’s correlations to examine cross-sectional relationships between the new and modified metrics and existing measures for similar and different constructs. We will utilize Cohen’s conventions to interpret effect size (r=0.10 small, r=0.30 moderate, r=0.5 large).^72^

For known groups validation*,* we will compare outcomes for prosthesis users and nonusers, by amputation level, and by years of experience as a prosthesis user (experienced >1 year, vs. < 1 year). For these analyses, we will use t-tests, ANOVAs or Kruskal-Wallis one-way analysis of variance by ranks test (if there are small numbers for some sub-groups). Prior research demonstrates significant differences in activity performance and self-reported disability by amputation level,^31^ as well as differences in prosthesis satisfaction as evidenced by abandonment by level of amputation. If our new metrics are valid, we expect to see similar patterns in the data. We also hypothesize that there would be significant differences in PEQ residual limb health for prosthesis users and non-users.

Other validity analyses: We will compare PEM scores of persons with and without SRS implantation to test the hypothesis that all PEM subscale scores would be better for upper limb amputees using SRS systems as compared to those without these systems. We will use t-tests or ANOVAs to compare group means, but for small sample sizes we will also use Wilcoxin signed rank tests or Kruskal-Wallis one-way analysis of variance by ranks tests. P-UEFS scores will be compared by amputation level, and years for prosthesis experience. We hypothesize that P-UEFS scores will be lower (indicating worse function) for persons with more proximal amputation (TH, SD, FQ) as compared to TR, and lower for those with less prosthesis use experience. Validity of PEQ will be examined by testing the hypothesis that persons who rate utility and residual limb health more favorably will wear their prostheses more often and for more hours per day. We will develop separate general linear regression models using the hours of prosthesis use and days/week of prosthesis use as independent variables to predict each of the PEQ subscale scores (utility and residual limb health) adjusting for amputation level. We will also perform exploratory analyses to describe PEQ scores for prosthetic users by amputation level for major category of suspensions (suction, gel or silicone liner with pin, vacuum, self-suspending, external suspension sleeve). Validity of PEQ will be examined by testing the hypothesis that persons who rate utility and residual limb health more favorably will wear their prostheses more often and for more hours per day. We will develop separate general linear regression models using the hours of prosthesis use and days/week of prosthesis use as independent variables to predict each of the PEQ subscale scores (utility and residual limb health) adjusting for amputation level. We will also perform exploratory analyses to describe PEQ scores for prosthetic users by amputation level for major category of suspensions (suction, gel or silicone liner with pin, vacuum, self-suspending, external suspension sleeve).

Descriptive analyses comparing outcomes by gender

We will describe outcomes (TAPES, TAPES-MOD, OPUS CSD, OPUS CSD-R, OPUS P-UEFS, PEM, PEQ-utility, PEQ Residual Limb Health) of men and women by amputation level using ANOVAs or Kruskal-Wallis one-way analysis of variance by ranks test, if there are small numbers for sub-groups.

**Sample size considerations:**

a. Item generation and modification of existing measures (N/A)

b. Cognitive and pilot testing: There are no definitive rules for determining appropriate sample sizes for cognitive testing and pilot testing. Conducting cognitive interviews with up to 20 participants (10 per group of measures) affords us the opportunity to test the instruments by gender and amputation level. We considered conducting fewer interviews. However, increased sample size is positively associated with number, type, and severity of problem items identified during the interview process ^75^ Pilot testing will also be conducted with 10 participants per measure group (20 total). In prior studies, we have found that 10 participants provide sufficient diversity in attitudes and experiences to test new items adequately.

c. Test-retest reliability

Our sample will consist of 50 subjects per mode of administration (telephone or paper and pencil). A total of 48 subjects is needed to achieve power of 0.8 under significance level 0.05, to reject the null hypothesis that the true reliability coefficient is excellent (r=0.9) when in fact it is at the minimally acceptable value is good (r= 0.8). ^76^

d. Structural Validity and Rasch modeling

We expect that our calibration sample will be sufficient for both structural validity (factor analyses) and Rasch analyses. While there are no hard and fast rules, a rule of thumb is that the ratio of sample size (N) to number of items in the model (p) should be >10. Our longest scale is the OPUS UEFS-P (with 23 items), thus we exceed this minimum threshold.^77^ For Rasch analyses, a sample ≥250 participants are required for polytomous items to achieve stable and robust estimates of item parameters.^78-80^ However, Scott and colleagues maintain that a sample of 100 is sufficient to estimate item difficulties within 0.5 logits.^79^

e Differential item functioning

The minimum sample size per sub-group needed for DIF analysis is not clear-cut. Others examining DIF in outcome measures for upper limb amputation have performed these analyses in sub-groups as small as 50 persons. With 100 per group, a large DIF effect can be detected using an ordinal logistic regression.^81^ Further, a 100 person sample will have 80% power to detect at least an item calibration difference of 0.4 logits at 80% power using paired t-tests of item calibration between groups.

f. Validation

f.1-f.2 Concurrent validity-and divergent correlations

With a sample of 286 we would have 80% power to detect concurrent validity correlations as low as 0.165 with an alpha of 0.5 (calculated using G-Power 3.1.9.2).

f. 3 Known group validation and g. comparison by sex

| **Analysis** | **Sample size** | **Effect size** | **Power** |
| --- | --- | --- | --- |
| ANOVAs | 300 | .181 | 80% |
| Kruskal-Wallis | 255 | .196 | 80% |
| **Table 8. Sample size calculation for known groups validation and comparisons by sex** | | | |

We calculated (using G Power 3.1.9.2) the power that we would achieve, given alphas of .05 for group comparison. For estimates of non-parametric tests (Kruskal-Wallis and Friedman tests) where the distribution of values is not known, we assumed that effect sizes and power achieved would be equivalent to what would be achievable with 15% fewer subjects.^74^ The calculations shown in Table 8 demonstrate that a sample of 300 persons provides ample power to achieve the study objectives.

## Withdrawal of Subjects

Subjects have the right to withdraw from the study at any time by notifying the Principal Investigator or designee of the Principal Investigator (ie, research assistant/study coordinator) orally or in writing. Thus, subjects are free to terminate the telephone survey at any time. Subjects also are free to withdraw from the study after completing Part 1, and will be informed that they do not have to continue on to participate in Parts 2 or 3 (if selected).

# Reporting

The Principal Investigator will have primary responsibility for day-to-day monitoring of participant safety, and the quality of operations in all data collection, data cleaning and transfer activities. The research protocol will be approved by the following Institutional Review Boards: VA Central IRB, Center for the Intrepid, University of Massachusetts Medical School, Food and Drug Administration, and USAMRMC ORP Human Research Protections Office before any data collection begins.

All Adverse Events (AEs) will be assessed continuously and reported to the local PIs immediately. The PVAMC study team will also be notified of all AEs that take place at each participating study sites.

Adverse Events and unanticipated events will be submitted to each participating IRB according to local policy.

**Special reporting guidelines for VA Central IRB**
The VA Central IRB requires reporting of all local unanticipated serious adverse events and unanticipated problems involving risks to participants or others that are related to research projects overseen by the VA Central IRB:

(1) Immediate oral reporting if all research-related deaths is required, followed by written notification within 5 work days after being made award of the death.

(2) Written notification within 5 work days after being made aware of the occurrence is required for all qualifying incidents. Local site investigators are not required to report events and problems to the VA central IRB that do not occur at their site.

(3) Adverse events or problems that do not meet the criteria for reporting are to be reported in summary format by the investigator as part of the continuing review submission for a study or as part of a study or site closure report.

The VA Central IRB reviews all reported unanticipated serious adverse events and serious unanticipated problems involving risks to participants or others and determines if they require further reporting. If the submitted report requires further reporting, the VA Central IRB will report the incident to the VA Central IRB Institutional Official and the applicable local VSA Medical Facility Director and ACOS/R&D in accordance with VA Central IRB SOP 125, Reportable Action Reporting. SOP 125 also includes reporting requirements to the VA Office of Research Oversight (ORO), the Office for Human Research Protections (OHRP), and/or the US Food and Drug Administration as applicable.

Privacy and Confidentiality

The study will collect and utilize Protected Health Information (PHI). All participating study staff will be trained in HIPAA and Data Security policies and will maintain up-to-date training required for their institutions.

Electronic Data:

A password protected electronic file with participant names and their subject ID numbers will be stored in Dr. Resnik’s secure research folder. Survey data will be merged with diagnostic information and other data extracted from VA datasets and will be stored in VINCI. To ensure the protection of Veteran data, VINCI maintains compliance with the guidelines set forth by Veterans Health Administration (VHA) Handbook 1200.12, Use of Data and Data Repositories in VHA Research, and all other applicable VA and VHA policies and regulations. In addition, VINCI has undergone all security certification activities in support of obtaining an Authorization to Operate (ATO). Access to VINCI resources are approved in accordance with the requirements of National Data Systems (NDS), VHA Handbook 1200.12, Use of Data and Data Repositories in VHA Research, and all other applicable VA and VHA policies and regulations. All data transferred from VINCI is subject to audit for compliance.

Paper Data:

Response cards and completed paper and pencil surveys, will be stored in locked files in a private locked office at PVAMC. Files will only be accessible by approved study team members.

Telephone Surveys:

Data from the telephone surveys will be stored in the AWS HIPAA compliant VPC (Amazon Web Services HIPAA compliant Virtual Private Cloud). Data will be entered into REDCap. Data will be protected in the following ways:

- Access to the REDCap web site will be encrypted via SSL from client to server.
- Access to the REDCap application itself will be managed by a login challenge.  Only those individuals granted access to the application will be able to log in and have rights limited to the permissions granted by the REDCap Administrator.
- Only study team members will be granted access to the Virtual work station.  Access will be controlled by a two phase authentication first to have access to the workstation hub and second to the workstation itself.
- Data will be delivered to the PVAMC team via VA approved secure method.

At completion of the project when the data are no longer required, all instances of EC2 servers, VM workstations and storage will be terminated and destroyed in accordance with AWS HIPAA compliance. Additional security measures will be added as necessary to comply with all VA directives developed in accordance with FISMA, HIPAA, NIST, and related VA security and privacy control requirements for Federal information systems.

Data from telephone surveys collected by the PVAMC study team will be entered into REDCap and then extracted and stored in VINCI. See above for detail regarding data storage on VINCI.

Data will be transferred to the University of Massachusetts Survey Center as previously outlined in this protocol, per the approved Data Use Agreement.

# Communication Plan

All local site IRB and R&D approvals will be obtained prior to initiating this research study. All engaged sites will receive notification(s) when there are changes to the study protocol. All unanticipated events will be reported as per protocol section 6.0. No research will be conducted at any facility that is not engaged. Study staff will receive training, through online and/or in-person training sessions prior to engaging in study activities to ensure that the research is conducted according to IRB-approved protocol. A minimum of monthly telephone meetings will be held with key study staff during the data collection phase.

# References

1.	Ziegler-Graham K, MacKenzie EJ, Ephraim PL, Travison TG, Brookmeyer R. Estimating the Prevalence of Limb Loss in the United States: 2005 to 2050. *Archives of Physical Medicine and Rehabilitation.* 2008;89(3):422-429.

2.	Petri RP, Jr., Aguila E. The military upper extremity amputee. *Phys Med Rehabil Clin N Am.* 2002;13(1):17-43.

3.	Stansbury LG, Lalliss SJ, Branstetter JG, Bagg MR, Holcomb JB. Amputations in U.S. military personnel in the current conflicts in Afghanistan and Iraq. *J Orthop Trauma.* 2008;22(1):43-46.

4.	Matsen SL, Malchow D, Matsen FA, 3rd. Correlations with patients' perspectives of the result of lower-extremity amputation. *J Bone Joint Surg Am.* 2000;82-A(8):1089-1095.

5.	Hanley MA, Ehde DM, Jensen M, Czerniecki J, Smith DG, Robinson LR. Chronic pain associated with upper-limb loss. *American Journal of Physical Medicine & Rehabilitation.* 2009;88(9):742.

6.	Rock Gambrell C. Overuse Syndrome and the Unilateral Upper Limb Amputee: Consequences and Prevention. *Journal of Prosthetics & Orthotics (JPO).* 2008;20(3):126-132.

7.	Kejlaa GH. Consumer concerns and the functional value of prostheses to upper limb amputees. *Prosthet Orthot Int.* 1993;17(3):157-163.

8.	Atkins DJ, Heard DC, Donovan WH. Epidemiologic Overview of lndividuals with Upper-Limb Loss and Their Reported Research Priorities. *Journal of Prosthetics & Orthotics.* 1996;8(1):0.

9.	Reyburn TV. A method of early prosthetics training for upper-extremity amputees. *Artif Limbs.* 1971;15(2):1-5.

10.	Biddiss E, Beaton D, Chau T. Consumer design priorities for upper limb prosthetics. *Disabil Rehabil Assist Technol.* 2007;2(6):346-357.

11.	Wright TW, Hagen AD, Wood MB. Prosthetic usage in major upper extremity amputations. *J Hand Surg [Am].* 1995;20(4):619-622.

12.	Biddiss E, Chau T. Upper-limb prosthetics: critical factors in device abandonment. *Am J Phys Med Rehabil.* 2007;86(12):977-987.

13.	McFarland LV, Winkler SLH, Heinemann AW, Jones M, Esquenazi A. Unilateral upper-limb loss: Satisfaction and prosthetic-device use in veterans and servicemembers from Vietnam and OIF/OEF conflicts. *Journal of Rehabilitation Research & Development.* 2010;47(3):299-316.

14.	Blough DK, Hubbard S, McFarland LV, Smith DG, Gambel JM, Reiber GE. Prosthetic cost projections for servicemembers with major limb loss from Vietnam and OIF/OEF. *J Rehabil Res Dev.* 2010;47(4):387-402.

15.	Resnik L, Borgia M. Reliability and Validity of Outcome Measures for Upper Limb Amputation. *JPO: Journal of Prosthetics and Orthotics.* 2012;24(4):192-212

16.	Ahern D, Correia, S., Resnik, L., Barnabe, K., & Gnepp, D. *Cognitive Predictors of Successful Adaptation to an Advanced Multifunction Prosthesis.* 6 ed. GREAT CLARENDON ST, OXFORD OX2 6DP, ENGLAND:: Oxford University Press (OUP); 2012.

17.	Resnik L, Klinger SL, Etter K. The DEKA Arm: its features, functionality, and evolution during the Veterans Affairs Study to optimize the DEKA Arm. *Prosthet Orthot Int.* 2014;38(6):492-504.

18.	Baker JJ, Scheme E, Englehart K, Hutchinson DT, Greger B. Continuous detection and decoding of dexterous finger flexions with implantable myoelectric sensors. *IEEE Trans Neural Syst Rehabil Eng.* 2010;18(4):424-432.

19.	Ortiz-Catalan M, Branemark R, Hakansson B, Delbeke J. On the viability of implantable electrodes for the natural control of artificial limbs: Review and discussion. *Biomed Eng Online.* 2012;11:33.

20.	Horch K, Meek S, Taylor TG, Hutchinson DT. Object discrimination with an artificial hand using electrical stimulation of peripheral tactile and proprioceptive pathways with intrafascicular electrodes. *IEEE Trans Neural Syst Rehabil Eng.* 2011;19(5):483-489.

21.	Raspopovic S, Capogrosso M, Petrini FM, et al. Restoring natural sensory feedback in real-time bidirectional hand prostheses. *Science translational medicine.* 2014;6(222):222ra219.

22.	Tan DW, Schiefer MA, Keith MW, Anderson JR, Tyler J, Tyler DJ. A neural interface provides long-term stable natural touch perception. *Science translational medicine.* 2014;6(257):257ra138.

23.	Ho MP, Gonzalez JM, Lerner HP, et al. Incorporating patient-preference evidence into regulatory decision making. *Surg Endosc.* 2015.

24.	FDA. News Release. FDA approvdes first-of-kind device to treat obesity. January 14,, 2015.

25.	Berke GM, Fergason J, Milani JR, et al. Comparison of satisfaction with current prosthetic care in veterans and servicemembers from Vietnam and OIF/OEF conflicts with major traumatic limb loss. *Journal of Rehabilitation Research & Development.* 2010;47(4):361-372.

26.	Management of Upper Extremity Amputation Rehabilitation Working Group. VA/DoD Clinical Practice Guideline for the Management of Upper Extremity Amputation Rehabilitation. Department of Veterans Affairs, Department of Defense.; 2014.

27.	Resnik LJ, Borgia ML. Factors associated with utilization of preoperative and postoperative rehabilitation services by patients with amputation in the VA system: an observational study. *Phys Ther.* 2013;93(9):1197-1210.

28.	Zhou J, Bates BE, Kurichi JE, Kwong PL, Xie D, Stineman MG. Factors influencing receipt of outpatient rehabilitation services among veterans following lower extremity amputation. *Arch Phys Med Rehabil.* 2011;92(9):1455-1461.

29.	Etter K, Borgia M, Resnik L. Prescription and repair rates of prosthetic limbs in the VA healthcare system: implications for national prosthetic parity. *Disability and Rehabilitation: Assistive Technology.*0(0):1-8.

30.	Resnik L, Baxter K, Borgia M, Mathewson K. Is the UNB test reliable and valid for use with adults with upper limb amputation? *J Hand Ther.* 2013;26(4):353-359; quiz 359.

31.	Resnik L, Borgia M. Reliability, Validity and Responsiveness of the QuickDASH in Patients with Upper Limb Amputation. *Arch Phys Med Rehabil.* 2015.

32.	Resnik L, Borgia M. Responsiveness of outcome measures for upper limb prosthetic rehabilitation. *Prosthet Orthot Int.* 2014.

33.	Resnik L. Research update: VA study to optimize DEKA arm. *J Rehabil Res Dev.* 2010;47(3):ix-x.

34.	Resnik L, Borgia M. User ratings of prosthetic usability and satisfaction in VA study to optimize DEKA arm. *J Rehabil Res Dev.* 2014;51(1):15-26.

35.	Phillips SL, Resnik L, Fantini C, Latlief G. Endpoint Control for a Powered Shoulder Prosthesis. *Journal of Posthetics & Othotics.* 2013;25(4):8.

36.	Resnik L, Borgia M, Latlief G, Sasson N, Smurr-Walters L. Self-reported and performance-based outcomes using DEKA Arm. *J Rehabil Res Dev.* 2014;51(3):351-362.

37.	Resnik L, Klinger SL, Etter K, Fantini C. Controlling a multi-degree of freedom upper limb prosthesis using foot controls: user experience. *Disabil Rehabil Assist Technol.* 2013.

38.	Smith DG, Horn P, Malchow D, Boone DA, Reiber GE, Hansen ST, Jr. Prosthetic history, prosthetic charges, and functional outcome of the isolated, traumatic below-knee amputee. *J Trauma.* 1995;38(1):44-47.

39.	Legro MW, Reiber GD, Smith DG, del Aguila M, Larsen J, Boone D. Prosthesis evaluation questionnaire for persons with lower limb amputations: assessing prosthesis-related quality of life. *Arch Phys Med Rehabil.* 1998;79(8):931-938.

40.	Legro MW, Reiber G, del Aguila M, et al. Issues of importance reported by persons with lower limb amputations and prostheses. *J Rehabil Res Dev.* 1999;36(3):155-163.

41.	Smith DG, Ehde DM, Legro MW, Reiber GE, del Aguila M, Boone DA. Phantom limb, residual limb, and back pain after lower extremity amputations. *Clin Orthop.* 1999(361):29-38.

42.	Highsmith MJ, Kahle JT, Knight M, Olk-Szost A, Boyd M, Miro RM. Delivery of cosmetic covers to persons with transtibial and transfemoral amputations in an outpatient prosthetic practice. *Prosthetics and Orthotics International.* 2015.

43.	Highsmith MJ, Carey SL, Koelsch KW, Lusk CP, Maitland ME. Kinematic Evaluation of Terminal Devices for Kayaking With Upper Extremity Amputation. *JPO: Journal of Prosthetics and Orthotics.* 2007;19(3):84-90 10.1097/JPO.1090b1013e31806ada31802f.

44.	Carey SL, Jason Highsmith M, Maitland ME, Dubey RV. Compensatory movements of transradial prosthesis users during common tasks. *Clin Biomech (Bristol, Avon).* 2008;23(9):1128-1135.

45.	Carey SL, Dubey RV, Bauer GS, Highsmith MJ. Kinematic comparison of myoelectric and body powered prostheses while performing common activities. *Prosthetics and Orthotics International.* 2009;33(2):179-186.

46.	Ramirez IA, Lusk CP, Dubey R, Highsmith MJ, Maitland ME. Crossed four-bar mechanism for improved prosthetic grasp. *J Rehabil Res Dev.* 2009;46(8):1011-1020.

47.	Resnik LJ, Clark MA, Borgia M. Telephone and face to face methods of assessment of veteran's community reintegration yield equivalent results. *BMC Med Res Methodol.* 2011;11(1):98.

48.	Resnik L, Plow M, Jette A. Development of CRIS: measure of community reintegration of injured service members. *J Rehabil Res Dev.* 2009;46(4):469-480.

49.	Ware JE, Jr., Snow K, Kozinski M, Gandek B. *SF-36 Health Survey: Manual and Interpretation Guide.* Boston: The Health Institute New England Medical Center; 1993.

50.	Beaton DE, Wright JG, Katz JN. Development of the QuickDASH: comparison of three item-reduction approaches. *J Bone Joint Surg Am.* 2005;87(5):1038-1046.

51.	Desmond DM, MacLachlan M. Factor structure of the Trinity Amputation and Prosthesis Experience Scales (TAPES) with individuals with acquired upper limb amputations. *Am J Phys Med Rehabil.* 2005;84(7):506-513.

52.	Heinemann AW, Bode RK, O'Reilly C. Development and measurement properties of the Orthotics and Prosthetics Users' Survey (OPUS): a comprehensive set of clinical outcome instruments. *Prosthet Orthot Int.* 2003;27(3):191-206.

53.	Gummesson C, Ward MM, Atroshi I. The shortened disabilities of the arm, shoulder and hand questionnaire (QuickDASH): validity and reliability based on responses within the full-length DASH. *BMC Musculoskelet Disord.* 2006;7:44.

54.	Mittal VK, Pierce AK, Priestley JC. Use of probenecid in ticarcillin-induced hypokalemia. *Clin Ther.* 1980;3(2):125-128.

55.	Ware J, Jr., Kosinski M, Keller SD. A 12-Item Short-Form Health Survey: construction of scales and preliminary tests of reliability and validity. *Med Care.* 1996;34(3):220-233.

56.	Ware JE, Jr. SF-36 health survey update. *Spine.* 2000;25(24):3130-3139.

57.	Resnik L, Borgia M. Reliability of outcome measures for people with lower-limb amputations: distinguishing true change from statistical error. *Phys Ther.* 2011;91(4):555-565.

58.	VA. *PSAS Program Evaluation: Patients With Amputations Study.* December 3, 2002 2002.

59.	Rider B, Linden C. Comparison of standardized and non-standardized administration of the Jebsen Hand Function Test. *Journal of Hand Therapy.* 1988;2:121-123.

60.	Burckhardt CS, Anderson KL. The Quality of Life Scale (QOLS): reliability, validity, and utilization. *Health Qual Life Outcomes.* 2003;1:60.

61.	Meredith JM. Comparison of three myoelectrically controlled prehensors and the voluntary-opening split hook. *Am J Occup Ther.* 1994;48(10):932-937.

62.	Wang YC, Magasi SR, Bohannon RW, et al. Assessing dexterity function: a comparison of two alternatives for the NIH Toolbox. *J Hand Ther.* 2011;24(4):313-320; quiz 321.

63.	Mathiowetz V, Volland G, Kashman N, Weber K. Adult norms for the Box and Block Test of manual dexterity. *Am J Occup Ther.* 1985;39(6):386-391.

64.	Resnik L, Adams L, Borgia M, et al. Development and Evaluation of the Activities Measure for Upper Limb Amputees. *Arch Phys Med Rehabil.* 2012.

65.	Resnik L, Borgia M, Acluche F. Brief activity performance measure for upper limb amputees: BAM-ULA. *Prosthet Orthot Int.* 2017:309364616684196.

66.	Resnik L, Borgia M, Acluche F. Timed activity performance in persons with upper limb amputation: A preliminary study. *J Hand Ther.* 2017.

67.	Kazis LE, Miller DR, Skinner KM, et al. Patient-reported measures of health: The Veterans Health Study. *J Ambul Care Manage.* 2004;27(1):70-83.

68.	Resnik L, Borgia M, Ni P, Pirraglia PA, Jette A. Reliability, validity and administrative burden of the community reintegration of injured service members computer adaptive test (CRIS-CAT)". *BMC Med Res Methodol.* 2012;12(1):145.

69.	World Health Organization. *International Classification of Functioning, Disability, and Health* Geneva: World Health Organization;2001.

70.	Gallagher P, Maclachlan M. The Trinity Amputation and Prosthesis Experience Scales and quality of life in people with lower-limb amputation. *Arch Phys Med Rehabil.* 2004;85(5):730-736.

71.	Gallagher P, MacLachlan M. Development and psychometric evaluation of the Trinity Amputation and Prosthesis Experience Scales (TAPES). *Rehabilitation Psychology.* 2000;45(2):130-154.

72.	Cohen J. *Statistical Power Analysis for the Behavioral Sciences.* New Jersey: Lawrence Elrbaum Associates; 1988.

73.	Holm S. A simple sequentially rejective multiple test procedure. *Scandinavian Journal of Statistics.* 1979;6(2):70.

74.	Lehmann E. *Nonparametrics : Statistical Methods Based on Ranks* New York: Springer New York; 1998.

75.	Blair JM, Conrad F. Sample Size for Cognitive Interview Pretesting. *Public Opinion Quarterly.* 2011;75(4): 636–658.

76.	Donner A, Eljaszw M. Sample size requirements for reliability studies. . *Statistics in medicine* 1987;6(4):441-448.

77.	Myers ND, Ahn S, Jin Y. Sample size and power estimates for a confirmatory factor analytic model in exercise and sport: a Monte Carlo approach. *Res Q Exerc Sport.* 2011;82(3):412-423.

78.	Linacre JM. Optimizing rating scale category effectiveness. *J Appl Meas.* 2002;3(1):85-106.

79.	Linacre M. Sample size and item calibration stability. *Rasch Measurement Transactions.* 1994;7:326.

80.	Chen WH, Lenderking W, Jin Y, Wyrwich KW, Gelhorn H, Revicki DA. Is Rasch model analysis applicable in small sample size pilot studies for assessing item characteristics? An example using PROMIS pain behavior item bank data. . *Quality of Life Research.* 2014;23(2): 485-493.

81.	Scott NW, Fayers PM, Aaronson NK, et al. A simulation study provided sample size guidance for differential item functioning (DIF) studies using short scales. *J Clin Epidemiol.* 2009;62(3):288-295.
